# Fiducial, total and differential cross-section measurements of *t*-channel single top-quark production in *pp* collisions at 8 TeV using data collected by the ATLAS detector

**DOI:** 10.1140/epjc/s10052-017-5061-9

**Published:** 2017-08-09

**Authors:** M. Aaboud, G. Aad, B. Abbott, J. Abdallah, O. Abdinov, B. Abeloos, O. S. AbouZeid, N. L. Abraham, H. Abramowicz, H. Abreu, R. Abreu, Y. Abulaiti, B. S. Acharya, S. Adachi, L. Adamczyk, D. L. Adams, J. Adelman, S. Adomeit, T. Adye, A. A. Affolder, T. Agatonovic-Jovin, J. A. Aguilar-Saavedra, S. P. Ahlen, F. Ahmadov, G. Aielli, H. Akerstedt, T. P. A. Åkesson, A. V. Akimov, G. L. Alberghi, J. Albert, S. Albrand, M. J. Alconada Verzini, M. Aleksa, I. N. Aleksandrov, C. Alexa, G. Alexander, T. Alexopoulos, M. Alhroob, B. Ali, M. Aliev, G. Alimonti, J. Alison, S. P. Alkire, B. M. M. Allbrooke, B. W. Allen, P. P. Allport, A. Aloisio, A. Alonso, F. Alonso, C. Alpigiani, A. A. Alshehri, M. Alstaty, B. Alvarez Gonzalez, D. Álvarez Piqueras, M. G. Alviggi, B. T. Amadio, Y. Amaral Coutinho, C. Amelung, D. Amidei, S. P. Amor Dos Santos, A. Amorim, S. Amoroso, G. Amundsen, C. Anastopoulos, L. S. Ancu, N. Andari, T. Andeen, C. F. Anders, J. K. Anders, K. J. Anderson, A. Andreazza, V. Andrei, S. Angelidakis, I. Angelozzi, A. Angerami, F. Anghinolfi, A. V. Anisenkov, N. Anjos, A. Annovi, C. Antel, M. Antonelli, A. Antonov, D. J. Antrim, F. Anulli, M. Aoki, L. Aperio Bella, G. Arabidze, Y. Arai, J. P. Araque, A. T. H. Arce, F. A. Arduh, J-F. Arguin, S. Argyropoulos, M. Arik, A. J. Armbruster, L. J. Armitage, O. Arnaez, H. Arnold, M. Arratia, O. Arslan, A. Artamonov, G. Artoni, S. Artz, S. Asai, N. Asbah, A. Ashkenazi, B. Åsman, L. Asquith, K. Assamagan, R. Astalos, M. Atkinson, N. B. Atlay, K. Augsten, G. Avolio, B. Axen, M. K. Ayoub, G. Azuelos, M. A. Baak, A. E. Baas, M. J. Baca, H. Bachacou, K. Bachas, M. Backes, M. Backhaus, P. Bagiacchi, P. Bagnaia, Y. Bai, J. T. Baines, M. Bajic, O. K. Baker, E. M. Baldin, P. Balek, T. Balestri, F. Balli, W. K. Balunas, E. Banas, Sw. Banerjee, A. A. E. Bannoura, L. Barak, E. L. Barberio, D. Barberis, M. Barbero, T. Barillari, M-S. Barisits, T. Barklow, N. Barlow, S. L. Barnes, B. M. Barnett, R. M. Barnett, Z. Barnovska-Blenessy, A. Baroncelli, G. Barone, A. J. Barr, L. Barranco Navarro, F. Barreiro, J. Barreiro Guimarães da Costa, R. Bartoldus, A. E. Barton, P. Bartos, A. Basalaev, A. Bassalat, R. L. Bates, S. J. Batista, J. R. Batley, M. Battaglia, M. Bauce, F. Bauer, H. S. Bawa, J. B. Beacham, M. D. Beattie, T. Beau, P. H. Beauchemin, P. Bechtle, H. P. Beck, K. Becker, M. Becker, M. Beckingham, C. Becot, A. J. Beddall, A. Beddall, V. A. Bednyakov, M. Bedognetti, C. P. Bee, L. J. Beemster, T. A. Beermann, M. Begel, J. K. Behr, A. S. Bell, G. Bella, L. Bellagamba, A. Bellerive, M. Bellomo, K. Belotskiy, O. Beltramello, N. L. Belyaev, O. Benary, D. Benchekroun, M. Bender, K. Bendtz, N. Benekos, Y. Benhammou, E. Benhar Noccioli, J. Benitez, D. P. Benjamin, J. R. Bensinger, S. Bentvelsen, L. Beresford, M. Beretta, D. Berge, E. Bergeaas Kuutmann, N. Berger, J. Beringer, S. Berlendis, N. R. Bernard, C. Bernius, F. U. Bernlochner, T. Berry, P. Berta, C. Bertella, G. Bertoli, F. Bertolucci, I. A. Bertram, C. Bertsche, D. Bertsche, G. J. Besjes, O. Bessidskaia Bylund, M. Bessner, N. Besson, C. Betancourt, A. Bethani, S. Bethke, A. J. Bevan, R. M. Bianchi, M. Bianco, O. Biebel, D. Biedermann, R. Bielski, N. V. Biesuz, M. Biglietti, J. Bilbao De Mendizabal, T. R. V. Billoud, H. Bilokon, M. Bindi, A. Bingul, C. Bini, S. Biondi, T. Bisanz, D. M. Bjergaard, C. W. Black, J. E. Black, K. M. Black, D. Blackburn, R. E. Blair, T. Blazek, I. Bloch, C. Blocker, A. Blue, W. Blum, U. Blumenschein, S. Blunier, G. J. Bobbink, V. S. Bobrovnikov, S. S. Bocchetta, A. Bocci, C. Bock, M. Boehler, D. Boerner, J. A. Bogaerts, D. Bogavac, A. G. Bogdanchikov, C. Bohm, V. Boisvert, P. Bokan, T. Bold, A. S. Boldyrev, M. Bomben, M. Bona, M. Boonekamp, A. Borisov, G. Borissov, J. Bortfeldt, D. Bortoletto, V. Bortolotto, K. Bos, D. Boscherini, M. Bosman, J. D. Bossio Sola, J. Boudreau, J. Bouffard, E. V. Bouhova-Thacker, D. Boumediene, C. Bourdarios, S. K. Boutle, A. Boveia, J. Boyd, I. R. Boyko, J. Bracinik, A. Brandt, G. Brandt, O. Brandt, U. Bratzler, B. Brau, J. E. Brau, W. D. Breaden Madden, K. Brendlinger, A. J. Brennan, L. Brenner, R. Brenner, S. Bressler, T. M. Bristow, D. Britton, D. Britzger, F. M. Brochu, I. Brock, R. Brock, G. Brooijmans, T. Brooks, W. K. Brooks, J. Brosamer, E. Brost, J. H Broughton, P. A. Bruckman de Renstrom, D. Bruncko, R. Bruneliere, A. Bruni, G. Bruni, L. S. Bruni, BH Brunt, M. Bruschi, N. Bruscino, P. Bryant, L. Bryngemark, T. Buanes, Q. Buat, P. Buchholz, A. G. Buckley, I. A. Budagov, F. Buehrer, M. K. Bugge, O. Bulekov, D. Bullock, H. Burckhart, S. Burdin, C. D. Burgard, A. M. Burger, B. Burghgrave, K. Burka, S. Burke, I. Burmeister, J. T. P. Burr, E. Busato, D. Büscher, V. Büscher, P. Bussey, J. M. Butler, C. M. Buttar, J. M. Butterworth, P. Butti, W. Buttinger, A. Buzatu, A. R. Buzykaev, S. Cabrera Urbán, D. Caforio, V. M. Cairo, O. Cakir, N. Calace, P. Calafiura, A. Calandri, G. Calderini, P. Calfayan, G. Callea, L. P. Caloba, S. Calvente Lopez, D. Calvet, S. Calvet, T. P. Calvet, R. Camacho Toro, S. Camarda, P. Camarri, D. Cameron, R. Caminal Armadans, C. Camincher, S. Campana, M. Campanelli, A. Camplani, A. Campoverde, V. Canale, A. Canepa, M. Cano Bret, J. Cantero, T. Cao, M. D. M. Capeans Garrido, I. Caprini, M. Caprini, M. Capua, R. M. Carbone, R. Cardarelli, F. Cardillo, I. Carli, T. Carli, G. Carlino, B. T. Carlson, L. Carminati, R. M. D. Carney, S. Caron, E. Carquin, G. D. Carrillo-Montoya, J. R. Carter, J. Carvalho, D. Casadei, M. P. Casado, M. Casolino, D. W. Casper, E. Castaneda-Miranda, R. Castelijn, A. Castelli, V. Castillo Gimenez, N. F. Castro, A. Catinaccio, J. R. Catmore, A. Cattai, J. Caudron, V. Cavaliere, E. Cavallaro, D. Cavalli, M. Cavalli-Sforza, V. Cavasinni, F. Ceradini, L. Cerda Alberich, A. S. Cerqueira, A. Cerri, L. Cerrito, F. Cerutti, A. Cervelli, S. A. Cetin, A. Chafaq, D. Chakraborty, S. K. Chan, Y. L. Chan, P. Chang, J. D. Chapman, D. G. Charlton, A. Chatterjee, C. C. Chau, C. A. Chavez Barajas, S. Che, S. Cheatham, A. Chegwidden, S. Chekanov, S. V. Chekulaev, G. A. Chelkov, M. A. Chelstowska, C. Chen, H. Chen, S. Chen, S. Chen, X. Chen, Y. Chen, H. C. Cheng, H. J Cheng, Y. Cheng, A. Cheplakov, E. Cheremushkina, R. Cherkaoui El Moursli, V. Chernyatin, E. Cheu, L. Chevalier, V. Chiarella, G. Chiarelli, G. Chiodini, A. S. Chisholm, A. Chitan, M. V. Chizhov, K. Choi, A. R. Chomont, S. Chouridou, B. K. B. Chow, V. Christodoulou, D. Chromek-Burckhart, J. Chudoba, A. J. Chuinard, J. J. Chwastowski, L. Chytka, G. Ciapetti, A. K. Ciftci, D. Cinca, V. Cindro, I. A. Cioara, C. Ciocca, A. Ciocio, F. Cirotto, Z. H. Citron, M. Citterio, M. Ciubancan, A. Clark, B. L. Clark, M. R. Clark, P. J. Clark, R. N. Clarke, C. Clement, Y. Coadou, M. Cobal, A. Coccaro, J. Cochran, L. Colasurdo, B. Cole, A. P. Colijn, J. Collot, T. Colombo, P. Conde Muiño, E. Coniavitis, S. H. Connell, I. A. Connelly, V. Consorti, S. Constantinescu, G. Conti, F. Conventi, M. Cooke, B. D. Cooper, A. M. Cooper-Sarkar, F. Cormier, K. J. R. Cormier, T. Cornelissen, M. Corradi, F. Corriveau, A. Cortes-Gonzalez, G. Cortiana, G. Costa, M. J. Costa, D. Costanzo, G. Cottin, G. Cowan, B. E. Cox, K. Cranmer, S. J. Crawley, G. Cree, S. Crépé-Renaudin, F. Crescioli, W. A. Cribbs, M. Crispin Ortuzar, M. Cristinziani, V. Croft, G. Crosetti, A. Cueto, T. Cuhadar Donszelmann, J. Cummings, M. Curatolo, J. Cúth, H. Czirr, P. Czodrowski, G. D’amen, S. D’Auria, M. D’Onofrio, M. J. Da Cunha Sargedas De Sousa, C. Da Via, W. Dabrowski, T. Dado, T. Dai, O. Dale, F. Dallaire, C. Dallapiccola, M. Dam, J. R. Dandoy, N. P. Dang, A. C. Daniells, N. S. Dann, M. Danninger, M. Dano Hoffmann, V. Dao, G. Darbo, S. Darmora, J. Dassoulas, A. Dattagupta, W. Davey, C. David, T. Davidek, M. Davies, P. Davison, E. Dawe, I. Dawson, K. De, R. de Asmundis, A. De Benedetti, S. De Castro, S. De Cecco, N. De Groot, P. de Jong, H. De la Torre, F. De Lorenzi, A. De Maria, D. De Pedis, A. De Salvo, U. De Sanctis, A. De Santo, J. B. De Vivie De Regie, W. J. Dearnaley, R. Debbe, C. Debenedetti, D. V. Dedovich, N. Dehghanian, I. Deigaard, M. Del Gaudio, J. Del Peso, T. Del Prete, D. Delgove, F. Deliot, C. M. Delitzsch, A. Dell’Acqua, L. Dell’Asta, M. Dell’Orso, M. Della Pietra, D. della Volpe, M. Delmastro, P. A. Delsart, D. A. DeMarco, S. Demers, M. Demichev, A. Demilly, S. P. Denisov, D. Denysiuk, D. Derendarz, J. E. Derkaoui, F. Derue, P. Dervan, K. Desch, C. Deterre, K. Dette, P. O. Deviveiros, A. Dewhurst, S. Dhaliwal, A. Di Ciaccio, L. Di Ciaccio, W. K. Di Clemente, C. Di Donato, A. Di Girolamo, B. Di Girolamo, B. Di Micco, R. Di Nardo, K. F. Di Petrillo, A. Di Simone, R. Di Sipio, D. Di Valentino, C. Diaconu, M. Diamond, F. A. Dias, M. A. Diaz, E. B. Diehl, J. Dietrich, S. Díez Cornell, A. Dimitrievska, J. Dingfelder, P. Dita, S. Dita, F. Dittus, F. Djama, T. Djobava, J. I. Djuvsland, M. A. B. do Vale, D. Dobos, M. Dobre, C. Doglioni, J. Dolejsi, Z. Dolezal, M. Donadelli, S. Donati, P. Dondero, J. Donini, J. Dopke, A. Doria, M. T. Dova, A. T. Doyle, E. Drechsler, M. Dris, Y. Du, J. Duarte-Campderros, E. Duchovni, G. Duckeck, O. A. Ducu, D. Duda, A. Dudarev, A. Chr. Dudder, E. M. Duffield, L. Duflot, M. Dührssen, M. Dumancic, A. K. Duncan, M. Dunford, H. Duran Yildiz, M. Düren, A. Durglishvili, D. Duschinger, B. Dutta, M. Dyndal, C. Eckardt, K. M. Ecker, R. C. Edgar, N. C. Edwards, T. Eifert, G. Eigen, K. Einsweiler, T. Ekelof, M. El Kacimi, V. Ellajosyula, M. Ellert, S. Elles, F. Ellinghaus, A. A. Elliot, N. Ellis, J. Elmsheuser, M. Elsing, D. Emeliyanov, Y. Enari, O. C. Endner, J. S. Ennis, J. Erdmann, A. Ereditato, G. Ernis, J. Ernst, M. Ernst, S. Errede, E. Ertel, M. Escalier, H. Esch, C. Escobar, B. Esposito, A. I. Etienvre, E. Etzion, H. Evans, A. Ezhilov, M. Ezzi, F. Fabbri, L. Fabbri, G. Facini, R. M. Fakhrutdinov, S. Falciano, R. J. Falla, J. Faltova, Y. Fang, M. Fanti, A. Farbin, A. Farilla, C. Farina, E. M. Farina, T. Farooque, S. Farrell, S. M. Farrington, P. Farthouat, F. Fassi, P. Fassnacht, D. Fassouliotis, M. Faucci Giannelli, A. Favareto, W. J. Fawcett, L. Fayard, O. L. Fedin, W. Fedorko, S. Feigl, L. Feligioni, C. Feng, E. J. Feng, H. Feng, A. B. Fenyuk, L. Feremenga, P. Fernandez Martinez, S. Fernandez Perez, J. Ferrando, A. Ferrari, P. Ferrari, R. Ferrari, D. E. Ferreira de Lima, A. Ferrer, D. Ferrere, C. Ferretti, F. Fiedler, A. Filipčič, M. Filipuzzi, F. Filthaut, M. Fincke-Keeler, K. D. Finelli, M. C. N. Fiolhais, L. Fiorini, A. Fischer, C. Fischer, J. Fischer, W. C. Fisher, N. Flaschel, I. Fleck, P. Fleischmann, G. T. Fletcher, R. R. M. Fletcher, T. Flick, B. M. Flierl, L. R. Flores Castillo, M. J. Flowerdew, G. T. Forcolin, A. Formica, A. Forti, A. G. Foster, D. Fournier, H. Fox, S. Fracchia, P. Francavilla, M. Franchini, D. Francis, L. Franconi, M. Franklin, M. Frate, M. Fraternali, D. Freeborn, S. M. Fressard-Batraneanu, F. Friedrich, D. Froidevaux, J. A. Frost, C. Fukunaga, E. Fullana Torregrosa, T. Fusayasu, J. Fuster, C. Gabaldon, O. Gabizon, A. Gabrielli, A. Gabrielli, G. P. Gach, S. Gadatsch, G. Gagliardi, L. G. Gagnon, P. Gagnon, C. Galea, B. Galhardo, E. J. Gallas, B. J. Gallop, P. Gallus, G. Galster, K. K. Gan, S. Ganguly, J. Gao, Y. Gao, Y. S. Gao, F. M. Garay Walls, C. García, J. E. García Navarro, M. Garcia-Sciveres, R. W. Gardner, N. Garelli, V. Garonne, A. Gascon Bravo, K. Gasnikova, C. Gatti, A. Gaudiello, G. Gaudio, L. Gauthier, I. L. Gavrilenko, C. Gay, G. Gaycken, E. N. Gazis, Z. Gecse, C. N. P. Gee, Ch. Geich-Gimbel, M. Geisen, M. P. Geisler, K. Gellerstedt, C. Gemme, M. H. Genest, C. Geng, S. Gentile, C. Gentsos, S. George, D. Gerbaudo, A. Gershon, S. Ghasemi, M. Ghneimat, B. Giacobbe, S. Giagu, P. Giannetti, S. M. Gibson, M. Gignac, M. Gilchriese, T. P. S. Gillam, D. Gillberg, G. Gilles, D. M. Gingrich, N. Giokaris, M. P. Giordani, F. M. Giorgi, P. F. Giraud, P. Giromini, D. Giugni, F. Giuli, C. Giuliani, M. Giulini, B. K. Gjelsten, S. Gkaitatzis, I. Gkialas, E. L. Gkougkousis, L. K. Gladilin, C. Glasman, J. Glatzer, P. C. F. Glaysher, A. Glazov, M. Goblirsch-Kolb, J. Godlewski, S. Goldfarb, T. Golling, D. Golubkov, A. Gomes, R. Gonçalo, J. Goncalves Pinto Firmino Da Costa, G. Gonella, L. Gonella, A. Gongadze, S. González de la Hoz, S. Gonzalez-Sevilla, L. Goossens, P. A. Gorbounov, H. A. Gordon, I. Gorelov, B. Gorini, E. Gorini, A. Gorišek, A. T. Goshaw, C. Gössling, M. I. Gostkin, C. R. Goudet, D. Goujdami, A. G. Goussiou, N. Govender, E. Gozani, L. Graber, I. Grabowska-Bold, P. O. J. Gradin, P. Grafström, J. Gramling, E. Gramstad, S. Grancagnolo, V. Gratchev, P. M. Gravila, H. M. Gray, E. Graziani, Z. D. Greenwood, C. Grefe, K. Gregersen, I. M. Gregor, P. Grenier, K. Grevtsov, J. Griffiths, A. A. Grillo, K. Grimm, S. Grinstein, Ph. Gris, J.-F. Grivaz, S. Groh, E. Gross, J. Grosse-Knetter, G. C. Grossi, Z. J. Grout, L. Guan, W. Guan, J. Guenther, F. Guescini, D. Guest, O. Gueta, B. Gui, E. Guido, T. Guillemin, S. Guindon, U. Gul, C. Gumpert, J. Guo, W. Guo, Y. Guo, R. Gupta, S. Gupta, G. Gustavino, P. Gutierrez, N. G. Gutierrez Ortiz, C. Gutschow, C. Guyot, C. Gwenlan, C. B. Gwilliam, A. Haas, C. Haber, H. K. Hadavand, N. Haddad, A. Hadef, S. Hageböck, M. Hagihara, H. Hakobyan, M. Haleem, J. Haley, G. Halladjian, G. D. Hallewell, K. Hamacher, P. Hamal, K. Hamano, A. Hamilton, G. N. Hamity, P. G. Hamnett, L. Han, S. Han, K. Hanagaki, K. Hanawa, M. Hance, B. Haney, P. Hanke, R. Hanna, J. B. Hansen, J. D. Hansen, M. C. Hansen, P. H. Hansen, K. Hara, A. S. Hard, T. Harenberg, F. Hariri, S. Harkusha, R. D. Harrington, P. F. Harrison, F. Hartjes, N. M. Hartmann, M. Hasegawa, Y. Hasegawa, A. Hasib, S. Hassani, S. Haug, R. Hauser, L. Hauswald, M. Havranek, C. M. Hawkes, R. J. Hawkings, D. Hayakawa, D. Hayden, C. P. Hays, J. M. Hays, H. S. Hayward, S. J. Haywood, S. J. Head, T. Heck, V. Hedberg, L. Heelan, S. Heim, T. Heim, B. Heinemann, J. J. Heinrich, L. Heinrich, C. Heinz, J. Hejbal, L. Helary, S. Hellman, C. Helsens, J. Henderson, R. C. W. Henderson, Y. Heng, S. Henkelmann, A. M. Henriques Correia, S. Henrot-Versille, G. H. Herbert, H. Herde, V. Herget, Y. Hernández Jiménez, G. Herten, R. Hertenberger, L. Hervas, G. G. Hesketh, N. P. Hessey, J. W. Hetherly, E. Higón-Rodriguez, E. Hill, J. C. Hill, K. H. Hiller, S. J. Hillier, I. Hinchliffe, E. Hines, M. Hirose, D. Hirschbuehl, O. Hladik, X. Hoad, J. Hobbs, N. Hod, M. C. Hodgkinson, P. Hodgson, A. Hoecker, M. R. Hoeferkamp, F. Hoenig, D. Hohn, T. R. Holmes, M. Homann, S. Honda, T. Honda, T. M. Hong, B. H. Hooberman, W. H. Hopkins, Y. Horii, A. J. Horton, J-Y. Hostachy, S. Hou, A. Hoummada, J. Howarth, J. Hoya, M. Hrabovsky, I. Hristova, J. Hrivnac, T. Hryn’ova, A. Hrynevich, P. J. Hsu, S.-C. Hsu, Q. Hu, S. Hu, Y. Huang, Z. Hubacek, F. Hubaut, F. Huegging, T. B. Huffman, E. W. Hughes, G. Hughes, M. Huhtinen, P. Huo, N. Huseynov, J. Huston, J. Huth, G. Iacobucci, G. Iakovidis, I. Ibragimov, L. Iconomidou-Fayard, E. Ideal, Z. Idrissi, P. Iengo, O. Igonkina, T. Iizawa, Y. Ikegami, M. Ikeno, Y. Ilchenko, D. Iliadis, N. Ilic, G. Introzzi, P. Ioannou, M. Iodice, K. Iordanidou, V. Ippolito, N. Ishijima, M. Ishino, M. Ishitsuka, C. Issever, S. Istin, F. Ito, J. M. Iturbe Ponce, R. Iuppa, H. Iwasaki, J. M. Izen, V. Izzo, S. Jabbar, B. Jackson, P. Jackson, V. Jain, K. B. Jakobi, K. Jakobs, S. Jakobsen, T. Jakoubek, D. O. Jamin, D. K. Jana, R. Jansky, J. Janssen, M. Janus, P. A. Janus, G. Jarlskog, N. Javadov, T. Javůrek, F. Jeanneau, L. Jeanty, J. Jejelava, G.-Y. Jeng, P. Jenni, C. Jeske, S. Jézéquel, H. Ji, J. Jia, H. Jiang, Y. Jiang, Z. Jiang, S. Jiggins, J. Jimenez Pena, S. Jin, A. Jinaru, O. Jinnouchi, H. Jivan, P. Johansson, K. A. Johns, C. A. Johnson, W. J. Johnson, K. Jon-And, G. Jones, R. W. L. Jones, S. Jones, T. J. Jones, J. Jongmanns, P. M. Jorge, J. Jovicevic, X. Ju, A. Juste Rozas, M. K. Köhler, A. Kaczmarska, M. Kado, H. Kagan, M. Kagan, S. J. Kahn, T. Kaji, E. Kajomovitz, C. W. Kalderon, A. Kaluza, S. Kama, A. Kamenshchikov, N. Kanaya, S. Kaneti, L. Kanjir, V. A. Kantserov, J. Kanzaki, B. Kaplan, L. S. Kaplan, A. Kapliy, D. Kar, K. Karakostas, A. Karamaoun, N. Karastathis, M. J. Kareem, E. Karentzos, M. Karnevskiy, S. N. Karpov, Z. M. Karpova, K. Karthik, V. Kartvelishvili, A. N. Karyukhin, K. Kasahara, L. Kashif, R. D. Kass, A. Kastanas, Y. Kataoka, C. Kato, A. Katre, J. Katzy, K. Kawade, K. Kawagoe, T. Kawamoto, G. Kawamura, V. F. Kazanin, R. Keeler, R. Kehoe, J. S. Keller, J. J. Kempster, H. Keoshkerian, O. Kepka, B. P. Kerševan, S. Kersten, R. A. Keyes, M. Khader, F. Khalil-zada, A. Khanov, A. G. Kharlamov, T. Kharlamova, T. J. Khoo, V. Khovanskiy, E. Khramov, J. Khubua, S. Kido, C. R. Kilby, H. Y. Kim, S. H. Kim, Y. K. Kim, N. Kimura, O. M. Kind, B. T. King, M. King, J. Kirk, A. E. Kiryunin, T. Kishimoto, D. Kisielewska, F. Kiss, K. Kiuchi, O. Kivernyk, E. Kladiva, T. Klapdor-kleingrothaus, M. H. Klein, M. Klein, U. Klein, K. Kleinknecht, P. Klimek, A. Klimentov, R. Klingenberg, T. Klioutchnikova, E.-E. Kluge, P. Kluit, S. Kluth, J. Knapik, E. Kneringer, E. B. F. G. Knoops, A. Knue, A. Kobayashi, D. Kobayashi, T. Kobayashi, M. Kobel, M. Kocian, P. Kodys, T. Koffas, E. Koffeman, N. M. Köhler, T. Koi, H. Kolanoski, M. Kolb, I. Koletsou, A. A. Komar, Y. Komori, T. Kondo, N. Kondrashova, K. Köneke, A. C. König, T. Kono, R. Konoplich, N. Konstantinidis, R. Kopeliansky, S. Koperny, A. K. Kopp, K. Korcyl, K. Kordas, A. Korn, A. A. Korol, I. Korolkov, E. V. Korolkova, O. Kortner, S. Kortner, T. Kosek, V. V. Kostyukhin, A. Kotwal, A. Koulouris, A. Kourkoumeli-Charalampidi, C. Kourkoumelis, V. Kouskoura, A. B. Kowalewska, R. Kowalewski, T. Z. Kowalski, C. Kozakai, W. Kozanecki, A. S. Kozhin, V. A. Kramarenko, G. Kramberger, D. Krasnopevtsev, M. W. Krasny, A. Krasznahorkay, A. Kravchenko, M. Kretz, J. Kretzschmar, K. Kreutzfeldt, P. Krieger, K. Krizka, K. Kroeninger, H. Kroha, J. Kroll, J. Kroseberg, J. Krstic, U. Kruchonak, H. Krüger, N. Krumnack, M. C. Kruse, M. Kruskal, T. Kubota, H. Kucuk, S. Kuday, J. T. Kuechler, S. Kuehn, A. Kugel, F. Kuger, T. Kuhl, V. Kukhtin, R. Kukla, Y. Kulchitsky, S. Kuleshov, M. Kuna, T. Kunigo, A. Kupco, O. Kuprash, H. Kurashige, L. L. Kurchaninov, Y. A. Kurochkin, M. G. Kurth, V. Kus, E. S. Kuwertz, M. Kuze, J. Kvita, T. Kwan, D. Kyriazopoulos, A. La Rosa, J. L. La Rosa Navarro, L. La Rotonda, C. Lacasta, F. Lacava, J. Lacey, H. Lacker, D. Lacour, E. Ladygin, R. Lafaye, B. Laforge, T. Lagouri, S. Lai, S. Lammers, W. Lampl, E. Lançon, U. Landgraf, M. P. J. Landon, M. C. Lanfermann, V. S. Lang, J. C. Lange, A. J. Lankford, F. Lanni, K. Lantzsch, A. Lanza, S. Laplace, C. Lapoire, J. F. Laporte, T. Lari, F. Lasagni Manghi, M. Lassnig, P. Laurelli, W. Lavrijsen, A. T. Law, P. Laycock, T. Lazovich, M. Lazzaroni, B. Le, O. Le Dortz, E. Le Guirriec, E. P. Le Quilleuc, M. LeBlanc, T. LeCompte, F. Ledroit-Guillon, C. A. Lee, S. C. Lee, L. Lee, B. Lefebvre, G. Lefebvre, M. Lefebvre, F. Legger, C. Leggett, A. Lehan, G. Lehmann Miotto, X. Lei, W. A. Leight, A. G. Leister, M. A. L. Leite, R. Leitner, D. Lellouch, B. Lemmer, K. J. C. Leney, T. Lenz, B. Lenzi, R. Leone, S. Leone, C. Leonidopoulos, S. Leontsinis, G. Lerner, C. Leroy, A. A. J. Lesage, C. G. Lester, M. Levchenko, J. Levêque, D. Levin, L. J. Levinson, M. Levy, D. Lewis, M. Leyton, B. Li, C. Li, H. Li, L. Li, L. Li, Q. Li, S. Li, X. Li, Y. Li, Z. Liang, B. Liberti, A. Liblong, P. Lichard, K. Lie, J. Liebal, W. Liebig, A. Limosani, S. C. Lin, T. H. Lin, B. E. Lindquist, A. E. Lionti, E. Lipeles, A. Lipniacka, M. Lisovyi, T. M. Liss, A. Lister, A. M. Litke, B. Liu, D. Liu, H. Liu, H. Liu, J. Liu, J. B. Liu, K. Liu, L. Liu, M. Liu, Y. L. Liu, Y. Liu, M. Livan, A. Lleres, J. Llorente Merino, S. L. Lloyd, F. Lo Sterzo, E. M. Lobodzinska, P. Loch, F. K. Loebinger, K. M. Loew, A. Loginov, T. Lohse, K. Lohwasser, M. Lokajicek, B. A. Long, J. D. Long, R. E. Long, L. Longo, K. A. Looper, J. A. Lopez Lopez, D. Lopez Mateos, B. Lopez Paredes, I. Lopez Paz, A. Lopez Solis, J. Lorenz, N. Lorenzo Martinez, M. Losada, P. J. Lösel, X. Lou, A. Lounis, J. Love, P. A. Love, H. Lu, N. Lu, H. J. Lubatti, C. Luci, A. Lucotte, C. Luedtke, F. Luehring, W. Lukas, L. Luminari, O. Lundberg, B. Lund-Jensen, P. M. Luzi, D. Lynn, R. Lysak, E. Lytken, V. Lyubushkin, H. Ma, L. L. Ma, Y. Ma, G. Maccarrone, A. Macchiolo, C. M. Macdonald, B. Maček, J. Machado Miguens, D. Madaffari, R. Madar, H. J. Maddocks, W. F. Mader, A. Madsen, J. Maeda, S. Maeland, T. Maeno, A. Maevskiy, E. Magradze, J. Mahlstedt, C. Maiani, C. Maidantchik, A. A. Maier, T. Maier, A. Maio, S. Majewski, Y. Makida, N. Makovec, B. Malaescu, Pa. Malecki, V. P. Maleev, F. Malek, U. Mallik, D. Malon, C. Malone, S. Maltezos, S. Malyukov, J. Mamuzic, G. Mancini, L. Mandelli, I. Mandić, J. Maneira, L. Manhaes de Andrade Filho, J. Manjarres Ramos, A. Mann, A. Manousos, B. Mansoulie, J. D. Mansour, R. Mantifel, M. Mantoani, S. Manzoni, L. Mapelli, G. Marceca, L. March, G. Marchiori, M. Marcisovsky, M. Marjanovic, D. E. Marley, F. Marroquim, S. P. Marsden, Z. Marshall, S. Marti-Garcia, B. Martin, T. A. Martin, V. J. Martin, B. Martin dit Latour, M. Martinez, V. I. Martinez Outschoorn, S. Martin-Haugh, V. S. Martoiu, A. C. Martyniuk, A. Marzin, L. Masetti, T. Mashimo, R. Mashinistov, J. Masik, A. L. Maslennikov, I. Massa, L. Massa, P. Mastrandrea, A. Mastroberardino, T. Masubuchi, P. Mättig, J. Mattmann, J. Maurer, S. J. Maxfield, D. A. Maximov, R. Mazini, I. Maznas, S. M. Mazza, N. C. Mc Fadden, G. Mc Goldrick, S. P. Mc Kee, A. McCarn, R. L. McCarthy, T. G. McCarthy, L. I. McClymont, E. F. McDonald, J. A. Mcfayden, G. Mchedlidze, S. J. McMahon, R. A. McPherson, M. Medinnis, S. Meehan, S. Mehlhase, A. Mehta, K. Meier, C. Meineck, B. Meirose, D. Melini, B. R. Mellado Garcia, M. Melo, F. Meloni, S. B. Menary, L. Meng, X. T. Meng, A. Mengarelli, S. Menke, E. Meoni, S. Mergelmeyer, P. Mermod, L. Merola, C. Meroni, F. S. Merritt, A. Messina, J. Metcalfe, A. S. Mete, C. Meyer, C. Meyer, J-P. Meyer, J. Meyer, H. Meyer Zu Theenhausen, F. Miano, R. P. Middleton, S. Miglioranzi, L. Mijović, G. Mikenberg, M. Mikestikova, M. Mikuž, M. Milesi, A. Milic, D. W. Miller, C. Mills, A. Milov, D. A. Milstead, A. A. Minaenko, Y. Minami, I. A. Minashvili, A. I. Mincer, B. Mindur, M. Mineev, Y. Minegishi, Y. Ming, L. M. Mir, K. P. Mistry, T. Mitani, J. Mitrevski, V. A. Mitsou, A. Miucci, P. S. Miyagawa, A. Mizukami, J. U. Mjörnmark, M. Mlynarikova, T. Moa, K. Mochizuki, P. Mogg, S. Mohapatra, S. Molander, R. Moles-Valls, R. Monden, M. C. Mondragon, K. Mönig, J. Monk, E. Monnier, A. Montalbano, J. Montejo Berlingen, F. Monticelli, S. Monzani, R. W. Moore, N. Morange, D. Moreno, M. Moreno Llácer, P. Morettini, S. Morgenstern, D. Mori, T. Mori, M. Morii, M. Morinaga, V. Morisbak, S. Moritz, A. K. Morley, G. Mornacchi, J. D. Morris, S. S. Mortensen, L. Morvaj, P. Moschovakos, M. Mosidze, H. J. Moss, J. Moss, K. Motohashi, R. Mount, E. Mountricha, E. J. W. Moyse, S. Muanza, R. D. Mudd, F. Mueller, J. Mueller, R. S. P. Mueller, T. Mueller, D. Muenstermann, P. Mullen, G. A. Mullier, F. J. Munoz Sanchez, J. A. Murillo Quijada, W. J. Murray, H. Musheghyan, M. Muškinja, A. G. Myagkov, M. Myska, B. P. Nachman, O. Nackenhorst, K. Nagai, R. Nagai, K. Nagano, Y. Nagasaka, K. Nagata, M. Nagel, E. Nagy, A. M. Nairz, Y. Nakahama, K. Nakamura, T. Nakamura, I. Nakano, R. F. Naranjo Garcia, R. Narayan, D. I. Narrias Villar, I. Naryshkin, T. Naumann, G. Navarro, R. Nayyar, H. A. Neal, P. Yu. Nechaeva, T. J. Neep, A. Negri, M. Negrini, S. Nektarijevic, C. Nellist, A. Nelson, S. Nemecek, P. Nemethy, A. A. Nepomuceno, M. Nessi, M. S. Neubauer, M. Neumann, R. M. Neves, P. Nevski, P. R. Newman, D. H. Nguyen, T. Nguyen Manh, R. B. Nickerson, R. Nicolaidou, J. Nielsen, V. Nikolaenko, I. Nikolic-Audit, K. Nikolopoulos, J. K. Nilsen, P. Nilsson, Y. Ninomiya, A. Nisati, R. Nisius, T. Nobe, M. Nomachi, I. Nomidis, T. Nooney, S. Norberg, M. Nordberg, N. Norjoharuddeen, O. Novgorodova, S. Nowak, M. Nozaki, L. Nozka, K. Ntekas, E. Nurse, F. Nuti, F. O’grady, D. C. O’Neil, A. A. O’Rourke, V. O’Shea, F. G. Oakham, H. Oberlack, T. Obermann, J. Ocariz, A. Ochi, I. Ochoa, J. P. Ochoa-Ricoux, S. Oda, S. Odaka, H. Ogren, A. Oh, S. H. Oh, C. C. Ohm, H. Ohman, H. Oide, H. Okawa, Y. Okumura, T. Okuyama, A. Olariu, L. F. Oleiro Seabra, S. A. Olivares Pino, D. Oliveira Damazio, A. Olszewski, J. Olszowska, A. Onofre, K. Onogi, P. U. E. Onyisi, M. J. Oreglia, Y. Oren, D. Orestano, N. Orlando, R. S. Orr, B. Osculati, R. Ospanov, G. Otero y Garzon, H. Otono, M. Ouchrif, F. Ould-Saada, A. Ouraou, K. P. Oussoren, Q. Ouyang, M. Owen, R. E. Owen, V. E. Ozcan, N. Ozturk, K. Pachal, A. Pacheco Pages, L. Pacheco Rodriguez, C. Padilla Aranda, M. Pagáčová, S. Pagan Griso, M. Paganini, F. Paige, P. Pais, K. Pajchel, G. Palacino, S. Palazzo, S. Palestini, M. Palka, D. Pallin, E. St. Panagiotopoulou, C. E. Pandini, J. G. Panduro Vazquez, P. Pani, S. Panitkin, D. Pantea, L. Paolozzi, Th. D. Papadopoulou, K. Papageorgiou, A. Paramonov, D. Paredes Hernandez, A. J. Parker, M. A. Parker, K. A. Parker, F. Parodi, J. A. Parsons, U. Parzefall, V. R. Pascuzzi, E. Pasqualucci, S. Passaggio, Fr. Pastore, G. Pásztor, S. Pataraia, J. R. Pater, T. Pauly, J. Pearce, B. Pearson, L. E. Pedersen, M. Pedersen, S. Pedraza Lopez, R. Pedro, S. V. Peleganchuk, O. Penc, C. Peng, H. Peng, J. Penwell, B. S. Peralva, M. M. Perego, D. V. Perepelitsa, E. Perez Codina, L. Perini, H. Pernegger, S. Perrella, R. Peschke, V. D. Peshekhonov, K. Peters, R. F. Y. Peters, B. A. Petersen, T. C. Petersen, E. Petit, A. Petridis, C. Petridou, P. Petroff, E. Petrolo, M. Petrov, F. Petrucci, N. E. Pettersson, A. Peyaud, R. Pezoa, P. W. Phillips, G. Piacquadio, E. Pianori, A. Picazio, E. Piccaro, M. Piccinini, M. A. Pickering, R. Piegaia, J. E. Pilcher, A. D. Pilkington, A. W. J. Pin, M. Pinamonti, J. L. Pinfold, A. Pingel, S. Pires, H. Pirumov, M. Pitt, L. Plazak, M.-A. Pleier, V. Pleskot, E. Plotnikova, D. Pluth, R. Poettgen, L. Poggioli, D. Pohl, G. Polesello, A. Poley, A. Policicchio, R. Polifka, A. Polini, C. S. Pollard, V. Polychronakos, K. Pommès, L. Pontecorvo, B. G. Pope, G. A. Popeneciu, A. Poppleton, S. Pospisil, K. Potamianos, I. N. Potrap, C. J. Potter, C. T. Potter, G. Poulard, J. Poveda, V. Pozdnyakov, M. E. Pozo Astigarraga, P. Pralavorio, A. Pranko, S. Prell, D. Price, L. E. Price, M. Primavera, S. Prince, K. Prokofiev, F. Prokoshin, S. Protopopescu, J. Proudfoot, M. Przybycien, D. Puddu, M. Purohit, P. Puzo, J. Qian, G. Qin, Y. Qin, A. Quadt, W. B. Quayle, M. Queitsch-Maitland, D. Quilty, S. Raddum, V. Radeka, V. Radescu, S. K. Radhakrishnan, P. Radloff, P. Rados, F. Ragusa, G. Rahal, J. A. Raine, S. Rajagopalan, M. Rammensee, C. Rangel-Smith, M. G. Ratti, D. M. Rauch, F. Rauscher, S. Rave, T. Ravenscroft, I. Ravinovich, M. Raymond, A. L. Read, N. P. Readioff, M. Reale, D. M. Rebuzzi, A. Redelbach, G. Redlinger, R. Reece, R. G. Reed, K. Reeves, L. Rehnisch, J. Reichert, A. Reiss, C. Rembser, H. Ren, M. Rescigno, S. Resconi, E. D. Resseguie, O. L. Rezanova, P. Reznicek, R. Rezvani, R. Richter, S. Richter, E. Richter-Was, O. Ricken, M. Ridel, P. Rieck, C. J. Riegel, J. Rieger, O. Rifki, M. Rijssenbeek, A. Rimoldi, M. Rimoldi, L. Rinaldi, B. Ristić, E. Ritsch, I. Riu, F. Rizatdinova, E. Rizvi, C. Rizzi, R. T. Roberts, S. H. Robertson, A. Robichaud-Veronneau, D. Robinson, J. E. M. Robinson, A. Robson, C. Roda, Y. Rodina, A. Rodriguez Perez, D. Rodriguez Rodriguez, S. Roe, C. S. Rogan, O. Røhne, J. Roloff, A. Romaniouk, M. Romano, S. M. Romano Saez, E. Romero Adam, N. Rompotis, M. Ronzani, L. Roos, E. Ros, S. Rosati, K. Rosbach, P. Rose, N.-A. Rosien, V. Rossetti, E. Rossi, L. P. Rossi, J. H. N. Rosten, R. Rosten, M. Rotaru, I. Roth, J. Rothberg, D. Rousseau, A. Rozanov, Y. Rozen, X. Ruan, F. Rubbo, M. S. Rudolph, F. Rühr, A. Ruiz-Martinez, Z. Rurikova, N. A. Rusakovich, A. Ruschke, H. L. Russell, J. P. Rutherfoord, N. Ruthmann, Y. F. Ryabov, M. Rybar, G. Rybkin, S. Ryu, A. Ryzhov, G. F. Rzehorz, A. F. Saavedra, G. Sabato, S. Sacerdoti, H.F-W. Sadrozinski, R. Sadykov, F. Safai Tehrani, P. Saha, M. Sahinsoy, M. Saimpert, T. Saito, H. Sakamoto, Y. Sakurai, G. Salamanna, A. Salamon, J. E. Salazar Loyola, D. Salek, P. H. Sales De Bruin, D. Salihagic, A. Salnikov, J. Salt, D. Salvatore, F. Salvatore, A. Salvucci, A. Salzburger, D. Sammel, D. Sampsonidis, J. Sánchez, V. Sanchez Martinez, A. Sanchez Pineda, H. Sandaker, R. L. Sandbach, M. Sandhoff, C. Sandoval, D. P. C. Sankey, M. Sannino, A. Sansoni, C. Santoni, R. Santonico, H. Santos, I. Santoyo Castillo, K. Sapp, A. Sapronov, J. G. Saraiva, B. Sarrazin, O. Sasaki, K. Sato, E. Sauvan, G. Savage, P. Savard, N. Savic, C. Sawyer, L. Sawyer, J. Saxon, C. Sbarra, A. Sbrizzi, T. Scanlon, D. A. Scannicchio, M. Scarcella, V. Scarfone, J. Schaarschmidt, P. Schacht, B. M. Schachtner, D. Schaefer, L. Schaefer, R. Schaefer, J. Schaeffer, S. Schaepe, S. Schaetzel, U. Schäfer, A. C. Schaffer, D. Schaile, R. D. Schamberger, V. Scharf, V. A. Schegelsky, D. Scheirich, M. Schernau, C. Schiavi, S. Schier, C. Schillo, M. Schioppa, S. Schlenker, K. R. Schmidt-Sommerfeld, K. Schmieden, C. Schmitt, S. Schmitt, S. Schmitz, B. Schneider, U. Schnoor, L. Schoeffel, A. Schoening, B. D. Schoenrock, E. Schopf, M. Schott, J. F. P. Schouwenberg, J. Schovancova, S. Schramm, M. Schreyer, N. Schuh, A. Schulte, M. J. Schultens, H.-C. Schultz-Coulon, H. Schulz, M. Schumacher, B. A. Schumm, Ph. Schune, A. Schwartzman, T. A. Schwarz, H. Schweiger, Ph. Schwemling, R. Schwienhorst, J. Schwindling, T. Schwindt, G. Sciolla, F. Scuri, F. Scutti, J. Searcy, P. Seema, S. C. Seidel, A. Seiden, F. Seifert, J. M. Seixas, G. Sekhniaidze, K. Sekhon, S. J. Sekula, D. M. Seliverstov, N. Semprini-Cesari, C. Serfon, L. Serin, L. Serkin, M. Sessa, R. Seuster, H. Severini, T. Sfiligoj, F. Sforza, A. Sfyrla, E. Shabalina, N. W. Shaikh, L. Y. Shan, R. Shang, J. T. Shank, M. Shapiro, P. B. Shatalov, K. Shaw, S. M. Shaw, A. Shcherbakova, C. Y. Shehu, P. Sherwood, L. Shi, S. Shimizu, C. O. Shimmin, M. Shimojima, S. Shirabe, M. Shiyakova, A. Shmeleva, D. Shoaleh Saadi, M. J. Shochet, S. Shojaii, D. R. Shope, S. Shrestha, E. Shulga, M. A. Shupe, P. Sicho, A. M. Sickles, P. E. Sidebo, E. Sideras Haddad, O. Sidiropoulou, D. Sidorov, A. Sidoti, F. Siegert, Dj. Sijacki, J. Silva, S. B. Silverstein, V. Simak, Lj. Simic, S. Simion, E. Simioni, B. Simmons, D. Simon, M. Simon, P. Sinervo, N. B. Sinev, M. Sioli, G. Siragusa, I. Siral, S. Yu. Sivoklokov, J. Sjölin, M. B. Skinner, H. P. Skottowe, P. Skubic, M. Slater, T. Slavicek, M. Slawinska, K. Sliwa, R. Slovak, V. Smakhtin, B. H. Smart, L. Smestad, J. Smiesko, S. Yu. Smirnov, Y. Smirnov, L. N. Smirnova, O. Smirnova, J. W. Smith, M. N. K. Smith, R. W. Smith, M. Smizanska, K. Smolek, A. A. Snesarev, I. M. Snyder, S. Snyder, R. Sobie, F. Socher, A. Soffer, D. A. Soh, G. Sokhrannyi, C. A. Solans Sanchez, M. Solar, E. Yu. Soldatov, U. Soldevila, A. A. Solodkov, A. Soloshenko, O. V. Solovyanov, V. Solovyev, P. Sommer, H. Son, H. Y. Song, A. Sood, A. Sopczak, V. Sopko, V. Sorin, D. Sosa, C. L. Sotiropoulou, R. Soualah, A. M. Soukharev, D. South, B. C. Sowden, S. Spagnolo, M. Spalla, M. Spangenberg, F. Spanò, D. Sperlich, F. Spettel, R. Spighi, G. Spigo, L. A. Spiller, M. Spousta, R. D. St. Denis, A. Stabile, R. Stamen, S. Stamm, E. Stanecka, R. W. Stanek, C. Stanescu, M. Stanescu-Bellu, M. M. Stanitzki, S. Stapnes, E. A. Starchenko, G. H. Stark, J. Stark, P. Staroba, P. Starovoitov, S. Stärz, R. Staszewski, P. Steinberg, B. Stelzer, H. J. Stelzer, O. Stelzer-Chilton, H. Stenzel, G. A. Stewart, J. A. Stillings, M. C. Stockton, M. Stoebe, G. Stoicea, P. Stolte, S. Stonjek, A. R. Stradling, A. Straessner, M. E. Stramaglia, J. Strandberg, S. Strandberg, A. Strandlie, M. Strauss, P. Strizenec, R. Ströhmer, D. M. Strom, R. Stroynowski, A. Strubig, S. A. Stucci, B. Stugu, N. A. Styles, D. Su, J. Su, S. Suchek, Y. Sugaya, M. Suk, V. V. Sulin, S. Sultansoy, T. Sumida, S. Sun, X. Sun, J. E. Sundermann, K. Suruliz, C. J. E. Suster, M. R. Sutton, S. Suzuki, M. Svatos, M. Swiatlowski, S. P. Swift, I. Sykora, T. Sykora, D. Ta, K. Tackmann, J. Taenzer, A. Taffard, R. Tafirout, N. Taiblum, H. Takai, R. Takashima, T. Takeshita, Y. Takubo, M. Talby, A. A. Talyshev, J. Tanaka, M. Tanaka, R. Tanaka, S. Tanaka, R. Tanioka, B. B. Tannenwald, S. Tapia Araya, S. Tapprogge, S. Tarem, G. F. Tartarelli, P. Tas, M. Tasevsky, T. Tashiro, E. Tassi, A. Tavares Delgado, Y. Tayalati, A. C. Taylor, G. N. Taylor, P. T. E. Taylor, W. Taylor, F. A. Teischinger, P. Teixeira-Dias, K. K. Temming, D. Temple, H. Ten Kate, P. K. Teng, J. J. Teoh, F. Tepel, S. Terada, K. Terashi, J. Terron, S. Terzo, M. Testa, R. J. Teuscher, T. Theveneaux-Pelzer, J. P. Thomas, J. Thomas-Wilsker, P. D. Thompson, A. S. Thompson, L. A. Thomsen, E. Thomson, M. J. Tibbetts, R. E. Ticse Torres, V. O. Tikhomirov, Yu. A. Tikhonov, S. Timoshenko, P. Tipton, S. Tisserant, K. Todome, T. Todorov, S. Todorova-Nova, J. Tojo, S. Tokár, K. Tokushuku, E. Tolley, L. Tomlinson, M. Tomoto, L. Tompkins, K. Toms, B. Tong, P. Tornambe, E. Torrence, H. Torres, E. Torró Pastor, J. Toth, F. Touchard, D. R. Tovey, T. Trefzger, A. Tricoli, I. M. Trigger, S. Trincaz-Duvoid, M. F. Tripiana, W. Trischuk, B. Trocmé, A. Trofymov, C. Troncon, M. Trottier-McDonald, M. Trovatelli, L. Truong, M. Trzebinski, A. Trzupek, J.C-L. Tseng, P. V. Tsiareshka, G. Tsipolitis, N. Tsirintanis, S. Tsiskaridze, V. Tsiskaridze, E. G. Tskhadadze, K. M. Tsui, I. I. Tsukerman, V. Tsulaia, S. Tsuno, D. Tsybychev, Y. Tu, A. Tudorache, V. Tudorache, T. T. Tulbure, A. N. Tuna, S. A. Tupputi, S. Turchikhin, D. Turgeman, I. Turk Cakir, R. Turra, P. M. Tuts, G. Ucchielli, I. Ueda, M. Ughetto, F. Ukegawa, G. Unal, A. Undrus, G. Unel, F. C. Ungaro, Y. Unno, C. Unverdorben, J. Urban, P. Urquijo, P. Urrejola, G. Usai, J. Usui, L. Vacavant, V. Vacek, B. Vachon, C. Valderanis, E. Valdes Santurio, N. Valencic, S. Valentinetti, A. Valero, L. Valery, S. Valkar, J. A. Valls Ferrer, W. Van Den Wollenberg, P. C. Van Der Deijl, H. van der Graaf, N. van Eldik, P. van Gemmeren, J. Van Nieuwkoop, I. van Vulpen, M. C. van Woerden, M. Vanadia, W. Vandelli, R. Vanguri, A. Vaniachine, P. Vankov, G. Vardanyan, R. Vari, E. W. Varnes, T. Varol, D. Varouchas, A. Vartapetian, K. E. Varvell, J. G. Vasquez, G. A. Vasquez, F. Vazeille, T. Vazquez Schroeder, J. Veatch, V. Veeraraghavan, L. M. Veloce, F. Veloso, S. Veneziano, A. Ventura, M. Venturi, N. Venturi, A. Venturini, V. Vercesi, M. Verducci, W. Verkerke, J. C. Vermeulen, A. Vest, M. C. Vetterli, O. Viazlo, I. Vichou, T. Vickey, O. E. Vickey Boeriu, G. H. A. Viehhauser, S. Viel, L. Vigani, M. Villa, M. Villaplana Perez, E. Vilucchi, M. G. Vincter, V. B. Vinogradov, C. Vittori, I. Vivarelli, S. Vlachos, M. Vlasak, M. Vogel, P. Vokac, G. Volpi, M. Volpi, H. von der Schmitt, E. von Toerne, V. Vorobel, K. Vorobev, M. Vos, R. Voss, J. H. Vossebeld, N. Vranjes, M. Vranjes Milosavljevic, V. Vrba, M. Vreeswijk, R. Vuillermet, I. Vukotic, P. Wagner, W. Wagner, H. Wahlberg, S. Wahrmund, J. Wakabayashi, J. Walder, R. Walker, W. Walkowiak, V. Wallangen, C. Wang, C. Wang, F. Wang, H. Wang, H. Wang, J. Wang, J. Wang, K. Wang, R. Wang, S. M. Wang, T. Wang, W. Wang, C. Wanotayaroj, A. Warburton, C. P. Ward, D. R. Wardrope, A. Washbrook, P. M. Watkins, A. T. Watson, M. F. Watson, G. Watts, S. Watts, B. M. Waugh, S. Webb, M. S. Weber, S. W. Weber, S. A. Weber, J. S. Webster, A. R. Weidberg, B. Weinert, J. Weingarten, C. Weiser, H. Weits, P. S. Wells, T. Wenaus, T. Wengler, S. Wenig, N. Wermes, M. D. Werner, P. Werner, M. Wessels, J. Wetter, K. Whalen, N. L. Whallon, A. M. Wharton, A. White, M. J. White, R. White, D. Whiteson, F. J. Wickens, W. Wiedenmann, M. Wielers, C. Wiglesworth, L. A. M. Wiik-Fuchs, A. Wildauer, F. Wilk, H. G. Wilkens, H. H. Williams, S. Williams, C. Willis, S. Willocq, J. A. Wilson, I. Wingerter-Seez, F. Winklmeier, O. J. Winston, B. T. Winter, M. Wittgen, M. Wobisch, T. M. H. Wolf, R. Wolff, M. W. Wolter, H. Wolters, S. D. Worm, B. K. Wosiek, J. Wotschack, M. J. Woudstra, K. W. Wozniak, M. Wu, M. Wu, S. L. Wu, X. Wu, Y. Wu, T. R. Wyatt, B. M. Wynne, S. Xella, Z. Xi, D. Xu, L. Xu, B. Yabsley, S. Yacoob, D. Yamaguchi, Y. Yamaguchi, A. Yamamoto, S. Yamamoto, T. Yamanaka, K. Yamauchi, Y. Yamazaki, Z. Yan, H. Yang, H. Yang, Y. Yang, Z. Yang, W-M. Yao, Y. C. Yap, Y. Yasu, E. Yatsenko, K. H. Yau Wong, J. Ye, S. Ye, I. Yeletskikh, E. Yildirim, K. Yorita, R. Yoshida, K. Yoshihara, C. Young, C. J. S. Young, S. Youssef, D. R. Yu, J. Yu, J. M. Yu, J. Yu, L. Yuan, S. P. Y. Yuen, I. Yusuff, B. Zabinski, R. Zaidan, A. M. Zaitsev, N. Zakharchuk, J. Zalieckas, A. Zaman, S. Zambito, L. Zanello, D. Zanzi, C. Zeitnitz, M. Zeman, A. Zemla, J. C. Zeng, Q. Zeng, O. Zenin, T. Ženiš, D. Zerwas, D. Zhang, F. Zhang, G. Zhang, H. Zhang, J. Zhang, L. Zhang, L. Zhang, M. Zhang, R. Zhang, R. Zhang, X. Zhang, Z. Zhang, X. Zhao, Y. Zhao, Z. Zhao, A. Zhemchugov, J. Zhong, B. Zhou, C. Zhou, L. Zhou, L. Zhou, M. Zhou, M. Zhou, N. Zhou, C. G. Zhu, H. Zhu, J. Zhu, Y. Zhu, X. Zhuang, K. Zhukov, A. Zibell, D. Zieminska, N. I. Zimine, C. Zimmermann, S. Zimmermann, Z. Zinonos, M. Zinser, M. Ziolkowski, L. Živković, G. Zobernig, A. Zoccoli, M. zur Nedden, L. Zwalinski, ATLAS Collaboration

**Affiliations:** 1 Department of Physics, University of Adelaide, Adelaide, Australia; 2 Physics Department, SUNY Albany, Albany, NY USA; 3 Department of Physics, University of Alberta, Edmonton, AB Canada; 4 Department of Physics, Ankara University, Ankara, Turkey; 5 Istanbul Aydin University, Istanbul, Turkey; 6 Division of Physics, TOBB University of Economics and Technology, Ankara, Turkey; 7 LAPP, CNRS/IN2P3 and Université Savoie Mont Blanc, Annecy-le-Vieux, France; 8 High Energy Physics Division, Argonne National Laboratory, Argonne, IL USA; 9 Department of Physics, University of Arizona, Tucson, AZ USA; 10 Department of Physics, The University of Texas at Arlington, Arlington, TX USA; 11 Physics Department, National and Kapodistrian University of Athens, Athens, Greece; 12 Physics Department, National Technical University of Athens, Zografou, Greece; 13 Department of Physics, The University of Texas at Austin, Austin, TX USA; 14 Institute of Physics, Azerbaijan Academy of Sciences, Baku, Azerbaijan; 15 Institut de Física d’Altes Energies (IFAE), The Barcelona Institute of Science and Technology, Barcelona, Spain; 16 Institute of Physics, University of Belgrade, Belgrade, Serbia; 17 Department for Physics and Technology, University of Bergen, Bergen, Norway; 18 Physics Division, Lawrence Berkeley National Laboratory and University of California, Berkeley, CA USA; 19 Department of Physics, Humboldt University, Berlin, Germany; 20 Albert Einstein Center for Fundamental Physics and Laboratory for High Energy Physics, University of Bern, Bern, Switzerland; 21 School of Physics and Astronomy, University of Birmingham, Birmingham, UK; 22 Department of Physics, Bogazici University, Istanbul, Turkey; 23 Department of Physics Engineering, Gaziantep University, Gaziantep, Turkey; 24 Faculty of Engineering and Natural Sciences, Istanbul Bilgi University, Istanbul, Turkey; 25 Faculty of Engineering and Natural Sciences, Bahcesehir University, Istanbul, Turkey; 26 Centro de Investigaciones, Universidad Antonio Narino, Bogotá, Colombia; 27 INFN Sezione di Bologna, Bologna, Italy; 28 Dipartimento di Fisica e Astronomia, Università di Bologna, Bologna, Italy; 29 Physikalisches Institut, University of Bonn, Bonn, Germany; 30 Department of Physics, Boston University, Boston, MA USA; 31 Department of Physics, Brandeis University, Waltham, MA USA; 32 Universidade Federal do Rio De Janeiro COPPE/EE/IF, Rio de Janeiro, Brazil; 33 Electrical Circuits Department, Federal University of Juiz de Fora (UFJF), Juiz de Fora, Brazil; 34 Federal University of Sao Joao del Rei (UFSJ), Sao Joao del Rei, Brazil; 35 Instituto de Fisica, Universidade de Sao Paulo, Sao Paulo, Brazil; 36 Physics Department, Brookhaven National Laboratory, Upton, NY USA; 37 Transilvania University of Brasov, Brasov, Romania; 38 National Institute of Physics and Nuclear Engineering, Bucharest, Romania; 39 Physics Department, National Institute for Research and Development of Isotopic and Molecular Technologies, Cluj Napoca, Romania; 40 University Politehnica Bucharest, Bucharest, Romania; 41 West University in Timisoara, Timisoara, Romania; 42 Departamento de Física, Universidad de Buenos Aires, Buenos Aires, Argentina; 43 Cavendish Laboratory, University of Cambridge, Cambridge, UK; 44 Department of Physics, Carleton University, Ottawa, ON Canada; 45 CERN, Geneva, Switzerland; 46 Enrico Fermi Institute, University of Chicago, Chicago, IL USA; 47 Departamento de Física, Pontificia Universidad Católica de Chile, Santiago, Chile; 48 Departamento de Física, Universidad Técnica Federico Santa María, Valparaiso, Chile; 49 Institute of High Energy Physics, Chinese Academy of Sciences, Beijing, China; 50 Department of Physics, Nanjing University, Nanjing, Jiangsu China; 51 Physics Department, Tsinghua University, Beijing, 100084 China; 52 Department of Modern Physics, University of Science and Technology of China, Hefei, Anhui China; 53 School of Physics, Shandong University, Jinan, Shandong China; 54 Department of Physics and Astronomy, Shanghai Key Laboratory for Particle Physics and Cosmology, Shanghai Jiao Tong University, Shanghai (also affiliated with PKU-CHEP), Shanghai, China; 55 Laboratoire de Physique Corpusculaire, Université Clermont Auvergne, Université Blaise Pascal, CNRS/IN2P3, Clermont-Ferrand, France; 56 Nevis Laboratory, Columbia University, Irvington, NY USA; 57 Niels Bohr Institute, University of Copenhagen, Copenhagen, Denmark; 58 INFN Gruppo Collegato di Cosenza, Laboratori Nazionali di Frascati, Frascati, Italy; 59 Dipartimento di Fisica, Università della Calabria, Rende, Italy; 60 Faculty of Physics and Applied Computer Science, AGH University of Science and Technology, Kraków, Poland; 61 Marian Smoluchowski Institute of Physics, Jagiellonian University, Kraków, Poland; 62 Institute of Nuclear Physics, Polish Academy of Sciences, Kraków, Poland; 63 Physics Department, Southern Methodist University, Dallas, TX USA; 64 Physics Department, University of Texas at Dallas, Richardson, TX USA; 65 DESY, Hamburg, Zeuthen, Germany; 66 Lehrstuhl für Experimentelle Physik IV, Technische Universität Dortmund, Dortmund, Germany; 67 Institut für Kern- und Teilchenphysik, Technische Universität Dresden, Dresden, Germany; 68 Department of Physics, Duke University, Durham, NC USA; 69 SUPA-School of Physics and Astronomy, University of Edinburgh, Edinburgh, UK; 70 INFN Laboratori Nazionali di Frascati, Frascati, Italy; 71 Fakultät für Mathematik und Physik, Albert-Ludwigs-Universität, Freiburg, Germany; 72 Departement de Physique Nucleaire et Corpusculaire, Université de Genève, Geneva, Switzerland; 73 INFN Sezione di Genova, Genoa, Italy; 74 Dipartimento di Fisica, Università di Genova, Genoa, Italy; 75 E. Andronikashvili Institute of Physics, Iv. Javakhishvili Tbilisi State University, Tbilisi, Georgia; 76 High Energy Physics Institute, Tbilisi State University, Tbilisi, Georgia; 77 II Physikalisches Institut, Justus-Liebig-Universität Giessen, Giessen, Germany; 78 SUPA-School of Physics and Astronomy, University of Glasgow, Glasgow, UK; 79 II Physikalisches Institut, Georg-August-Universität, Göttingen, Germany; 80 Laboratoire de Physique Subatomique et de Cosmologie, Université Grenoble-Alpes, CNRS/IN2P3, Grenoble, France; 81 Laboratory for Particle Physics and Cosmology, Harvard University, Cambridge, MA USA; 82 Kirchhoff-Institut für Physik, Ruprecht-Karls-Universität Heidelberg, Heidelberg, Germany; 83 Physikalisches Institut, Ruprecht-Karls-Universität Heidelberg, Heidelberg, Germany; 84 ZITI Institut für technische Informatik, Ruprecht-Karls-Universität Heidelberg, Mannheim, Germany; 85 Faculty of Applied Information Science, Hiroshima Institute of Technology, Hiroshima, Japan; 86 Department of Physics, The Chinese University of Hong Kong, Shatin, N.T. Hong Kong; 87 Department of Physics, The University of Hong Kong, Hong Kong, China; 88 Department of Physics and Institute for Advanced Study, The Hong Kong University of Science and Technology, Clear Water Bay, Kowloon, Hong Kong, China; 89 Department of Physics, National Tsing Hua University, Hsinchu City, Taiwan; 90 Department of Physics, Indiana University, Bloomington, IN USA; 91 Institut für Astro- und Teilchenphysik, Leopold-Franzens-Universität, Innsbruck, Austria; 92 University of Iowa, Iowa City, IA USA; 93 Department of Physics and Astronomy, Iowa State University, Ames, IA USA; 94 Joint Institute for Nuclear Research, JINR Dubna, Dubna, Russia; 95 KEK, High Energy Accelerator Research Organization, Tsukuba, Japan; 96 Graduate School of Science, Kobe University, Kobe, Japan; 97 Faculty of Science, Kyoto University, Kyoto, Japan; 98 Kyoto University of Education, Kyoto, Japan; 99 Department of Physics, Kyushu University, Fukuoka, Japan; 100 Instituto de Física La Plata, Universidad Nacional de La Plata and CONICET, La Plata, Argentina; 101 Physics Department, Lancaster University, Lancaster, UK; 102 INFN Sezione di Lecce, Lecce, Italy; 103 Dipartimento di Matematica e Fisica, Università del Salento, Lecce, Italy; 104 Oliver Lodge Laboratory, University of Liverpool, Liverpool, UK; 105 Department of Experimental Particle Physics, Jožef Stefan Institute and Department of Physics, University of Ljubljana, Ljubljana, Slovenia; 106 School of Physics and Astronomy, Queen Mary University of London, London, UK; 107 Department of Physics, Royal Holloway University of London, Surrey, UK; 108 Department of Physics and Astronomy, University College London, London, UK; 109 Louisiana Tech University, Ruston, LA USA; 110 Laboratoire de Physique Nucléaire et de Hautes Energies, UPMC and Université Paris-Diderot and CNRS/IN2P3, Paris, France; 111 Fysiska institutionen, Lunds universitet, Lund, Sweden; 112 Departamento de Fisica Teorica C-15, Universidad Autonoma de Madrid, Madrid, Spain; 113 Institut für Physik, Universität Mainz, Mainz, Germany; 114 School of Physics and Astronomy, University of Manchester, Manchester, UK; 115 CPPM, Aix-Marseille Université and CNRS/IN2P3, Marseille, France; 116 Department of Physics, University of Massachusetts, Amherst, MA USA; 117 Department of Physics, McGill University, Montreal, QC Canada; 118 School of Physics, University of Melbourne, Victoria, Australia; 119 Department of Physics, The University of Michigan, Ann Arbor, MI USA; 120 Department of Physics and Astronomy, Michigan State University, East Lansing, MI USA; 121 INFN Sezione di Milano, Milan, Italy; 122 Dipartimento di Fisica, Università di Milano, Milan, Italy; 123 B.I. Stepanov Institute of Physics, National Academy of Sciences of Belarus, Minsk, Republic of Belarus; 124 Research Institute for Nuclear Problems of Byelorussian State University, Minsk, Republic of Belarus; 125 Group of Particle Physics, University of Montreal, Montreal, QC Canada; 126 P.N. Lebedev Physical Institute of the Russian Academy of Sciences, Moscow, Russia; 127 Institute for Theoretical and Experimental Physics (ITEP), Moscow, Russia; 128 National Research Nuclear University MEPhI, Moscow, Russia; 129 D.V. Skobeltsyn Institute of Nuclear Physics, M.V. Lomonosov Moscow State University, Moscow, Russia; 130 Fakultät für Physik, Ludwig-Maximilians-Universität München, München, Germany; 131 Max-Planck-Institut für Physik (Werner-Heisenberg-Institut), Münich, Germany; 132 Nagasaki Institute of Applied Science, Nagasaki, Japan; 133 Graduate School of Science and Kobayashi-Maskawa Institute, Nagoya University, Nagoya, Japan; 134 INFN Sezione di Napoli, Naples, Italy; 135 Dipartimento di Fisica, Università di Napoli, Naples, Italy; 136 Department of Physics and Astronomy, University of New Mexico, Albuquerque, NM USA; 137 Institute for Mathematics, Astrophysics and Particle Physics, Radboud University Nijmegen/Nikhef, Nijmegen, The Netherlands; 138 Nikhef National Institute for Subatomic Physics and University of Amsterdam, Amsterdam, The Netherlands; 139 Department of Physics, Northern Illinois University, DeKalb, IL USA; 140 Budker Institute of Nuclear Physics, SB RAS, Novosibirsk, Russia; 141 Department of Physics, New York University, New York, NY USA; 142 Ohio State University, Columbus, OH USA; 143 Faculty of Science, Okayama University, Okayama, Japan; 144 Homer L. Dodge Department of Physics and Astronomy, University of Oklahoma, Norman, OK USA; 145 Department of Physics, Oklahoma State University, Stillwater, OK USA; 146 Palacký University, RCPTM, Olomouc, Czech Republic; 147 Center for High Energy Physics, University of Oregon, Eugene, OR USA; 148 LAL, Univ. Paris-Sud, CNRS/IN2P3, Université Paris-Saclay, Orsay, France; 149 Graduate School of Science, Osaka University, Osaka, Japan; 150 Department of Physics, University of Oslo, Oslo, Norway; 151 Department of Physics, Oxford University, Oxford, UK; 152 INFN Sezione di Pavia, Pavia, Italy; 153 Dipartimento di Fisica, Università di Pavia, Pavia, Italy; 154 Department of Physics, University of Pennsylvania, Philadelphia, PA USA; 155 National Research Centre “Kurchatov Institute” B.P. Konstantinov Petersburg Nuclear Physics Institute, St. Petersburg, Russia; 156 INFN Sezione di Pisa, Pisa, Italy; 157 Dipartimento di Fisica E. Fermi, Università di Pisa, Pisa, Italy; 158 Department of Physics and Astronomy, University of Pittsburgh, Pittsburgh, PA USA; 159 Laboratório de Instrumentação e Física Experimental de Partículas-LIP, Lisbon, Portugal; 160 Faculdade de Ciências, Universidade de Lisboa, Lisbon, Portugal; 161 Department of Physics, University of Coimbra, Coimbra, Portugal; 162 Centro de Física Nuclear da Universidade de Lisboa, Lisbon, Portugal; 163 Departamento de Fisica, Universidade do Minho, Braga, Portugal; 164 Departamento de Fisica Teorica y del Cosmos and CAFPE, Universidad de Granada, Granada, Spain; 165 Dep Fisica and CEFITEC of Faculdade de Ciencias e Tecnologia, Universidade Nova de Lisboa, Caparica, Lisbon, Portugal; 166 Institute of Physics, Academy of Sciences of the Czech Republic, Prague, Czech Republic; 167 Czech Technical University in Prague, Prague, Czech Republic; 168 Faculty of Mathematics and Physics, Charles University in Prague, Prague, Czech Republic; 169 State Research Center Institute for High Energy Physics (Protvino), NRC KI, Protvino, Russia; 170 Particle Physics Department, Rutherford Appleton Laboratory, Didcot, UK; 171 INFN Sezione di Roma, Rome, Italy; 172 Dipartimento di Fisica, Sapienza Università di Roma, Rome, Italy; 173 INFN Sezione di Roma Tor Vergata, Rome, Italy; 174 Dipartimento di Fisica, Università di Roma Tor Vergata, Rome, Italy; 175 INFN Sezione di Roma Tre, Rome, Italy; 176 Dipartimento di Matematica e Fisica, Università Roma Tre, Rome, Italy; 177 Faculté des Sciences Ain Chock, Réseau Universitaire de Physique des Hautes Energies-Université Hassan II, Casablanca, Morocco; 178 Centre National de l’Energie des Sciences Techniques Nucleaires, Rabat, Morocco; 179 Faculté des Sciences Semlalia, Université Cadi Ayyad, LPHEA-Marrakech, Marrakech, Morocco; 180 Faculté des Sciences, Université Mohamed Premier and LPTPM, Oujda, Morocco; 181 Faculté des Sciences, Université Mohammed V, Rabat, Morocco; 182 DSM/IRFU (Institut de Recherches sur les Lois Fondamentales de l’Univers), CEA Saclay (Commissariat à l’Energie Atomique et aux Energies Alternatives), Gif-sur-Yvette, France; 183 Santa Cruz Institute for Particle Physics, University of California Santa Cruz, Santa Cruz, CA USA; 184 Department of Physics, University of Washington, Seattle, WA USA; 185 Department of Physics and Astronomy, University of Sheffield, Sheffield, UK; 186 Department of Physics, Shinshu University, Nagano, Japan; 187 Fachbereich Physik, Universität Siegen, Siegen, Germany; 188 Department of Physics, Simon Fraser University, Burnaby, BC Canada; 189 SLAC National Accelerator Laboratory, Stanford, CA USA; 190 Faculty of Mathematics, Physics and Informatics, Comenius University, Bratislava, Slovak Republic; 191 Department of Subnuclear Physics, Institute of Experimental Physics of the Slovak Academy of Sciences, Kosice, Slovak Republic; 192 Department of Physics, University of Cape Town, Cape Town, South Africa; 193 Department of Physics, University of Johannesburg, Johannesburg, South Africa; 194 School of Physics, University of the Witwatersrand, Johannesburg, South Africa; 195 Department of Physics, Stockholm University, Stockholm, Sweden; 196 The Oskar Klein Centre, Stockholm, Sweden; 197 Physics Department, Royal Institute of Technology, Stockholm, Sweden; 198 Departments of Physics and Astronomy and Chemistry, Stony Brook University, Stony Brook, NY USA; 199 Department of Physics and Astronomy, University of Sussex, Brighton, UK; 200 School of Physics, University of Sydney, Sydney, Australia; 201 Institute of Physics, Academia Sinica, Taipei, Taiwan; 202 Department of Physics, Technion: Israel Institute of Technology, Haifa, Israel; 203 Raymond and Beverly Sackler School of Physics and Astronomy, Tel Aviv University, Tel Aviv, Israel; 204 Department of Physics, Aristotle University of Thessaloniki, Thessaloníki, Greece; 205 International Center for Elementary Particle Physics and Department of Physics, The University of Tokyo, Tokyo, Japan; 206 Graduate School of Science and Technology, Tokyo Metropolitan University, Tokyo, Japan; 207 Department of Physics, Tokyo Institute of Technology, Tokyo, Japan; 208 Tomsk State University, Tomsk, Russia; 209 Department of Physics, University of Toronto, Toronto, ON Canada; 210 INFN-TIFPA, Trento, Italy; 211 University of Trento, Trento, Italy; 212 TRIUMF, Vancouver, BC Canada; 213 Department of Physics and Astronomy, York University, Toronto, ON Canada; 214 Faculty of Pure and Applied Sciences, and Center for Integrated Research in Fundamental Science and Engineering, University of Tsukuba, Tsukuba, Japan; 215 Department of Physics and Astronomy, Tufts University, Medford, MA USA; 216 Department of Physics and Astronomy, University of California Irvine, Irvine, CA USA; 217 INFN Gruppo Collegato di Udine, Sezione di Trieste, Udine, Italy; 218 ICTP, Trieste, Italy; 219 Dipartimento di Chimica, Fisica e Ambiente, Università di Udine, Udine, Italy; 220 Department of Physics and Astronomy, University of Uppsala, Uppsala, Sweden; 221 Department of Physics, University of Illinois, Urbana, IL USA; 222 Instituto de Fisica Corpuscular (IFIC) and Departamento de Fisica Atomica, Molecular y Nuclear and Departamento de Ingeniería Electrónica and Instituto de Microelectrónica de Barcelona (IMB-CNM), University of Valencia and CSIC, Valencia, Spain; 223 Department of Physics, University of British Columbia, Vancouver, BC Canada; 224 Department of Physics and Astronomy, University of Victoria, Victoria, BC Canada; 225 Department of Physics, University of Warwick, Coventry, UK; 226 Waseda University, Tokyo, Japan; 227 Department of Particle Physics, The Weizmann Institute of Science, Rehovot, Israel; 228 Department of Physics, University of Wisconsin, Madison, WI USA; 229 Fakultät für Physik und Astronomie, Julius-Maximilians-Universität, Würzburg, Germany; 230 Fakultät für Mathematik und Naturwissenschaften, Fachgruppe Physik, Bergische Universität Wuppertal, Wuppertal, Germany; 231 Department of Physics, Yale University, New Haven, CT USA; 232 Yerevan Physics Institute, Yerevan, Armenia; 233 Centre de Calcul de l’Institut National de Physique Nucléaire et de Physique des Particules (IN2P3), Villeurbanne, France; 234 CERN, 1211 Geneva 23, Switzerland

## Abstract

Detailed measurements of *t*-channel single top-quark production are presented. They use 20.2 fb$^{-1}$ of data collected by the ATLAS experiment in proton–proton collisions at a centre-of-mass energy of 8 TeV at the LHC. Total, fiducial and differential cross-sections are measured for both top-quark and top-antiquark production. The fiducial cross-section is measured with a precision of 5.8% (top quark) and 7.8% (top antiquark), respectively. The total cross-sections are measured to be $\sigma _{\text {tot}} (tq) = 56.7^{+4.3}_{-3.8}\;\mathrm{pb}$ for top-quark production and $\sigma _{\text {tot}} (\bar{t} q) = 32.9^{+3.0}_{-2.7}\;\mathrm{pb}$ for top-antiquark production, in agreement with the Standard Model prediction. In addition, the ratio of top-quark to top-antiquark production cross-sections is determined to be $R_t=1.72 \pm 0.09$. The differential cross-sections as a function of the transverse momentum and rapidity of both the top quark and the top antiquark are measured at both the parton and particle levels. The transverse momentum and rapidity differential cross-sections of the accompanying jet from the *t*-channel scattering are measured at particle level. All measurements are compared to various Monte Carlo predictions as well as to fixed-order QCD calculations where available.

## Introduction

**Fig. 1:**
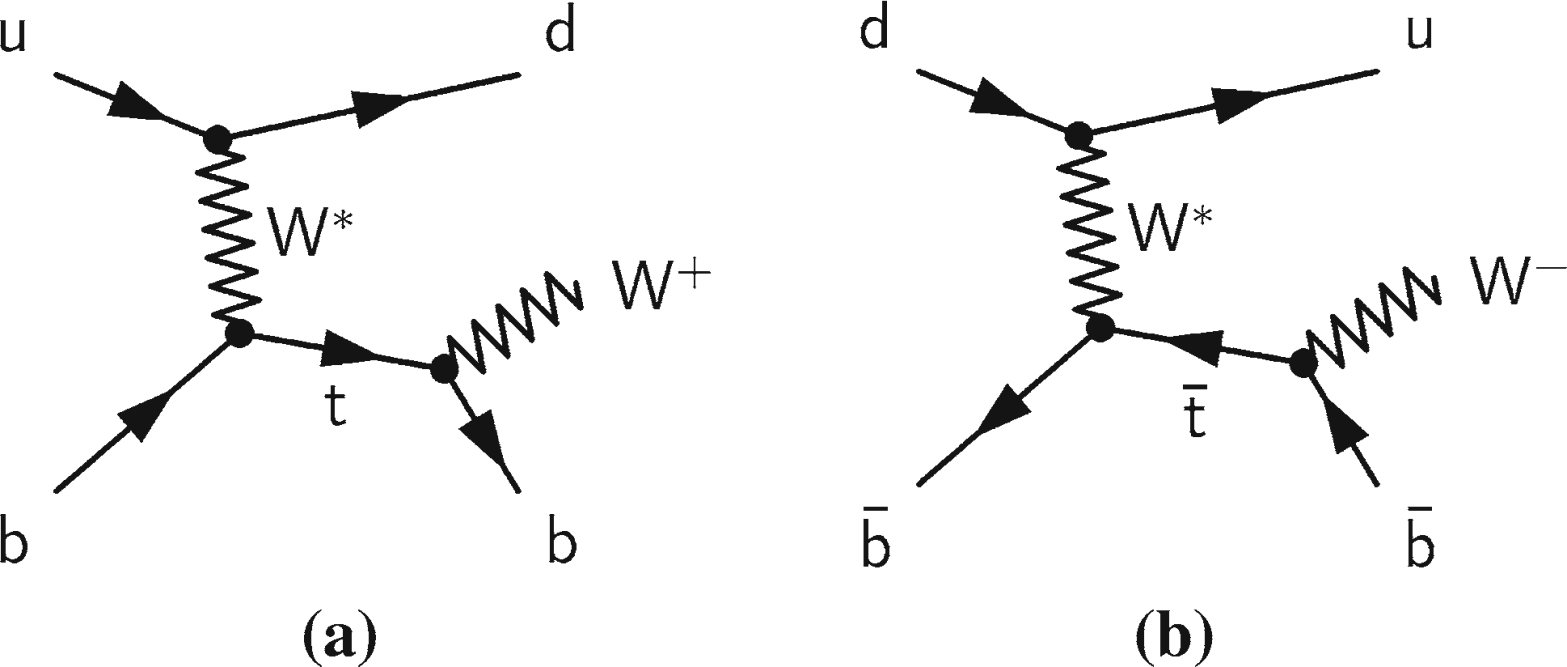
Representative leading-order Feynman diagrams for **a** single top-quark production and **b** single top-antiquark production via the *t*-channel exchange of a virtual $W^*$ boson, including the decay of the top quark and top antiquark, respectively

Top quarks are produced singly in proton–proton ($pp$) collisions via electroweak charged-current interactions. In leading-order (LO) perturbation theory, single top-quark production is described by three subprocesses that are distinguished by the virtuality of the exchanged *W* boson. The dominant process is the *t*-channel exchange depicted in Fig. 1, where a light quark from one of the colliding protons interacts with a *b*-quark from another proton by exchanging a virtual *W* boson ($W^*$). Since the valence *u*-quark density of the proton is about twice as high as the valence *d*-quark density, the production cross-section of single top quarks, $\sigma (tq)$, is expected to be about twice as high as the cross-section of top-antiquark production, $\sigma (\bar{t}q)$. At LO, subdominant single-top-quark processes are the associated production of a *W* boson and a top quark (*Wt*) and the *s*-channel production of $t\bar{b}$. The *t*-channel and *s*-channel processes do not interfere even at next-to-leading order (NLO) in perturbation theory and are thus well defined with that precision.

This paper presents measurements of $\sigma (tq)$ and $\sigma (\bar{t}q)$ in $pp$ collisions at a centre-of-mass energy of $\sqrt{s} = {8}\,{\mathrm{TeV}}$ at the Large Hadron Collider (LHC). The analysis is based on the full ATLAS dataset collected in 2012, corresponding to an integrated luminosity of 20.2 fb$^{-1}$. Separate measurements of *tq* and $\bar{t}q$ production provide sensitivity to the parton distribution functions (PDFs) of the *u*-quark and the *d*-quark, exploiting the different initial states of the two processes as shown in Fig. 1. In addition, the cross-section ratio $R_t \equiv \sigma (tq)/\sigma (\bar{t}q)$ is measured, which has smaller systematic uncertainties than the individual cross-sections, because of partial cancellations of common uncertainties. Investigating $R_t$ also provides a way of searching for new-physics contributions in single top-quark (top-antiquark) production [1] and of elucidating the nature of physics beyond the Standard Model (SM) if it were to be observed [2].

In general, measurements of single top-quark production provide insights into the properties of the *Wtb* interaction. The cross-sections are proportional to the square of the coupling at the *Wtb* production vertex. In the SM, the coupling is given by the Cabibbo–Kobayashi–Maskawa (CKM) matrix element $V_{tb}$ [3, 4] multiplied by the universal electroweak coupling constant. All measurements presented in this paper are based on the assumption that the production and the decay of top quarks via *Wts* and *Wtd* vertices are suppressed due to the fact that the CKM matrix elements $V_{ts}$ and $V_{td}$ are much smaller than $V_{tb}$. Potential new-physics contributions to the *Wtb* vertex are parameterised by an additional left-handed form factor $f_{\text {LV}}$  [5], assumed to be real. In this approach the Lorentz structure is assumed to be the same as in the SM, that is vector–axial-vector ($\mathrm {V}-\mathrm {A}$). The inclusive cross-section $\sigma (tq +\bar{t}q)$ is determined as the sum of $\sigma (tq)$ and $\sigma (\bar{t}q)$ and used to determine $f_{\text {LV}} \cdot |V_{tb}|$. Alternatively, the measurement of $\sigma (tq +\bar{t}q)$ can be used to constrain the *b*-quark PDF. The measurement of $\sigma (tq +\bar{t}q)$ is also sensitive to various models of new-physics phenomena [6], such as extra heavy quarks, gauge bosons, or scalar bosons. Studies of differential cross-sections allow the modelling of the process to be probed in more detail and provide a more sensitive search for effects of new physics.

Single top-quark production in the *t*-channel was first established in $p\bar{p}$ collisions at $\sqrt{s} = {1.96}\,\mathrm{TeV}$ at the Tevatron [7, 8]. Measurements of *t*-channel single top-quark production at the LHC at $\sqrt{s} = {7} \mathrm{TeV}$ were performed by the ATLAS Collaboration [9, 10] and the CMS Collaboration [11, 12]. At $\sqrt{s} = {8} \mathrm{TeV}$ the CMS Collaboration measured the *t*-channel cross-sections and the cross-section ratio, $R_t$ [13].

The total inclusive cross-sections of top-quark and top-antiquark production in the *t*-channel in *pp* collisions at $\sqrt{s} = {8}\,\mathrm{TeV}$ are predicted to be

1a $$\begin{aligned} \sigma (tq)&= {54.9 ^{+2.3}_{-1.9}}\mathrm{pb}, \end{aligned}$$

1b $$\begin{aligned} \sigma (\bar{t}q)&= {29.7 ^{+1.7}_{-1.5}}\mathrm{pb}, \end{aligned}$$

1c $$\begin{aligned} \sigma (tq+\bar{t}q)&= {84.6 ^{+3.9}_{-3.4}}\mathrm{pb}, \end{aligned}$$

at NLO accuracy in QCD. The cross-sections are calculated with the HatHor  v2.1 [14] tool, which is based on work documented in Ref. [15]. The top-quark mass $$m_t $$ is assumed to be 172.5 GeV, the same value which is used for the samples of simulated events in this analysis. The central values quoted in Eqs. (1a)–(1c) are determined following the PDF4LHC prescription [16], which defines the central value as the midpoint of the uncertainty envelope of three PDF sets: MSTW2008  [17, 18], CT10  NLO [19] and NNPDF 3.0  [20]. The uncertainty due to the PDFs and their $$\alpha _{\text {S}} $$ dependence is given by half of the width of the envelope defined by these PDFs and is added in quadrature to the scale uncertainty to obtain the total uncertainties quoted in Eqs. (1a)–(1c). The sensitivity of $$\sigma (tq)$$ and $$\sigma (\bar{t}q)$$ to the PDFs has recently gained attention in the literature [21]. The scale uncertainties in the predictions are determined following a prescription referred to as independent restricted scale variations, in which the renormalisation scale ($$\mu _{\text {r}}$$) and the factorisation scale ($$\mu _{\text {f}}$$) are varied independently, considering the default choices $$\mu _{\text {r}}^{\text {def}}$$and $$\mu _{\text {f}}^{\text {def}}$$, half the default scales and two times the default scales. The combinations ($$0.5 \mu _{\text {r}}^{\text {def}}$$, $$2.0 \mu _{\text {f}}^{\text {def}}$$) and ($$2.0 \mu _{\text {r}}^{\text {def}}$$, $$0.5 \mu _{\text {f}}^{\text {def}}$$) are excluded, thus “restricted variations”. The maximum deviations in the predicted cross-sections for the six probed variations define the uncertainty.

Predictions of $\sigma (tq)$ and $\sigma (\bar{t}q)$ have recently been calculated at next-to-next-to-leading order (NNLO) [22]. The calculation uses $m_t ={173.2}\,\mathrm{GeV}$ and $\mu _{\text {r}}=\mu _{\text {f}}=m_t$, and results in a cross-section which is 1.5% lower than the NLO value calculated with the same settings. Only a limited number of scale variations are presented in Ref. [22]; however, they do indicate a reduction in the scale uncertainties compared to the NLO result. Since the NLO computation implemented in HatHor allows a complete treatment of the scale and PDF uncertainties, which is not currently available for the NNLO calculation, the NLO computation is used when extracting $f_{\text {LV}} \cdot |V_{tb}|$ and for comparing the $R_t$ measurement to different PDF sets. The NLO results have been augmented by including the resummation of soft-gluon terms at next-to-next-to-leading logarithmic (NNLL) accuracy [23–25], leading to fixed-order predictions at the so-called NLO + NNLL level.

Cross-sections are measured in two ways: over the full kinematic range and within a fiducial phase space, defined to be as close as possible to the experimental measurement range. The definition of the fiducial phase space is based on stable particles output by Monte Carlo (MC) generators, with which reconstructed objects, such as primary leptons, jets and missing transverse momentum, are defined. The advantage of the fiducial cross-section measurements is a substantial reduction of the size of the applied acceptance corrections, leading to reduced systematic uncertainties.

Differential cross-sections are measured as a function of the transverse momentum of the top (anti)quark, $p_{\text {T}} (t)$, and as a function of the absolute value of its rapidity, $|y(t)|$. The measured cross-sections are unfolded to both parton level and particle level. Parton-level measurements can be directly compared to theory predictions that use stable top quarks. Particle-level measurements make use of a top-quark proxy which is constructed with the objects used in the fiducial cross-section measurements. At particle level, it is also possible to measure differential cross-sections as a function of the $p_{\text {T}}$ and rapidity of the jet formed by the scattered light quark in the *t*-channel exchange of a *W* boson.

Events are selected targeting the $t \rightarrow \ell \nu b$ decay mode of the top quark where the lepton can be either an electron or a muon originating from a *W*-boson decay.^1^ The experimental signature of candidate events is thus given by one charged lepton (electron or muon), large values of the magnitude of the missing transverse momentum, $E_{\text {T}}^{\text {miss}}$, and two hadronic jets with high transverse momentum. Exactly one of the two hadronic jets is required to be identified as a jet containing *b*-hadrons (*b*-jet). The other hadronic jet is referred to as the *untagged* jet and is assumed to be the accompanying jet in the *t*-channel exchange.

Several other processes feature the same signature as single-top-quark events; the main backgrounds being $W$ + jets production and top-quark–top-antiquark ($t\bar{t}$) pair production. Since a typical signature-based event selection yields only a relatively low signal purity, a dedicated analysis strategy is developed to separate signal and background events. Several observables discriminating between signal and background events are combined by an artificial neural network (NN) into one discriminant, $O_{\text {NN}}$, with improved signal-to-background separation. The cross-section measurements are based on a maximum-likelihood fit to the $O_{\text {NN}}$ distribution. In addition, a cut on $O_{\text {NN}}$ is applied to obtain a sample of events enriched in *t*-channel single-top-quark events. These events are used to extract differential cross-sections as a function of both the top-quark and untagged-jet variables.

This paper is organised as follows. The ATLAS detector is introduced in Sect. 2; details of both the data set and simulated event samples are given in Sect. 3. The objects used to select events are introduced in Sect. 4, while Sect. 5 discusses the event selection criteria. In Sect. 6 the background estimation is described. The measured cross-sections are defined in detail in Sect. 7 before turning to the separation of signal from background using a neural network in Sect. 8. The sources of systematic uncertainty considered in the analyses are covered in Sect. 9. The fiducial and inclusive cross-section measurements are the subject of Sect. 10, including the measurement of $R_{t}$ and $f_{\text {LV}} \cdot |V_{tb}|$. This is followed by the differential cross-section measurements in Sect. 11, which also explains the method used to unfold the cross-sections. Finally, the conclusion is given in Sect. 12.

## ATLAS detector

The ATLAS experiment [26] at the LHC is a multi-purpose particle detector with a forward-backward symmetric cylindrical geometry and a near $4\pi$ coverage in solid angle.^2^ It consists of an inner tracking detector (ID) surrounded by a thin superconducting solenoid providing a 2 T axial magnetic field, electromagnetic and hadron calorimeters, and a muon spectrometer. The ID covers the pseudorapidity range $|\eta | < 2.5$. It consists of silicon pixel, silicon microstrip, and transition-radiation tracking detectors. Lead/liquid-argon (LAr) sampling calorimeters provide electromagnetic (EM) energy measurements with high granularity. A hadron (steel/scintillator-tile) calorimeter covers the central pseudorapidity range ($|\eta | < 1.7$). The endcap ($1.5< |\eta | < 3.2$) and forward regions ($3.1< |\eta | < 4.9$) are instrumented with LAr calorimeters for both the EM and hadronic energy measurements. The muon spectrometer (MS) surrounds the calorimeters and is based on three large air-core toroid superconducting magnets with eight coils each. Its bending power ranges from 2.0 to 7.5 Tm. It includes a system of precision tracking chambers and fast detectors for triggering. A three-level trigger system is used to select events. The first-level trigger is implemented in hardware and uses a subset of the detector information to reduce the accepted rate to at most 75 kHz. This is followed by two software-based trigger levels that together reduce the accepted event rate to 400 Hz on average, depending on the data-taking conditions during 2012.

## Data sample and simulation

This analysis is performed using $pp$ collision data recorded at a centre-of-mass energy of $\sqrt{s}={8}\,\mathrm{TeV}$ with the ATLAS detector at the LHC. Only the data-taking periods in which all the subdetectors were operational are considered. The data sets used in this analysis are defined by high-$p_{\text {T}}$ single-electron or single-muon triggers [27, 28], resulting in a data sample with an integrated luminosity of $L_{\text {int}} = {20.2}$ fb$^{-1}$ [29].

In the first-level trigger, electron-channel events are triggered by a cluster of energy depositions in the electromagnetic calorimeter. In the software-based triggers, a cluster of energy depositions in the calorimeter needs to be matched to a track and the trigger electron candidate is required to have transverse energy $E_{\text {T}} > {60}\,\mathrm{GeV}$, or $E_{\text {T}} > {24}\,\mathrm{GeV}$ with additional isolation requirements.

The single-muon trigger is based on muon candidates reconstructed in the muon spectrometer. Muon-channel events are accepted by the trigger if they have either a muon with transverse momentum $p_{\text {T}} > {36}\,\mathrm{GeV}$ or an isolated muon with $p_{\text {T}} > {24}\,\mathrm{GeV}$.

Simulated signal and background samples were generated with an MC technique. Detector and trigger simulations are performed within the dedicated ATLAS simulation software infrastructure utilizing the GEANT4 framework [30, 31]. The same offline reconstruction methods used with data events are applied to the samples of simulated events. Multiple inelastic *pp* collisions (referred to as pile-up) are simulated with Pythia 8  [32], and are overlaid on each MC event. Weights are assigned to the simulated events such that the distribution of the number of pile-up interactions in the simulation matches the corresponding distribution in the data, which has an average of 21 [29].

Single-top-quark events from *t*-channel production are generated using the Powheg-Box  (r2556) [33] generator. This generator uses the four-flavour scheme (4FS) for the NLO matrix element (ME) calculations, since the 4FS leads to a more precise description of the event kinematics compared to the five-flavour scheme (5FS). Events are generated with the fixed four-flavour PDF set CT10f4  [19] and the renormalisation and factorisation scales are set to the recommendation given in Ref. [33]. Top quarks are decayed at LO using MadSpin  [34], preserving all spin correlations. The parton shower, hadronisation and the underlying event are modelled using the Pythia 6  (v6.428) [35] generator and a set of tuned parameters called the Perugia2012 tune (P2012) [36].

For the generation of single top-quarks in the *Wt* and the *s*-channel the Powheg-Box (r2819) generator [37, 38] with the CT10 PDF set is used. Samples of $t\bar{t}$ events are generated with the Powheg-Box (r3026) [39] and the CT10 PDF set. In the event generation of $t\bar{t}$, the $h_{\text {damp}}$ parameter, which controls the $p_{\text {T}}$ spectrum of the first additional emission beyond the Born configuration, is set to the mass of the top quark. The main effect of this is to regulate the high-$p_{\text {T}}$ emission against which the $t\bar{t}$ system recoils. The parton shower, hadronisation and the underlying event are added using Pythia 6 and the P2011C set of tuned parameters [36].

All top-quark processes are generated assuming a top-quark mass of 172.5 GeV. The decay of the top quark is assumed to be exclusively $t \rightarrow Wb$.

For studies of systematic uncertainties in all processes involving top quarks, either alternative generators or parameter variations in the Powheg-Box + Pythia 6 setup are used. To study the hadronisation modelling, the Powheg-Box generator interfaced to Herwig (v6.5.20)  [40] is used. The underlying event is simulated using the Jimmy  (v4.31) [41] model with the ATLAS AUET2 [42] set of tuned parameters. For studies of the NLO matching method, MadGraph5_aMC@NLO  (v2.2.2) [43] interfaced to Herwig is used. Samples are generated using the CT10f4 PDF set in the ME calculations and the renormalisation and factorisation scales are set to be the same as those implemented in Powheg-Box. Again, the top quarks produced in the ME are decayed using MadSpin, preserving all spin correlations. Variations of the amount of additional radiation are studied by generating samples using Powheg-Box + Pythia 6 after changing the hard-scatter scales and the scales in the parton shower simultaneously. In these samples, a variation of the factorisation and renormalisation scales by a factor of 2.0 is combined with the Perugia2012radLo parameters and a variation of both parameters by a factor of 0.5 is combined with the Perugia2012radHi parameters [36]. In the case of the up-variation, the $h_{\text {damp}}$ parameter is also changed and set to two times the top-quark mass [44].

Vector-boson production in association with jets, $V$ + jets, is simulated using the multi-leg LO generator Sherpa (v1.4.1)  [45] with its own parameter tune and the CT10 PDF set. Sherpa is used not only to generate the hard process, but also for the parton shower and the modelling of the underlying event. Samples of $W$ + jets and $Z$ + jets events with up to four additional partons are generated. The CKKW method [46] is used to remove overlap between partonic configurations generated by the matrix element and by parton shower evolution. Double counting between the inclusive $V+n$ parton samples and samples with associated heavy-quark pair production is avoided consistently by applying the CKKW method also to heavy quarks [46]. In Sherpa, massive *c*- and *b*-quarks are used in the ME as well as in the shower.

Diboson events, denoted *VV*, are also simulated using the Sherpa (v1.4.1) generator. The matrix elements contain all diagrams with four electroweak vertices. They are calculated for zero additional partons at NLO and up to three additional partons at LO using the same methodology as for $V$ + jets production. Only decay modes where one boson decays leptonically and the other boson decays hadronically are considered. The CT10 PDF set is used in conjunction with a dedicated set of parton-shower parameters developed by the Sherpa authors.

## Object definitions

Electron candidates are selected from energy deposits (clusters) in the LAr EM calorimeter associated with a well-measured track fulfilling strict quality requirements [47, 48]. Electron candidates are required to satisfy $p_{\text {T}} > {25} \mathrm{GeV}$ and $|\eta _{\text {clus}}| < 2.47$, where $\eta _{\text {clus}}$ denotes the pseudorapidity of the cluster. Clusters in the calorimeter barrel–endcap transition region, corresponding to $1.37<|\eta _{\text {clus}}|<1.52$, are ignored. High-$p_{\text {T}}$  electrons associated with the *W*-boson decay can be mimicked by hadronic jets reconstructed as electrons, electrons from the decay of heavy quarks, and photon conversions. Since electrons from the *W*-boson decay are typically isolated from hadronic jet activity, backgrounds are suppressed by isolation criteria, which require minimal calorimeter activity and only allow low-$p_{\text {T}}$ tracks in an $\eta$–$\phi$ cone around the electron candidate. Isolation criteria are optimised to achieve a uniform selection efficiency of 90% as a function of $\eta _{\text {clus}}$ and transverse energy, $E_{\text {T}}$. The direction of the electron candidate is taken as that of the associated track. Electron candidates are isolated by imposing thresholds on the scalar sum of the transverse momenta of calorimeter energy deposits within a surrounding cone of size $\Delta R = 0.2$. In addition, the scalar sum of all track transverse momenta within a cone of size $\Delta R = 0.3$ around the electron direction is required to be below a $p_{\text {T}}$-dependent threshold in the range between 0.9 and 2.5 GeV. The track belonging to the electron candidate is excluded from the sum.

Muon candidates are reconstructed by matching track segments or complete tracks in the MS with tracks found in the ID [49]. The candidates are required to have $p_{\text {T}} > {25}\,\mathrm{GeV}$ and to be in the pseudorapidity region $|\eta |<2.5$. Isolation criteria are applied to reduce background events in which a high-$p_{\text {T}}$ muon is produced in the decay of a heavy-flavour quark. An isolation variable is defined as the scalar sum of the transverse momenta of all tracks with $p_{\text {T}}$ above 1 GeV, excluding the one matched to the muon, within a cone of size $\Delta R_{\text {iso}} = {10}\,\mathrm{GeV}/p_{\text {T}} (\mu )$. The definition of $\Delta R_{\text {iso}}$ is inspired by the one used in Ref. [50]. Muon candidates are accepted if they have an isolation to $p_{\text {T}} (\mu )$ ratio of less than 0.05. Events are rejected if the selected electron and the muon candidate share the same ID track.

Jets are reconstructed using the anti-$k_{t}$ algorithm [51] with a radius parameter of $R=0.4$, using topological clusters [52] as inputs to the jet finding. The clusters are calibrated with a local cluster weighting method [52]. The jet energy is further corrected for the effect of multiple *pp* interactions, both in data and in simulated events. Calibrated jets [53] using a transverse momentum- and $\eta$-dependent simulation-based calibration scheme, with in situ corrections based on data, are required to have $p_{\text {T}} > {30}\,\mathrm{GeV}$ and $|\eta |<4.5$. The minimum jet $p_{\text {T}}$ is raised to 35 GeV within the transition region from the endcap to the forward calorimeter, corresponding to $2.7<|\eta |<3.5$.

If any jet is within $\Delta R = 0.2$ of an electron, the closest jet is removed, since in these cases the jet and the electron are very likely to correspond to the same object. Remaining electron candidates overlapping with jets within a distance $\Delta R=0.4$ are subsequently rejected.

To reject jets from pile-up events, a so-called jet-vertex-fraction criterion [54] is applied for jets with $p_{\text {T}} < {50}\,\mathrm{GeV}$ and $|\eta | <2.4$: at least 50% of the scalar sum of the $p_{\text {T}}$ of tracks within a jet is required to be from tracks compatible with the primary vertex^3^ associated with the hard-scattering collision.

Since $W+c$ production is a major background, a *b*-tagging algorithm optimised to improve the rejection of *c*-quark jets is used. A neural-network-based algorithm is employed, which combines three different algorithms exploiting the properties of a *b*-hadron decay in a jet [55]. The resulting NN discriminant ranges from zero to one and is required to be larger than 0.8349 for a jet to be considered *b*-tagged. This requirement corresponds to a *b*-tagging efficiency of 50% and a *c*-quark jet and light-parton jet mistag acceptance of 3.9 and 0.07%, respectively. These efficiencies are determined in simulated $t\bar{t}$ events.

The missing transverse momentum (with magnitude $E_{\text {T}}^{\text {miss}}$) is calculated based on the vector sum of energy deposits in the calorimeter projected onto the transverse plane [56]. All cluster energies are corrected using the local cluster weighting method. Clusters associated with a high-$p_{\text {T}}$ jet or electron are further calibrated using their respective energy corrections. In addition, the $p_{\text {T}}$ of muons with $p_{\text {T}} > {5} \mathrm{GeV}$ is included in the calculation of $E_{\text {T}}^{\text {miss}}$. The muon energy deposited in the calorimeter is taken into account to avoid double counting.

## Event selection

The event selection requires exactly one charged lepton ($\ell$), *e* or $\mu$, exactly two jets, and $E_{\text {T}}^{\text {miss}} > {30}\,\mathrm{GeV}$. Exactly one of the jets must be *b*-tagged. The selected lepton must be within $\Delta R=0.15$ of the lepton selected by the trigger. Candidate events are selected if they contain at least one good primary vertex candidate with at least five associated tracks, each of which has $p_{\text {T}} >{400}\,\mathrm{MeV}$. Events containing misreconstructed jets are rejected. Misreconstructed jets are jets with $p_{\text {T}} > {20}\,\mathrm{GeV}$ failing to satisfy quality criteria defined in Ref. [57].

Multijet events produced in hard QCD processes may be selected, even though there is no primary lepton from a weak-boson decay. This may happen if a jet is misidentified as an isolated lepton, leading to a so-called fake lepton, or if the event has a non-prompt lepton from a hadron decay which appears to be isolated. The misidentification of jets as leptons is difficult to model in the detector simulation, which is why two specific requirements are included in the event selection to reduce the multijet background without significantly reducing the signal efficiency. The first such requirement uses the transverse mass of the lepton–$E_{\text {T}}^{\text {miss}}$ system,

2 $$\begin{aligned} m_{\text {T}}\left( \ell E_{\text {T}}^{\text {miss}} \right) = \sqrt{2 p_\mathrm {T}(\ell ) \cdot E_{\text {T}}^{\text {miss}} \left[ 1-\cos \left( \Delta \phi \left( \ell , E_{\text {T}}^{\text {miss}} \right) \right) \right] }, \end{aligned}$$

and requires it to be larger than 50 GeV. Further reduction of the multijet background is achieved by placing an additional requirement on events with a charged lepton that is back-to-back with the highest-$p_{\text {T}}$ (leading) jet. This is realised by the following requirement between the lepton $p_{\text {T}} (\ell )$ and $\Delta \phi \left( j_1, \ell \right)$:

3 $$\begin{aligned} p_{\text {T}} \left( \ell \right) > \max \left( {25} \,\mathrm{GeV}, {40} \,\mathrm{GeV} \cdot \left( 1 - \frac{\pi - |\Delta \phi \left( j_1, \ell \right) |}{\pi -1} \right) \right) , \end{aligned}$$

where $j_1$ denotes the leading jet.

Events with an additional lepton are vetoed to suppress $Z$ + jets and $t\bar{t}$ dilepton backgrounds. Only leptons with opposite charge to the primary lepton are considered for this purpose. These additional leptons are identified with less stringent quality criteria than the primary lepton. Additional leptons are not required to be isolated and must have $p_{\text {T}} > {10} \,\mathrm{GeV}$. The pseudorapidity region in which additional electrons are identified includes $|\eta (e)|<4.9$, and for additional muons $|\eta (\mu )|<2.5$. Beyond the acceptance of the ID, forward electrons are identified within the pseudorapidity range of $2.5<|\eta |<4.9$ based on calorimeter measurements only [47].

Two separate vetoes are applied, depending on the flavour of the additional lepton with respect to the primary lepton. If the additional lepton has the same flavour as the primary lepton and the invariant mass of the lepton pair is between 80 and 100  GeV, the event is rejected. If the additional lepton has a different flavour than the primary lepton, the event is rejected unless the additional lepton is within $\Delta R=0.4$ to the selected *b*-jet.

A requirement of $m(\ell b) < {160}\,\mathrm{GeV}$, where $m(\ell b)$ is the invariant mass of the lepton and the *b*-tagged jet, is imposed, in order to exclude the off-shell region of top-quark decay beyond the kinematic limit of $m(\ell b)^2=m_t ^2-m_W^2$. The off-shell region is not modelled well by the currently available MC generators since off-shell effects are not included in the underlying matrix-element calculation.

Selected events are divided into two different signal regions (SRs) according to the sign of the lepton charge. These two regions are denoted $\ell ^+$ SR and $\ell ^-$ SR.

In addition, two validation regions (VRs) are defined to be orthogonal to the SRs in the same kinematic phase space to validate the modelling of the main backgrounds, $W$ + jets and $t\bar{t}$. Events in the $W$ + jets VR pass the same requirements as events in the SR except for the *b*-tagging. Exactly one *b*-tagged jet is required, which is identified with a less stringent *b*-tagging criterion than used to define the SR. The NN-*b*-tagging discriminant must be in the interval (0.4051, 0.8349), thereby excluding the SR beyond the higher threshold. The $t\bar{t}$ VR is defined by requiring both jets to pass the same *b*-tagging requirement that is used for the SR.

## Background estimation

For all background processes, except the multijet background, the normalisations are initially estimated by using MC simulation scaled to the theoretical cross-section predictions. The associated production of an on-shell *W* boson and a top quark (*Wt*) has a predicted production cross-section of 22.3 pb [58], calculated at NLO + NNLL accuracy. The uncertainty in this cross-section is 7.6%. Predictions of the *s*-channel production are calculated at NLO using the same methodology as for the *t*-channel production based on Ref. [59] and yield a predicted cross-section of 5.2 pb with a total uncertainty of 4.2%.

The predicted $t\bar{t}$ cross-section is 253 pb. It is calculated with Top++ (v2.0) [60–65] at NNLO in QCD, including the resummation of NNLL soft-gluon terms. The uncertainties due to the PDFs and $\alpha _{\text {S}}$ are calculated using the PDF4LHC prescription [16] with the MSTW2008 68% CL NNLO, CT10 NNLO and NNPDF 3.0 PDF sets and are added in quadrature to the scale uncertainty, leading to a total uncertainty in the cross-section of 6%.

The cross-sections for inclusive *W*- and *Z*-boson production are predicted with NNLO accuracy using the FEWZ program [66, 67] to be 37.0 nb and 3.83 nb, respectively. The uncertainty is 4% and comprises the PDF and scale uncertainties.

*VV* events are normalised to the NLO cross-section of 26.9 pb provided by MCFM  [68]. The uncertainty in the inclusive cross-section for these processes is 5%.

The normalisation of the multijet background is obtained from a fit to the observed $E_{\text {T}}^{\text {miss}}$  distribution, performed independently in the signal and in the validation regions. In order to select a pure sample of multijet background events, different methods are adopted for the electron and muon channels. The “jet-lepton” model is used in the electron channel while the “anti-muon” model is used in the muon channel [69]. In case of the “jet-lepton” model, a dedicated selection is imposed on MC simulated dijet events, in order to enrich events with jets that are likely to resemble a lepton in the detector. The jet candidates are treated as a lepton henceforth. The “anti-muon” model imposes a dedicated selection on data to enrich events that contain fake muons.

To determine the normalisation of the multijet background, a binned maximum-likelihood fit is performed on the $E_{\text {T}}^{\text {miss}}$  distribution using the observed data, after applying all selection criteria except for the cut on $E_{\text {T}}^{\text {miss}}$. Fits are performed separately in two $\eta$ regions for electrons: in the barrel ($|\eta | < 1.37$) and endcap ($|\eta | > 1.52$) region of the electromagnetic calorimeter, i.e. the transition region is excluded. For muons, the complete $\eta$ region is used. For the purpose of this fit, the contributions from $W$ + jets, the contributions from $t\bar{t}$ and single top-quark production, and the contributions from $Z$ + jets and *VV* production, are combined into one template. The normalisation of $Z$ + jets and *VV* backgrounds is fixed during the fit, as their contribution is small.

**Fig. 2:**
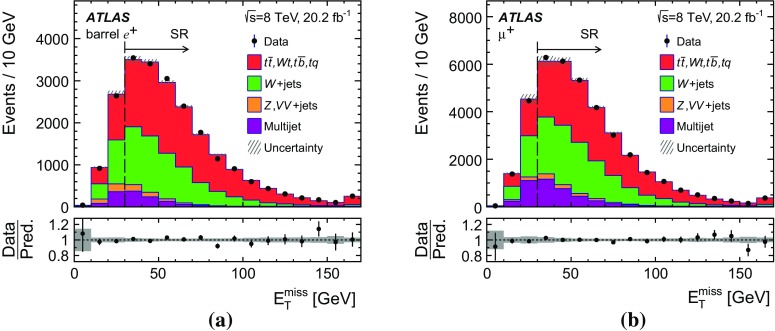
Observed distributions of the missing transverse momentum, $E_{\text {T}}^{\text {miss}}$, in the signal region (SR), including events with $E_{\text {T}}^{\text {miss}} < {30} \mathrm{GeV}$, for **a** events in the $e^{+}$ channel with an electron in the barrel region and for **b** events in the $\mu ^{+}$ channel, compared to the model obtained from simulated events. The normalisation is obtained from the binned maximum-likelihood fit to the full $E_{\text {T}}^{\text {miss}}$ distributions, and applied to the SR. The hatched uncertainty band represents the MC statistical uncertainty and the normalisation of the multijet background. The ratio of observed (Data) to predicted (Pred.) *number* of events in each bin is shown in the lower panel. Events beyond the *x*-axis range are included in the last bin

The $E_{\text {T}}^{\text {miss}}$  distributions, after rescaling the different backgrounds and the multijets template to their respective fit results, are shown in Fig. 2 for both the $e^{+}$ channel and $\mu ^{+}$ channel. The estimated event rates obtained from the binned maximum-likelihood fit for the combined contributions of $W$ + jets, $t\bar{t}$ and single top-quark production are not used in the later analysis and are only applied to scale the respective backgrounds in order to check the modelling of the kinematic distributions. For the later NN training, as well as for the final statistical analysis, the normalisation for all but the multijets background is taken solely from MC simulations scaled to their respective cross-section predictions. Based on comparisons of the rates using an alternative method, namely the matrix method [69], a systematic uncertainty of  15% is assigned to the estimated multijet yields.

Table 1 summarises the event yields in the signal region for each of the background processes considered, together with the event yields for the signal process. The quoted uncertainties are statistical uncertainties and the uncertainty in the number of multijet events. The yields are calculated using the acceptance from MC samples normalised to their respective theoretical cross-sections.

**Table 1:** Predicted and observed event yields for the signal region (SR). The multijet background prediction is obtained from a binned maximum-likelihood fit to the $E_{\text {T}}^{\text {miss}}$ distribution. All the other predictions are derived using theoretical cross-sections, given for the backgrounds in Sect. 6 and for the signal in Sect. 1. The quoted uncertainties are in the predicted cross-sections or in the number of multijet events, in case of the multijet process

Process	$\ell ^{+}$ SR	$\ell ^{-}$ SR
*tq*	11400 ± 470	17 ± 1
$\bar{t}q$	10 ± 1	6290 ± 350
$t\bar{t},Wt,t\bar{b}/\bar{t}b$	18,400 ± 1100	18,000 ± 1100
$W^{+}$ + jets	18700 ± 3700	47 ± 10
$W^{-}$ + jets	25± 5	14,000 ± 2800
$Z,VV+\mathrm {jets}$	1290 ± 260	1190 ± 240
Multijet	4520 ± 710	4520 ± 660
Total expected	54,300 ±4000	44,100 ± 3100
Data	55,800	44,687

## Measurement definitions

The paragraphs below describe the concepts and definitions on which the cross-section measurements are based.

### Fiducial and total cross-sections

Measuring a production cross-section with respect to a fiducial volume ($\sigma _{\text {fid}}$) has the benefit of reducing systematic uncertainties related to MC generators, since the extrapolation to the full phase space is avoided. In the usual case of a total cross-section measurement the measured cross-section is given by

4 $$\begin{aligned} \sigma _{\text {tot}} = \frac{\hat{\nu }}{\epsilon \cdot L_\text {int}} \quad \text {with} \quad \epsilon = \frac{N_\text {sel}}{N_\text {total}}, \end{aligned}$$

where $\hat{\nu }$ is the measured expectation value of the number of signal events and $\epsilon$ is the event selection efficiency, defined as the ratio of $N_\text {sel}$, the number of events after applying all selection cuts on a sample of simulated signal events, and $N_\text {total}$, the total number of events in that sample before any cut.

When defining a fiducial phase space, which is typically chosen to be close to the phase space of the selected data set, the fiducial acceptance is given by

5 $$\begin{aligned} A_\text {fid} = \frac{N_\text {fid}}{N_\text {total}}, \end{aligned}$$

with $N_\text {fid}$ being the number of generated events after applying the definition of the fiducial volume. The fiducial cross-section can be defined with respect to the fiducial phase space as

6 $$\begin{aligned} \sigma _{\text {fid}} = \frac{N_\text {fid}}{N_\text {sel}}\cdot \frac{\hat{\nu }}{L_\text {int}}\,. \end{aligned}$$

From Eq. (6) it is apparent that systematic effects which alter $N_\text {fid}$ and $N_\text {sel}$ by the same factor do not lead to an uncertainty in $\sigma _\text {fid}$ since the changes cancel. Using $\sigma _{\text {fid}}$ and $A_\text {fid}$, Eq. (4) can be written as

7 $$\begin{aligned} \sigma _{\text {tot}} = \frac{1}{A_\text {fid}}\cdot \sigma _{\text {fid}}, \end{aligned}$$

corresponding to the extrapolation of the fiducial cross-section to the full phase space.

### Particle-level objects

The definition of a fiducial phase space requires the implementation of the event selection at generator level. The corresponding particle-level objects are constructed from stable particles of the MC event record with a lifetime larger than 0.3E−10 s, using the following criteria.

Particle-level leptons are defined as electrons, muons or neutrinos that originate from a *W*-boson decay, including those emerging from a subsequent $\tau$-lepton decay. However, since certain MC generators do not include *W* bosons in the MC record, an implicit *W*-boson match is employed to achieve general applicability. This implicit requirement excludes leptons from hadronic decays, either directly or via a $\tau$ decay. The remaining leptons are assumed to come from a *W*-boson decay. In *t*-channel single-top-quark events, exactly one such electron or muon and the corresponding neutrino are present. The selected charged-lepton four-momentum is calculated including photons within a cone of size $\Delta R = 0.1$.

Particle-level jets are reconstructed using the anti-$k_{t}$ algorithm with a radius parameter of $R=0.4$. All stable particles are used to reconstruct the jets, except for the selected electron or muon and the photons associated with them. Particle-level jets are identified as *b*-jets, if the jet is within $|\eta | < 2.5$ and a *b*-hadron is associated with a ghost-matching technique as described in Ref. [70]. Events are rejected, if a selected particle-level lepton is identified within a cone of size $\Delta R = 0.4$ around a selected particle-level jet.

The particle-level event selection is designed to be close to the one used at reconstruction level. Exactly one particle-level electron or muon with $p_{\text {T}} > {25}\, \mathrm{GeV}$ and $|\eta | < 2.5$ is required. There must be two particle-level jets with $p_{\text {T}} > {30}\, \mathrm{GeV}$ and $|\eta | < 4.5$; exactly one of these jets must be a *b*-jet. The invariant mass of the lepton–*b*-jet system must fulfil $m(\ell b) < {160}\, \mathrm{GeV}$.

### Pseudo top quarks

Differential cross-sections characterise the top-quark kinematics. To facilitate the comparison between measurements and predictions, the top-quark objects have to closely correspond in both cases. While parton-level definitions of the top-quark are affected by ambiguities at NLO accuracy in calculations and incur related uncertainties, top-quark definitions based on stable particles in MC generators form a solid foundation. On the other hand, some calculations are only available at parton level. Following this logic, a top-quark proxy called a pseudo top quark is defined [71], based on the particle-level objects given in Sect. 7.2. Variables calculated using the pseudo top quark are denoted by $\hat{t}$, while the untagged jet is written as $\hat{j}$.

The reconstruction of the pseudo top quark starts from its decay products: the *W* boson and the *b*-tagged jet. The *W* boson is reconstructed from the charged lepton and the neutrino at particle level. The *z* component of the neutrino momentum, $p_{z}(\nu )$, is calculated using the *W*-boson mass as a constraint. If the resulting quadratic equation has two real solutions, the one with smallest absolute value of $|p_{z}(\nu )|$ is chosen. In case of complex solutions, which can occur due to the low $E_{\text {T}}^{\text {miss}}$ resolution, a kinematic fit is performed that rescales the neutrino $p_x$ and $p_y$ such that the imaginary part vanishes and at the same time the transverse components of the neutrino momentum are kept as close as possible to the $E_{\text {T}}^{\text {miss}}$. There are two jets in the events considered and exactly one of the jets is required to be *b*-tagged. The pseudo top quark is then formed by adding the four-momenta of the *W* boson and the *b*-tagged jet.

## Separation of signal from background

A neural network (NN) [72] is employed to separate signal from background events, by combining several kinematic variables into an optimised NN discriminant ($O_{\text {NN}}$). The reconstruction of top-quark-related kinematic variables, the ranking of input variables according to their discriminating power, and the training process of the NN follow closely the procedures used in previous ATLAS publications about *t*-channel single top-quark production [9, 10].

The input variables used for the NN are determined by a study in which the expected uncertainties in the cross-section measurements are computed for different sets of variables. The procedure starts from an initial set of 17 variables used in previous analyses [9, 10]. These variables are ranked based on the algorithm described in Ref. [9]. One variable after the other is removed from the network according to the ranking, starting with the lowest-ranked one, followed by the next-lowest-ranked one, and so forth. In each iteration step the full analysis is performed and the expected uncertainty of the measurement is determined. As a result of the study, it is found that the reduction from the set of six highest-ranking variables to a set of five highest-ranking variables leads to a significant increase in the uncertainty in the cross-sections. Finally, the seven highest-ranking input variables are chosen, in order to avoid sudden changes in the uncertainty due to statistical fluctuations. The input variables to the NN and their definitions are given in Table 2.

**Fig. 3:**
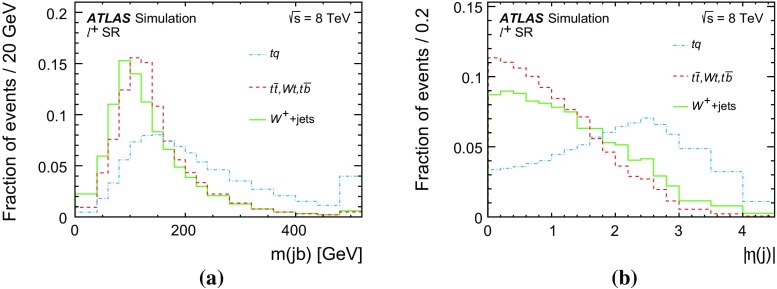
Probability densities of the two most discriminating input variables to the NN: **a** the invariant mass *m*(*jb*) of the untagged jet and the *b*-tagged jet, and **b** the absolute value of the pseudorapidity of the untagged jet $|\eta (j)|$. The distributions are shown for the *tq* signal process, the $W^{+}$ + jets background and the top-quark background in the $\ell ^{+}$ SR. Events beyond the *x*-axis range are included in the last bin

The separation between signal and the two most important backgrounds, i.e. the top-quark background and the $W$ + jets background, is illustrated in Fig. 3 for the two most discriminating variables.

**Table 2:** The seven input variables to the NN ordered by their discriminating power. The jet that is not *b*-tagged is referred to as *untagged* jet

Variable symbol	Definition
*m*(*jb*)	The invariant mass of the untagged jet (*j*) and the *b*-tagged jet (*b*)
$|\eta (j)|$	The absolute value of the pseudorapidity of the untagged jet
$m(\ell \nu b)$	The invariant mass of the reconstructed top quark
$m_{\mathrm {T}}(\ell E_{\text {T}}^{\text {miss}})$	The transverse mass of the lepton–$E_{\text {T}}^{\text {miss}}$ system, as defined in Eq. (2)
$|\Delta \eta (\ell \nu ,b)|$	The absolute value of $\Delta \eta$ between the reconstructed *W* boson and the *b*-tagged jet
$m(\ell b)$	The invariant mass of the charged lepton ($\ell$) and the *b*-tagged jet
$\cos \theta ^*(\ell ,j)$	The cosine of the angle, $\theta ^*$, between the charged lepton and the untagged jet in the rest frame of the reconstructed top quark

The training of the NN is done with a sample of simulated events that comprises events with leptons of positive and negative charge. This approach gives the same sensitivity as a scenario in which separate NNs are trained in the $\ell ^{+}$ SR and in the $\ell ^{-}$ SR. The modelling of the input variables is checked in the $W$ + jets VR and in the $t\bar{t}$ VR; see Sect. 5 for the definition. In the $t\bar{t}$ VR both jets are *b*-tagged, which poses the question how to define variables which are using the untagged jet in the SR. The two *b*-jets are sorted in $|\eta |$ and the jet with the highest $|\eta |$ is assigned to mimic the untagged jet of the SR. The distributions of all input variables are found to be well modelled in the VRs.

**Fig. 4:**
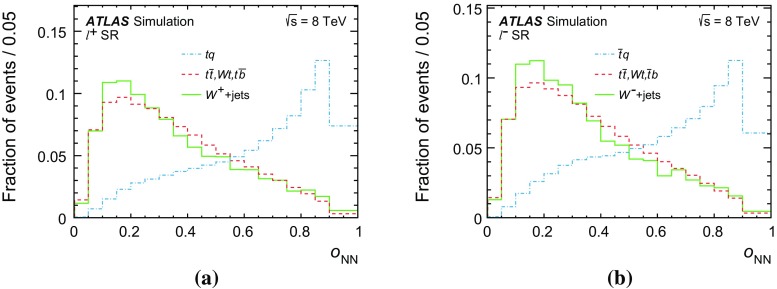
Probability densities of the NN discriminants in the signal region (SR) for the *tq* and $\bar{t}q$ signal processes, the $W$ + jets background and the top-quark background: **a** in the $\ell ^{+}$ SR and **b** in the $\ell ^{-}$ SR

In Fig. 4, the probability densities of the resulting $O_{\text {NN}}$ distributions are shown for the signal, the top-quark background, and the $W$ + jets  background.

**Fig. 5:**
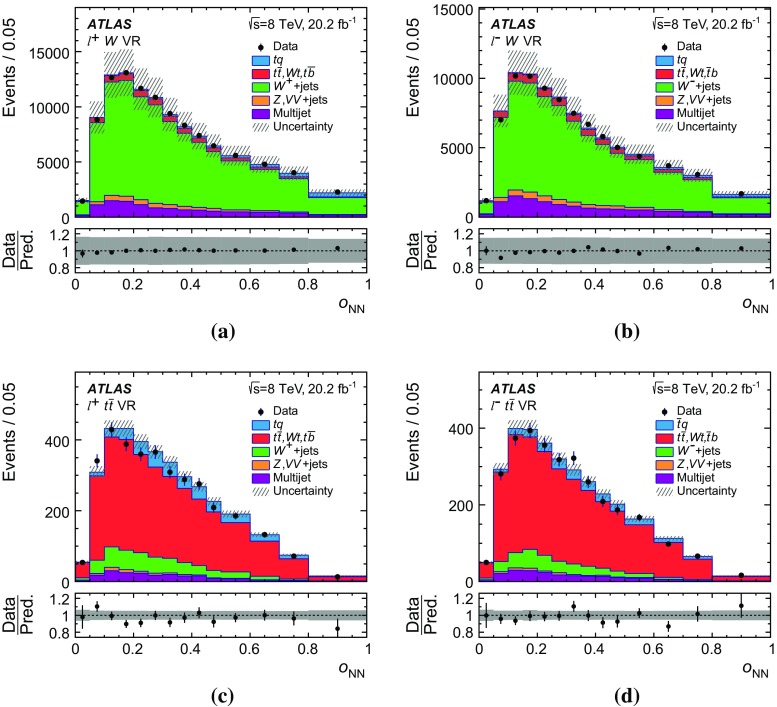
Observed $O_{\text {NN}}$ distributions (**a**, **b**) in the $W$ + jets VR and (**c**, **d**) in the $t\bar{t}$ VR compared to the model obtained from simulated events. The simulated distributions are normalised to the event rates obtained by the fits of the $E_{\text {T}}^{\text {miss}}$ distributions as described in Sect. 6. The hatched uncertainty band represents the uncertainty in the pre-fit process cross-sections and the bin-by-bin MC statistical uncertainty, added in quadrature. The *lower panels* show the ratio of the observed to the expected number of events in each bin

The modelling of collision data with simulated events is further tested by applying the NNs in the validation regions. The corresponding distributions are shown in Fig. 5. Good agreement between the model and the measured distributions is found.

## Systematic uncertainties

Many sources of systematic uncertainty affect the individual top-quark and top-antiquark cross-section measurements and their ratio. The uncertainties are split into the following categories:

**Object modelling** Systematic uncertainties due to the residual differences between data and MC simulation, for reconstructed jets, electrons and muons after calibration, and uncertainties in corrective scale factors are propagated through the entire analysis. The main source of object modelling uncertainty is the jet energy scale (JES).

Uncertainties in the lepton trigger, reconstruction, and selection efficiencies in simulations are estimated from measurements of the efficiency using $Z\rightarrow \ell ^{+}\ell ^{-}$ decays. To evaluate uncertainties in the lepton momentum scale and resolution, the same processes are used [73]. The uncertainty in the charge misidentification rates was studied and found to be negligible for this analysis.

The jet energy scale was derived using information from test-beam data, LHC collision data and simulation. Its uncertainty increases with $\eta$ and decreases with the $p_{\text {T}}$ of the reconstructed jet [53].

The JES uncertainty has various components originating from the calibration method, the calorimeter response, the detector simulation, and the specific choice of parameters in the parton shower and fragmentation models employed in the MC event generator. Additional contributions come from the modelling of pile-up effects, differences between *b*-quark-induced jets and light-quark or gluon-induced jets. Included in the JES components are also uncertainties in the flavour composition of the jets and the calorimeter response to jets of different flavours. Both JES flavour uncertainties are reduced by using actual gluon-fractions of the untagged jet obtained from simulated signal samples. A parameterisation with 22 uncorrelated components is used, as described in Ref. [53].

Small uncertainties arise from the modelling of the jet energy resolution and the missing transverse momentum, which accounts for contributions of calorimeter cells not matched to any jets, low-$p_{\text {T}}$ jets, and pile-up. The effect of uncertainties associated with the jet-vertex fraction is also considered for each jet.

Since the analysis makes use of *b*-tagging, the uncertainties in the *b*- and *c*-tagging efficiencies and the mistag rates [74, 75] are taken into account and called flavour tagging uncertainty. Since the interaction of matter and antimatter with the detector material is different, the difference in the *b*-tagging efficiency between jets initiated by a *b*-quark and a *b*-antiquark is estimated and results to be $\sim$1% based on simulated $tq$ and $\bar{t}q$ events .

**Monte Carlo generators and parton densities** Systematic uncertainties from MC modelling are estimated by comparing different generators and varying parameters for the event generation. These uncertainties are estimated for all processes involving top quarks, and taken to be correlated among the $tq$ and $\bar{t}q$ processes and uncorrelated between these two and the top-quark background ($t\bar{t}$, *Wt*, $t\bar{b}$, and $\bar{t}b$).

The uncertainty due to the choice of factorisation scale and renormalisation scale in the ME computation of the MC generators is estimated by varying these scales independently by factors of one half and two using the Powheg-Box generator. In addition, a different set of tuned parameters of the Pythia parton shower with modified $\alpha _{\text {S}}$ is used to match the scale variation in the ME. The detailed list of modified parameters is given in Ref. [36]. The uncertainty is defined by the envelope of all independent variations.

Systematic uncertainties in the matching of the NLO matrix calculation and the parton shower are estimated by comparing samples produced with MC@NLO and with Powheg-Box, in both cases interfaced to the Herwig parton shower. For the *tq* and $\bar{t} q$ processes, MadGraph5_aMC@NLO is used instead of MC@NLO.

The uncertainty from the parton shower and hadronisation modelling is estimated by comparing samples produced with Powheg-Box  + Herwig and Powheg-Box  + Pythia.

Systematic uncertainties related to the PDFs are taken into account for all processes, except for the $Z$ + jets, due to the small yield, and multijet contributions. The uncertainty is estimated following the PDF4LHC recommendation [76], using the PDF4LHC15_NLO PDF set. In addition, the acceptance difference between PDF4LHC15_NLO and CT10 is considered, since the latter PDF set is not covered by the uncertainty obtained with PDF4LHC15_NLO. The total PDF uncertainties are dominated by the acceptance differences between CT10 and PDF4LHC15_NLO. For the two signal processes the correlation coefficient of the total PDF uncertainties is found to be close to one.

Modelling uncertainties in the $W$ + jets sample are investigated using particle-level distributions obtained with the Sherpa event generator by varying simultaneously the factorisation and renormalisation scales. The corresponding fractional changes with respect to the nominal particle-level $p_{\text {T}} (W)$ distribution are applied to the reconstructed $p_{\text {T}} (W)$ distribution and modified $O_{\text {NN}}$ distributions are obtained. The effect on the measured *t*-channel cross section is found to be negligible.

Finally, the MC statistical uncertainty is included.

**Background normalisation** The uncertainties in the normalisation of the various background processes are estimated by using the uncertainties in the theoretical cross-section predictions as detailed in Sect. 6.

For the $W$ + jets and $Z$ + jets backgrounds, an uncertainty of 21% is assigned. This uncertainty is estimated based on parameter variations in the generation of the Sherpa samples. It was found that a correlated variation of the factorisation and renormalisation scales has the biggest impact on the kinematic distributions and produces variations covering the unfolded *Z* / *W* + jets data and their uncertainties [77].

The multijet background estimate has an uncertainty of 15%, based on comparisons of the default method with the yield obtained with the matrix method [69]. Additionally an uncertainty in the shape of distributions is defined in the same way.

**Luminosity** The absolute luminosity scale is derived from beam-separation scans performed in November 2012. The uncertainty in the integrated luminosity is 1.9% [29].

## Fiducial and total cross-section measurements

The signal yields $\hat{\nu }(tq)$ and $\hat{\nu }(\bar{t}q)$ (see Eq. (4)) are extracted by performing a binned maximum-likelihood fit to the $O_{\text {NN}}$ distributions in the $\ell ^{+}$ SR and in the $\ell ^{-}$ SR. The production of $tq$ and $\bar{t}q$ are treated independently. The signal rates, the rate of the combined top-quark background ($t\bar{t}$, *Wt*, $t\bar{b}$, and $\bar{t}b$), and the rate of the combined *W* + light-jets, $W+c\bar{c}$, and $W+b\bar{b}$ background, are fitted simultaneously. The rates of $W^++$ jets and $W^-+$ jets are independent parameters in the fit. The event yields of the multijet background and the $Z,VV+\mathrm {jets}$ background are fixed to the estimates given in Table 1. The multijet background is determined in a data-driven way, see Sect. 6, and is therefore not subject to the fit of the signal yields. The $Z,VV+\mathrm {jets}$ background is relatively small and cannot be further constrained by the fit.

The maximum-likelihood function is given by the product of Poisson probability terms for the individual histogram bins (see Ref. [9]). Gaussian prior probability distributions are included multiplicatively in the maximum-likelihood function to constrain the background rates, which are subject to the fit, to their predictions given the associated uncertainties. The event yields estimated in the fit are given in Table 3.

**Table 3:** Event yields for the different processes estimated with the fit to the $O_{\text {NN}}$ distribution compared to the numbers of observed events. Only the statistical uncertainties are quoted. The $Z,VV+\mathrm {jets}$ contributions and the multijet background are fixed in the fit; therefore no uncertainty is quoted for these processes

Process	$\hat{\nu }(\ell ^+)$	$\hat{\nu }(\ell ^-)$
*tq*	11,800 ± 200	17 ± 1
$\bar{t}q$	11 ± 1	6920 ± 170
$t\bar{t},Wt,t\bar{b}/\bar{t}b$	19,300 ± 740	18,900 ± 730
$W^++$ jets	18,800 ± 780	48 ± 2
$W^-+$ jets	23 ± 1	13,100 ± 740
$Z,VV+\mathrm {jets}$	1290	1190
Multijet	4520	4520
Total estimated	55,800 ± 1100	44,700 ± 1100
Data	55,800	44,687

**Fig. 6:**
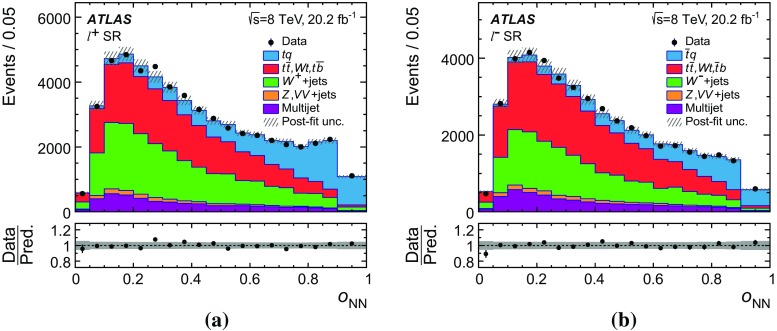
Observed $O_{\text {NN}}$ distributions in **a** the $\ell ^{+}$ SR and in **b** the $\ell ^{-}$ SR compared to the model obtained from simulated events. The simulated distributions are normalised to the event rates obtained by the fit to the discriminants. The hatched uncertainty *band* represents the total uncertainty in the rates of all processes after the fit and the bin-by-bin MC statistical uncertainty, added in quadrature. The *lower panels* show the ratio of the observed to the expected number of events in each bin to illustrate the goodness-of-fit.

In Fig. 6, the observed $O_{\text {NN}}$ distributions are shown and are compared to the compound model of signal and background normalised to the fit result.

**Fig. 7:**
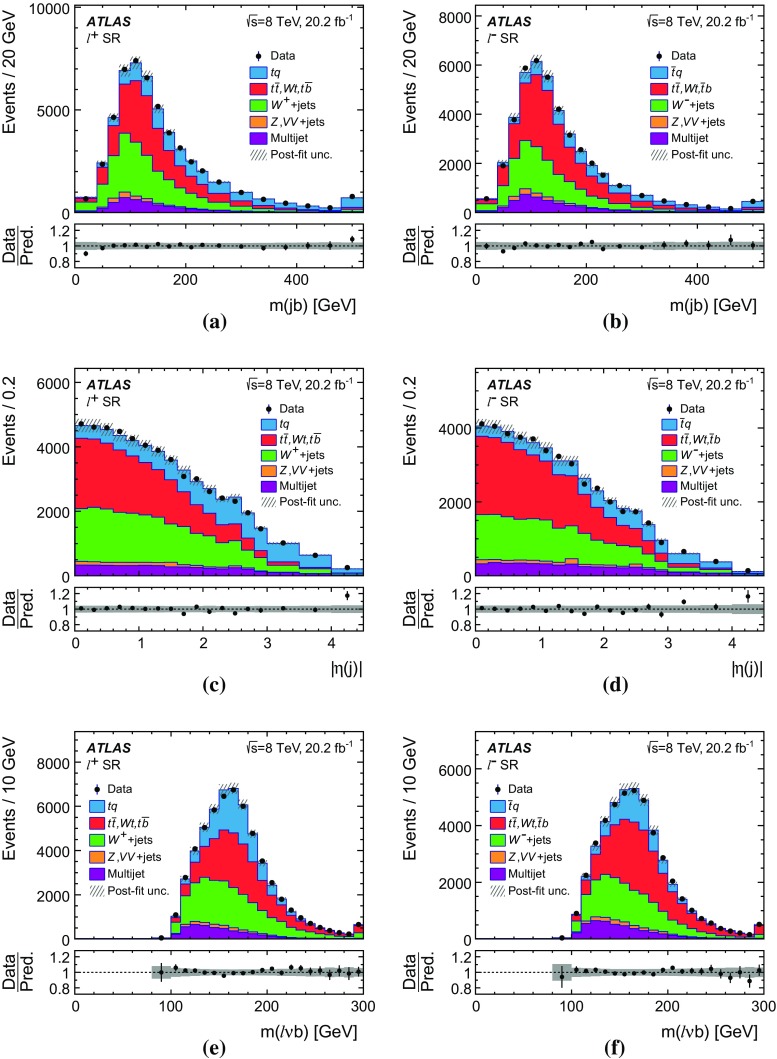
Observed distributions of the three most important input variables to the NN in the SR compared to the model obtained with simulated events. The definitions of the variables can be found in Table 2. The simulated distributions are normalised to the event rates obtained by the maximum-likelihood fit to the NN discriminants. The hatched uncertainty *band* represents the total uncertainty in the rates of all processes after the fit and the bin-by-bin MC statistical uncertainty, added in quadrature. The *lower panels* show the ratio of the observed to the expected number of events in each bin to illustrate the goodness-of-fit. Events beyond the *x*-axis range in (**a**), (**b**), (**e**) and (**f**)

Figure 7 displays the observed distributions of the three most discriminating variables compared to the distributions obtained with simulated events normalised to the fit result. Differences between data and prediction are covered by the normalisation uncertainty of the different fitted processes.

**Fig. 8:**
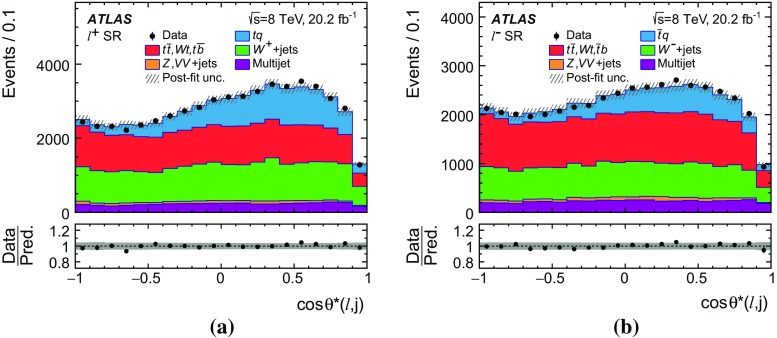
Observed distributions of $\cos \theta ^*(\ell , j)$ in **a** the $\ell ^{+}$ SR and in **b** the $\ell ^{-}$ SR compared to the model obtained from simulated events. The simulated distributions are normalised to the event rates obtained by the fit to the $O_{\text {NN}}$ distributions. The hatched uncertainty *band* represents the total uncertainty in the rates of all processes after the fit and the bin-by-bin MC statistical uncertainty, added in quadrature. The *lower panels* show the ratio of the observed to the expected number of events in each bin to illustrate the goodness-of-fit.

Since single top-quarks are produced via the charged–current weak interaction (*W*-boson exchange), they are polarised. The polarisation is most prominently visible in the distribution of $\cos \theta ^*(\ell , j)$ shown in Fig. 8. The good modelling of the observed distribution of this characteristic variable by simulated distributions scaled to the fitted event rates serves as further confirmation of the fit result.

### Fiducial cross-section measurements

The fiducial cross-sections are calculated using Eq. (6), yielding

8 $$\begin{aligned} \sigma _{\text {fid}} (tq)&= 9.78 \pm 0.16 \, (\text {stat.}) \pm 0.52 \, (\text {syst.}) \pm 0.19 \, (\text {lumi.})~\text {pb} \nonumber \\&= 9.78 \pm 0.57~\text {pb} \end{aligned}$$

and

9 $$\begin{aligned} \sigma _{\text {fid}} (\bar{t}q)&= 5.77 \pm 0.14 \, (\text {stat.}) \pm 0.41 \, (\text {syst.}) \pm 0.11 \, (\text {lumi.})~\text {pb} \nonumber \\&= 5.77 \pm 0.45~\text {pb}. \end{aligned}$$

The uncertainties in the measured expectation values of the number of signal events, $\hat{\nu }(tq)$ and $\hat{\nu }(\bar{t}q)$ in Eq. (6), are obtained from pseudo-experiments, employing the same technique as in Ref. [10], and are propagated to the measured cross-sections. The systematic uncertainties discussed in Sect. 9 cause variations of the signal acceptance, the background rates and the shape of the NN discriminant. Only significant shape uncertainties are taken into account in the statistical analysis. Shape uncertainties are considered significant if their magnitude exceeds the statistical uncertainty in at least one bin of the $O_{\text {NN}}$ distribution. In order to dampen statistical fluctuations a median filter is applied to the distribution of the bin-wise relative uncertainty. The filter uses a five-bin-wide sliding window and is by construction not applied to the first and the last two bins of a histogram. After applying this procedure, shape uncertainties are considered for the following sources: two JES uncertainty components, jet energy resolution, $E_{\text {T}}^{\text {miss}}$ modelling, the modelling of the multijet background, and all MC-generator-related uncertainties.

Since the *tq* and $\bar{t} q$ production cross-sections are measured in a fiducial region, systematic uncertainties in the event rates affect only $N_{\text {sel}}\,/N_{\text {fid}}$  in Eq. (6), thereby reducing the uncertainties related to the choice of PDF, signal MC generator and parton-shower by about 1 percentage point each. The uncertainties in the scale choice of the signal generator and the NLO matching are reduced by about 2 percentage points each. Contributions of the various sources of systematic uncertainty to the measured values of $\sigma _{\text {fid}} (tq)$ and $\sigma _{\text {fid}} (\bar{t} q)$ are shown in Table 4.

**Table 4:** Detailed list of the contribution from each source of uncertainty to the total uncertainty in the measured values of $\sigma _{\text {fid}} (tq)$ and $\sigma _{\text {fid}} (\bar{t}q)$. The estimation of the systematic uncertainties has a statistical uncertainty of 0.3%. Uncertainties contributing less than 0.5% are marked with ‘$<0.5$’

Source	$\Delta \sigma _{\text {fid}} (tq)\ / \ \sigma _{\text {fid}} (tq)$	$\Delta \sigma _{\text {fid}} (\bar{t} q)\ / \ \sigma _{\text {fid}} (\bar{t} q)$
	(%)	(%)
Data statistics	±1.7	±2.5
Monte Carlo statistics	±1.0	±1.4
Background normalisation	<0.5	<0.5
Background modelling	±1.0	±1.6
Lepton reconstruction	±2.1	±2.5
Jet reconstruction	±1.2	±1.5
Jet energy scale	±3.1	±3.6
Flavour tagging	±1.5	±1.8
$E_{\text {T}}^{\text {miss}}$ modelling	±1.1	±1.6
$b/\bar{b}$ tagging efficiency	±0.9	±0.9
PDF	±1.3	±2.2
*tq* ($\bar{t} q$) NLO matching	±0.5	<0.5
*tq* ($\bar{t} q$) parton shower	±1.1	±0.8
*tq* ($\bar{t} q$) scale variations	±2.0	±1.7
$t\bar{t}$ NLO matching	±2.1	±4.3
$t\bar{t}$ parton shower	±0.8	±2.5
$t\bar{t}$ scale variations	<0.5	<0.5
Luminosity	±1.9	±1.9
Total systematic	±5.6	±7.3
Total (stat. + syst.)	±5.8	±7.8

The relative combined uncertainties, including the statistical and systematic uncertainties, are ± 5.8% for $\sigma _{\text {fid}} (tq)$ and ± 7.8% for $\sigma _{\text {fid}} (\bar{t}q)$. The three largest sources of uncertainty are the uncertainty in the JES calibration, the choice of matching method used for the NLO generator of the top-quark background and the uncertainty in the lepton reconstruction.

**Fig. 9:**
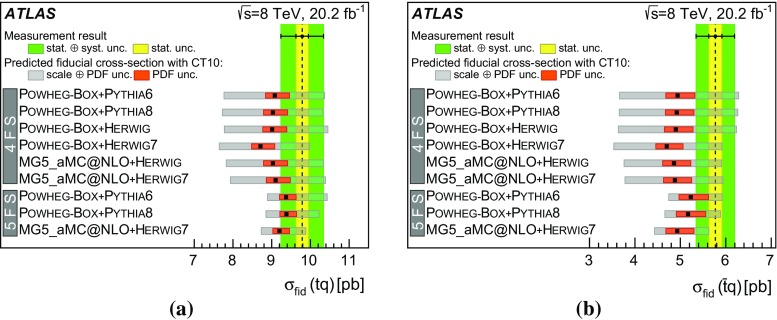
Measured *t*-channel **a** single-top-quark and **b** single-top-antiquark fiducial cross-sections compared to predictions by the NLO MC generators Powheg-Box and MadGraph5_aMC@NLO in the four-flavour scheme (4FS) and five-flavour scheme (5FS) combined with different parton-shower models. The uncertainties in the predictions include the uncertainty due to the scale choice using the method of independent restricted scale variations and the intra-PDF uncertainty in the CT10 PDF set

Figure 9 shows the measured fiducial cross-sections in comparison to the predictions by the NLO MC generators Powheg-Box and MadGraph5_aMC@NLO combined with the parton-shower programs Pythia 6  (v6.428), Pythia 8 (v8.2) [32], Herwig (v6.5.20) and Herwig 7 (v7.0.1) [78].

The 4FS and the 5FS are explored. The predictions are computed with the CT10 PDF set and include the uncertainty in the scale choice using the method of independent restricted scale variations as described in Sect. 1 and the uncertainty in the PDFs, using the intra-PDF uncertainties of CT10. The predictions based on the 5FS feature strongly reduced scale uncertainties compared to those based on the 4FS. When computing the predictions of $\sigma _{\text {fid}}$ based on Eq. (7), the uncertainties in the predictions of $\sigma _{\text {tot}}$ are treated as correlated with the scale and PDF uncertainties in $A_\text {fid}$. For the Pythia 6 parton shower the value of $\alpha _{\text {S}}$ in the set of tuned parameters is also modified consistently with the change of the scale in the ME. PDF uncertainties are obtained by reweighting to eigenvectors of their respective error sets. The predictions of all setups agree with each other and also with the measured values.

### Total cross-section measurements

**Fig. 10:**
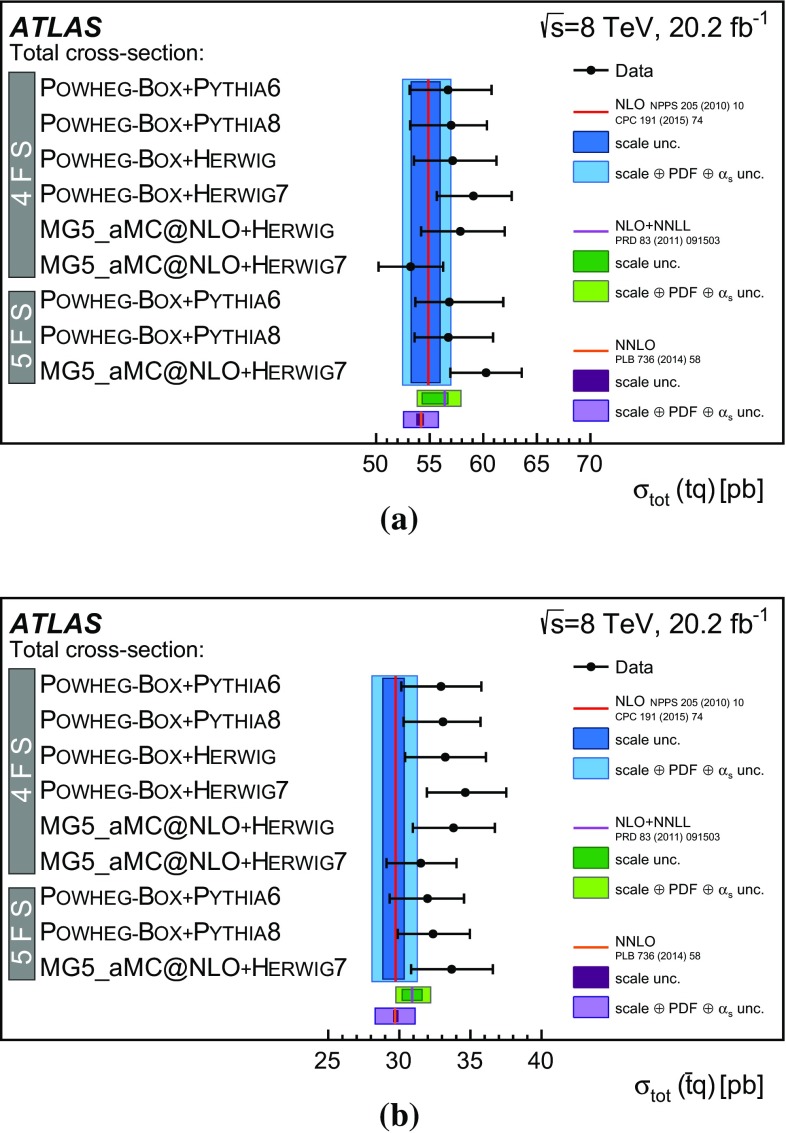
Extrapolated *t*-channel **a** single-top-quark and **b** single-top-antiquark production cross-sections for different MC-generator setups compared to fixed-order NLO calculations. For the three calculations, the uncertainty from the renormalisation and factorisation scales are indicated in *darker shading*, and the total uncertainties, including the renormalisation and factorisation scale as well as the PDF + $\alpha _{\text {S}}$ uncertainties, are indicated in *lighter shading*. For the NNLO prediction, only the renormalisation and factorisation scale uncertainty is provided in Ref. [22]. For comparison, the PDF + $\alpha _{\text {S}}$ uncertainties from the NLO prediction [14] are added to the NNLO renormalisation and factorisation scale uncertainty reflected in the *lighter shaded* uncertainty band. For this comparison, the uncertainty in the extrapolation does not include the contribution from the NLO-matching method and from the choice of parton-shower model

Using the predictions of $A_\text {fid}$ by different MC generators, the fiducial cross-sections are extrapolated to the full phase space and compared to fixed-order calculations. The PDF and scale uncertainties in $A_\text {fid}$ are included and correlated with the PDF and scale uncertainty in $\sigma _{\text {fid}}$. Figure 10 shows the total cross-sections obtained by the extrapolation, based on $A_\text {fid}$ from Powheg-Box and MadGraph5_aMC@NLO for the 4FS and 5FS and for different parton-shower MC programs. Since the extrapolation from the fiducial to the total cross-sections is performed for different MC generators, the uncertainty in the NLO-matching method and the uncertainty due to the choice of the parton-shower program are not considered for the extrapolation part, but these uncertainties are kept for the fiducial cross-sections entering the extrapolation. The measured values are compared with fixed-order perturbative QCD calculations [14, 15, 22, 23].

For the default generator Powheg-Box + Pythia 6 the fiducial acceptances are determined to be $A_\text {fid}(tq) = (17.26^{+0.46}_{-0.21})\%$ and $A_\text {fid}(\bar{t}q) = (17.52^{+0.45}_{-0.20})\%$, thereby yielding

10 $$\begin{aligned} \sigma _{\text {tot}} (tq) =&56.7 \pm 0.9 \, (\text {stat.}) \pm 2.7 \, (\text {exp.}) \,^{+2.7}_{-1.7} \, (\text {scale}) \pm 0.4 \, (\text {PDF}) \nonumber \\&\quad \pm 1.0 \, (\text {NLO-matching method}) \, \pm 1.1 \, (\text {parton shower}) \pm 1.1\,(\text {lumi.})\;\mathrm{pb} \nonumber \\ =&56.7^{+4.3}_{-3.8}\;\mathrm{pb} \end{aligned}$$

and

11 $$\begin{aligned} \sigma _{\text {tot}} (\bar{t} q) =&32.9 \pm 0.8 \, (\text {stat.}) \pm 2.3 \, (\text {exp.}) \,^{+1.4}_{-0.8} \, (\text {scale}) \pm 0.3 \, (\text {PDF}) \nonumber \\&\quad \pm \,^{+0.7}_{-0.6} \, (\text {NLO-matching method})\,\pm 0.6 \, (\text {parton shower}) \pm 0.6\,(\text {lumi.})\;\mathrm{pb} \nonumber \\ =&32.9^{+3.0}_{-2.7}\;\mathrm{pb}. \end{aligned}$$

The experimental systematic uncertainty (exp.) contains the uncertainty in the fiducial cross-sections, without the scale, PDF, NLO-matching method and parton-shower components, which are quoted separately and include both the uncertainties in $\sigma _{\text {fid}}$ and $A_\text {fid}$. The relative total uncertainty is $^{+7.6}_{-6.7}\,\%$ for $\sigma _{\text {tot}} (tq)$ and $^{+9.1}_{-8.4}\,\%$ for $\sigma _{\text {tot}} (\bar{t}q)$.

The total inclusive cross-section is obtained by adding $\sigma _{\text {tot}} (tq)$ and $\sigma _{\text {tot}} (\bar{t} q)$ in Eqs. (10) and (11):

12 $$\begin{aligned} \sigma _{\text {tot}} (tq+\bar{t} q) =&89.6 \pm 1.2 \, (\text {stat.}) \pm 5.1 \, (\text {exp.})\,^{+4.1}_{-2.5} \, (\text {scale}) \pm 0.7 \, (\text {PDF})\nonumber \\&\quad \pm \,^{+1.7}_{-1.6} \, (\text {NLO-matching method})\,\pm 1.6 \, (\text {parton shower}) \pm 1.7 \, (\text {lumi.})~\text {pb} \nonumber \\&= 89.6^{+7.1}_{-6.3}~\text {pb}. \end{aligned}$$

The systematic uncertainties are assumed to be 100% correlated between *tq* and $\bar{t} q$, except for the MC statistical uncertainty. Therefore, the uncertainties are added linearly component by component. The data statistical uncertainties of $\sigma _{\text {tot}} (tq)$ and $\sigma _{\text {tot}} (\bar{t} q)$ are added in quadrature to obtain the data statistical uncertainty of $\sigma _{\text {tot}} (tq+\bar{t} q)$. The same is done for the MC statistical uncertainty. The experimental systematic uncertainty (exp.) contains the uncertainty in the fiducial cross-sections, without the scale, PDF, NLO-matching method and parton-shower components.

### $R_t$ measurement

The ratio of the measured total cross-sections for top-quark and top-antiquark production in the *t*-channel is determined to be

13 $$\begin{aligned} R_t = \frac{\sigma _{\text {tot}} (tq)}{\sigma _{\text {tot}} (\bar{t}q)}=1.72 \pm 0.05\, (\text {stat.})\, \pm 0.07\, (\text {exp.}) = 1.72 \pm 0.09. \end{aligned}$$

The correlation of uncertainties in $\sigma _{\text {tot}} (tq)$ and $\sigma _{\text {tot}} (\bar{t}q)$ is taken into account in the pseudo-experiments used to determine the uncertainties in $\hat{\nu }(tq)$ and $\hat{\nu }(\bar{t}q)$, see Sect. 10.1. Significant sources of systematic uncertainty in the measured values of $R_t$ are shown in Table 5.

**Table 5:** Significant contributions to the total relative uncertainty in the measured value of $R_t$. The estimation of the systematic uncertainties has a statistical uncertainty of 0.3%. Uncertainties contributing less than 0.5% are not shown

Source	$\Delta R_t/ R_t$ (%)
Data statistics	±3.0
Monte Carlo statistics	±1.8
Background modelling	±0.7
Jet reconstruction	±0.5
$E_{\text {T}}^{\text {miss}}$ modelling	±0.6
$tq$ ($\bar{t}q$) NLO matching	±0.5
$tq$ ($\bar{t}q$) scale variations	±0.7
$t\bar{t}$ NLO matching	±2.3
$t\bar{t}$ parton shower	±1.7
PDF	±0.7
Total systematic	±3.9
Total (stat. + syst.)	±5.0

**Fig. 11:**
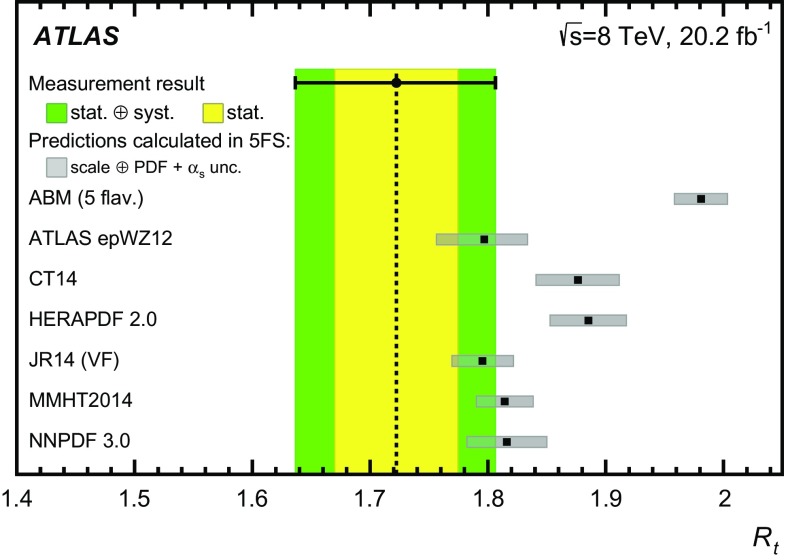
Predicted values of $R_t =\sigma _{\text {tot}} (tq)/\sigma _{\text {tot}} (\bar{t}q)$ calculated with HatHor  [14] at NLO accuracy in QCD [15] in the 5FS using different NLO PDF sets [79–85] compared to the measured value. The *error bars* on the predictions include the uncertainty in the renormalisation and factorisation scales and the combined internal PDF and $\alpha _{\text {S}}$ uncertainty. The *dashed black line* indicates the central value of the measured $R_t$ value. The combined statistical and systematic uncertainty of the measurement is shown in *green*, while the statistical uncertainty is represented by the *yellow error band*. The uncertainty in the measured $R_t$ value does not include the PDF components for this comparison

Figure 11 compares the observed value of $R_t$ to predictions based on several different PDFs. For this comparison the uncertainty in the measured $R_t$ value does not include the PDF components. The uncertainties in the predictions include the uncertainty in the renormalisation and factorisation scales and the combined internal PDF and $\alpha _{\text {S}}$ uncertainty. Most predictions agree at the $1\,\sigma$ level with the measured value; only the prediction based on ABM (5 flav.) [79] is about $2.5\,\sigma$ above the measurement. The main differences of the ABM PDF set compared to the other sets are the treatment of the *b*-quark PDF and the value of $\alpha _{\text {S}}$.

### Estimation of top-quark mass dependence

The *t*-channel cross-section results given above are obtained for a top-quark mass of $$m_t = {172.5}\,\mathrm{GeV}$$. The dependence of the measured cross-sections on $$m_t $$ is estimated by repeating the measurement with different mass assumptions. The MC samples for all processes containing top quarks are reproduced for six different values of $$m_t $$, namely 165, 167.5, 170, 175, 177.5 and 180 GeV. The samples comprise the $$tq$$ and $$\bar{t}q$$ signal as well as the background samples for $$t\bar{t}, Wt, t\bar{b}$$ production. The dependences of the resulting cross-sections on $$m_t $$ are fitted with a first-order polynomial, for which the constant term is given by the central value at $$m_t = {172.5}\,{\rm GeV}$$

14 $$\begin{aligned} \sigma (m_t) = \sigma ({172.5}\, {\rm GeV}) + a \cdot \Delta m_t [\mathrm{GeV}], \end{aligned}$$

where $\Delta m_t = m_t- {172.5}\,\mathrm{GeV}$. The fitted parameters *a*, the slopes, are given in Table 6 for all measured cross-sections.

**Table 6:** Slopes *a* of the mass dependence of the measured cross-sections

Measurement	$a\left[ \frac{\mathrm{pb}}{\mathrm{GeV}}\right]$
$\sigma _{\text {fid}}(tq)$	$-0.06\pm 0.01$
$\sigma _{\text {fid}}(\bar{t} q)$	$-0.04\pm 0.01$
$\sigma _{\text {tot}}(tq)$	$-0.59\pm 0.08$
$\sigma _{\text {tot}}(\bar{t} q)$	$-0.37\pm 0.06$
$\sigma _{\text {tot}}(tq+\bar{t} q)$	$-0.96\pm 0.13$

### Determination of $|V_{tb}|$

Single top-quark production in the *t*-channel proceeds via a *Wtb* vertex and the measured cross-section is proportional to $f_{\text {LV}}^2 \cdot |V_{tb}|^2$. In the SM, $|V_{tb}|$ is very close to one and $f_{\text {LV}}$ is exactly one, but new-physics contributions could alter the value of $f_{\text {LV}}$ significantly. The determination of $f_{\text {LV}} \cdot |V_{tb}|$ based on single-top-quark cross-section measurements is independent of assumptions about the number of quark generations and the unitarity of the CKM matrix. The only assumptions required are that $|V_{tb}|\gg |V_{td}|,|V_{ts}|$ and that the *Wtb* interaction involves a left-handed weak coupling as in the SM.

The value of $f_{\text {LV}}^2 \cdot |V_{tb}|^2$ is extracted by dividing the measured total inclusive cross-section $\sigma _{\text {tot}} (tq+\bar{t}q)$ by the SM expectation given in Eq. (1c). When calculating $f_{\text {LV}}^2 \cdot |V_{tb}|^2$, the experimental and theoretical uncertainties are added in quadrature. The uncertainty in $m_t$ is also considered, assuming $\Delta m_t = {\pm 1}\,\mathrm{GeV}$. The result obtained is

15 $$\begin{aligned} f_{\text {LV}} \cdot |V_{tb}| =&1.029 \pm 0.007 \, (\text {stat.}) \nonumber \\&\quad \pm 0.029 \, (\text {exp.}) \,^{+0.023}_{-0.014} \, (\text {scale})\pm 0.004 \, (\text {PDF})\nonumber \\&\quad \pm 0.010 \, (\text {NLO-matching method})\nonumber \\&\quad \pm 0.009 \, (\text {parton shower}) \pm 0.010 \, (\text {lumi.})\nonumber \\&\quad \pm 0.005 \, (m_t) \pm 0.024 \, (\text {theor.}) \nonumber \\ =&1.029\pm 0.048\,. \end{aligned}$$

The uncertainty in $f_{\text {LV}} \cdot |V_{tb}|$ is broken down in the first terms, reflecting the uncertainties in the combined total cross-section, as well as the uncertainty in the top-quark mass and the uncertainty in the theoretical cross-section calculation. The result is in full agreement with the SM prediction. Restricting the range of $|V_{tb}|$ to the interval [0, 1] and assuming $f_{\text {LV}} =1$, as required by the SM, a lower limit on $|V_{tb}|$ is extracted: $|V_{tb}|>0.92$ at 95% confidence level.

## Differential cross-section measurements

The measured differential distributions are unfolded, so that they can be directly compared to theoretical predictions. Two sets of unfolded cross-sections are derived: particle level and parton level. Particle-level cross-sections are measured in the fiducial volume defined in Sect. 7. Parton-level cross-sections are measured in the whole kinematic range using the MC simulation to extrapolate from the acceptance phase space. Particle-level cross-sections are measured as a function of the transverse momentum, $p_{\text {T}} (\hat{t})$, and absolute value of the rapidity, $|y(\hat{t})|$, of the pseudo top quark and pseudo top antiquark. In addition, they are measured as a function of the transverse momentum, $p_{\text {T}} (\hat{j})$, and the absolute value of the rapidity, $|y(\hat{j})|$, of the accompanying jet in the *t*-channel exchange, by assuming this jet is the untagged jet in the event. Parton-level cross-sections are measured as a function of the transverse momentum, $p_{\text {T}} (t)$, and absolute value of the rapidity, $|y(t)|$, of the top quark and top antiquark.

Differential cross-sections are extracted from an event sample enriched in signal events, which is obtained by cutting on $O_{\text {NN}}$. The cut value is set to $O_{\text {NN}} > 0.8$ (see Fig. 6), which achieves a good signal-to-background ratio and thereby reduces the impact of the systematic uncertainties on the backgrounds, while maintaining enough data events to keep the data statistical uncertainties at an acceptable level.

**Fig. 12:**
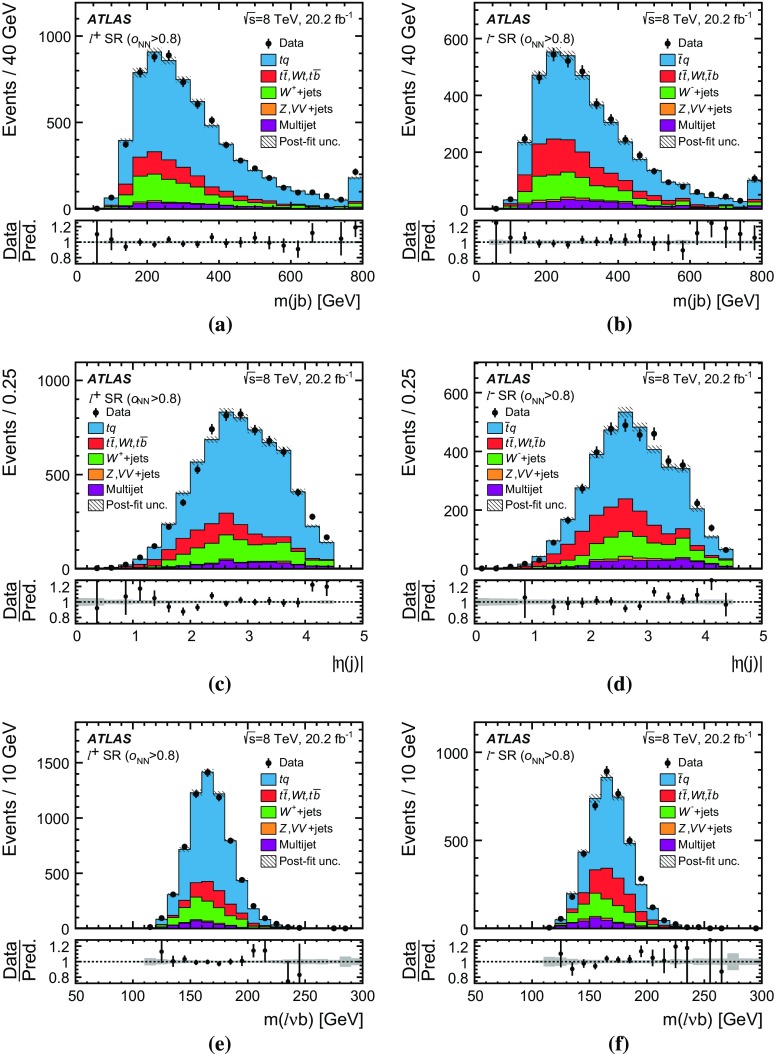
Observed distributions of the first three input variables to the default neural network in the signal region (SR), after a cut of $O_{\text {NN}} > 0.8$ on the network output. The distributions are compared to the model obtained from simulated events. The simulated distributions are normalised to the event rates obtained by the fit to the discriminants. The definitions of the variables can be found in Table 2. The hatched uncertainty *band* represents the total uncertainty in the rates of all processes after the fit and the bin-by-bin MC statistical uncertainty, added in quadrature. Events beyond the *x*-axis range in **a** and **b** are included in the last bin. The *lower panels* show the ratio of the observed to the expected number of events in each bin to illustrate the goodness-of-fit

Table 7 lists the numbers of events after the selection, including the cut on $O_{\text {NN}}$, separated into the $\ell ^{+}$ SR and the $\ell ^{-}$ SR. Both signal and backgrounds, except for the multijet background, are normalised to their fit value resulting from the binned maximum-likelihood fit to the whole $O_{\text {NN}}$ distribution, which was used to extract the total *t*-channel cross-sections described in Sect. 10. The multijet background normalisation is derived from the fit to the $E_{\text {T}}^{\text {miss}}$ distribution described in Sect. 6. Distributions of the three most discriminating input variables to the default NN (introduced in Sect. 8) after the cut on $O_{\text {NN}}$ are shown in Fig. 12.

**Table 7:** Predicted (post-fit) and observed event yields for the signal region (SR), after the requirement on the neural network discriminant, $O_{\text {NN}} > 0.8$. The multijet background prediction is obtained from the fit to the $E_{\text {T}}^{\text {miss}}$ distribution described in Sect. 6, while all the other predictions and uncertainties are derived from the total cross-section measurement. An uncertainty of 0 means that the value is $<0.5$

Process	$\ell ^{+}$ SR $(O_{\text {NN}} > 0.8)$	$\ell ^{-}$ SR $(O_{\text {NN}} > 0.8)$
$tq$	4470 ± 180	5 ± 0
$\bar{t}q$	3 ± 0	2270 ± 130
$t\bar{t}, Wt, t\bar{b}/\bar{t}b$	754 ± 45	753 ± 45
$W^{+}$ + jets	960 ± 190	1 ± 0
$W^{-}$ + jets	1 ± 0	610 ± 120
*Z*, *VV* + jets	52 ± 10	60 ± 12
Multijet	291 ± 46	267 ± 39
Total estimated	6540 ± 270	3960 ± 190
Data	6567	4007

**Table 8:** Predicted (post-fit) and observed event yields for the signal region (SR), after the requirement on the second neural network discriminant, $O_{\text {NN2}} > 0.8$. The multijet background prediction is obtained from the fit to the $E_{\text {T}}^{\text {miss}}$ distribution described in Sect. 6, while all the other predictions and uncertainties are taken from the total cross-section measurement. An uncertainty of 0 means that the value is $<0.5$

Process	$\ell ^{+}$ SR $(O_{\text {NN2}} > 0.8)$	$\ell ^{-}$ SR $(O_{\text {NN2}} > 0.8)$
$tq$	3440 ± 140	3 ± 0
$\bar{t}q$	2 ± 0	1860 ± 100
$t\bar{t}, Wt, t\bar{b}/\bar{t}b$	1072 ± 64	1057 ± 63
$W^{+}$ + jets	770 ± 150	0 ± 0
$W^{-}$ + jets	0 ± 0	494 ± 99
*Z*, *VV* + jets	43 ± 9	48 ± 10
Multijet	192 ± 30	186 ± 27
Total estimated	5520 ± 220	3650 ± 160
Data	5546	3647

**Fig. 13:**
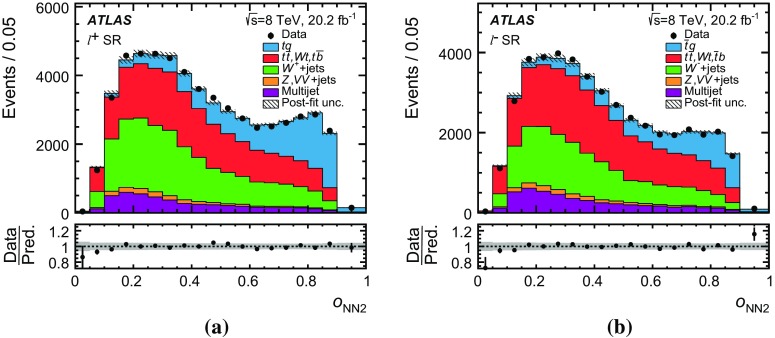
Neural network output distribution ($O_{\text {NN2}}$) of the neural network without $|\eta (j)|$ normalised to the fit results of the default network for **a** the $\ell ^{+}$ and **b** the $\ell ^{-}$ signal region (SR). The distributions are compared to the model obtained from simulated events. The simulated distributions are normalised to the event rates obtained by the fit to the discriminants. The hatched uncertainty *band* represents the total uncertainty in the rates of all processes after the fit and the bin-by-bin MC statistical uncertainty, added in quadrature.

For the measurement of the $|y(\hat{j})|$ distribution, a second neural network (NN2) is trained omitting the variable $|\eta (j)|$, in order to reduce the distortion of the $|y(\hat{j})|$ distribution as a result of cutting on the NN output. The distribution of the neutral network output variable $O_{\text {NN2}}$ is shown in Fig. 13 for both the $\ell ^{+}$ and $\ell ^{-}$ signal regions.

A cut $O_{\text {NN2}} > 0.8$ is placed on the NN output to select the events used in the unfolding. The event yields after the event selection with this network are shown in Table 8.

Very good agreement between the data and the predictions can be seen for both networks, indicating that the variables are also well described in the region where signal dominates.

**Fig. 14:**
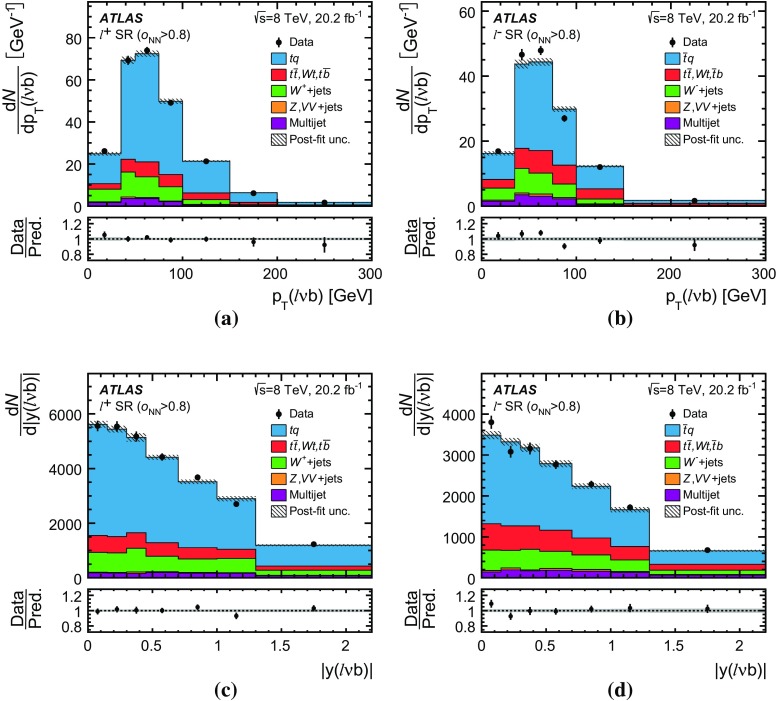
Measured distributions of (**a**, **b**) $p_{\text {T}} (\ell \nu b)$ and (**c**, **d**) $|y(\ell \nu b)|$ for (**a**, **c**) $\ell ^{+}$ and (**b**, **d**) $\ell ^{-}$ events in the signal region (SR) after a cut of $O_{\text {NN}} > 0.8$. The distributions are compared to the model obtained from simulated events. The simulated distributions are normalised to the event rates obtained by the fit to the discriminants. The hatched uncertainty *band* represents the total uncertainty in the rates of all processes after the fit and the bin-by-bin MC statistical uncertainty, added in quadrature. The *lower panels* show the ratio of the observed to the expected number of events in each bin to illustrate the goodness-of-fit

**Fig. 15:**
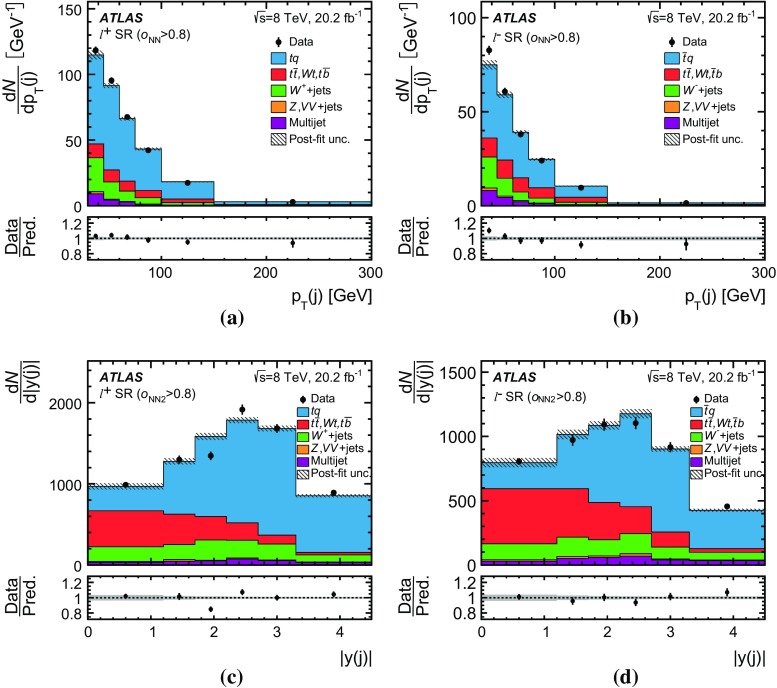
Measured distributions of (**a**, **b**) $p_{\text {T}} (j)$ and (**c**, **d**) $|y(j)|$ at reconstruction level for (**a**, **c**) $\ell ^{+}$ and (**b**, **d**) $\ell ^{-}$ events in the signal region (SR) after a cut of $O_{\text {NN}} (O_{\text {NN2}}) > 0.8$ The distributions are compared to the model obtained from simulated events. The simulated distributions are normalised to the event rates obtained by the fit to the discriminants. The hatched uncertainty *band* represents the total uncertainty in the rates of all processes after the fit and the bin-by-bin MC statistical uncertainty, added in quadrature. The *lower panels* show the ratio of the observed to the expected number of events in each bin to illustrate the goodness-of-fit

The measured differential distributions used in the unfolding are shown in Figs. 14 and 15.

Normalised differential cross-sections are evaluated by dividing the cross-section in each bin by the sum of the cross-sections in all bins for a given variable. The uncertainty in the normalised cross-section in each bin is determined from the coherent variation of the cross-section in that bin and the total cross-section when a variation reflecting a systematic uncertainty is applied.

### Unfolding technique

D’Agostini’s iterative approach [86], implemented in RooUnfold [87], is used to unfold the distributions. The method is based on picturing the problem with an “effect” and a “cause”. The number of reconstructed measured *t*-channel single-top-(anti)quark events in bin *j* is the effect, while the number of produced *t*-channel events in a *pp* collision in bin *k*, $N_{k}$, corresponds to the cause. As indicated, the bins of the measured distribution are labelled with *j*, while the bins of the generator-level distribution are labelled with *k*.

The unfolding starts from the reconstructed measured distributions. The aim is to correct these distributions for resolution and efficiency effects. The observed number of events in each bin *j* of the measured distribution can be described by:

16 $$\begin{aligned} N^{\text {data}}_j = \sum _k M_{jk} \epsilon _k L_{\text {int}}\cdot \mathrm {d}\hat{\sigma }_k + \hat{B}_j, \end{aligned}$$

where $\mathrm {d}\hat{\sigma }_k$ is the estimated cross-section in each bin *k*, $M_{jk}$ is the migration matrix, $\epsilon _k$ is the efficiency for an event to be selected in bin *k* and $\hat{B}_j$ is the sum of all background contributions.

**Fig. 16:**
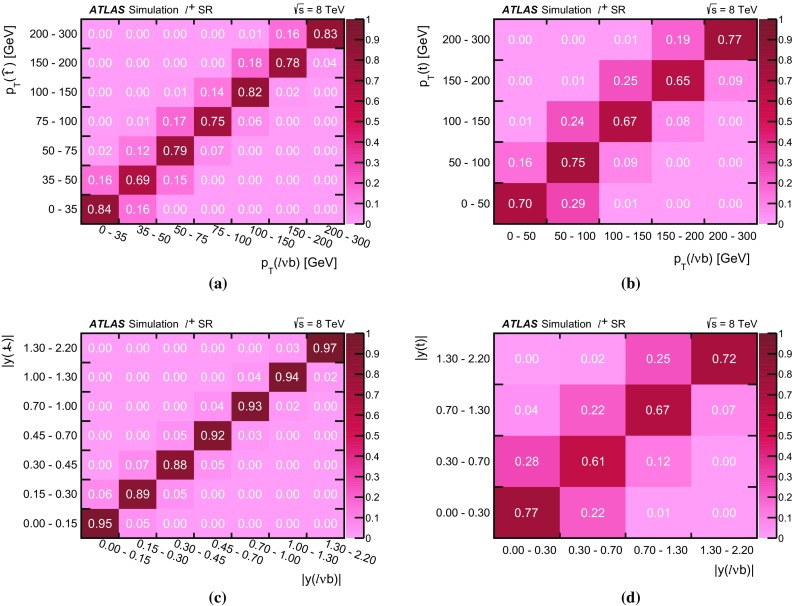
Migration matrices for **a** $p_{\text {T}} (\hat{t})$, **b** $p_{\text {T}} (t)$, **c** $|y(\hat{t})|$ and **d** $|y(t)|$. **a**, **c** Particle level, while **b** and **d** are for parton level. The pseudo top quark or parton-level quark is shown on the *y*-axis and the reconstructed variable is shown on the *x*-axis

The migration matrix describes the probability of migration of generator-level events in bin *k* to bin *j* after detector reconstruction of the event. Migration matrices, determined with the Powheg-Box + Pythia 6 MC sample, for $p_{\text {T}} (\hat{t})$ and $|y(\hat{t})|$ at particle level and $p_{\text {T}} (t)$ and $|y(t)|$ at parton level are shown in Figure 16.

The advantage of unfolding to particle level can clearly be seen; the sizes of the off-diagonal elements in the particle-level migration matrices are much smaller, which makes the unfolding less sensitive to the effect of systematic uncertainties.

The efficiency, $\epsilon _{k}$, includes signal acceptance, detector efficiencies due to e.g. trigger and *b*-tagging, as well as the efficiency of the cut on the NN output:

17 $$\begin{aligned} \epsilon _k = \frac{S_{k}^{\text {sel,MC}}}{S_k^{\text {tot,MC}}}, \end{aligned}$$

where $S_k^{\text {tot,MC}}$ is the number of generated MC events in bin *k* and $S_{k}^{\text {sel,MC}}$ is the number of selected MC events in bin *k* after all cuts are applied.

$\hat{B}_j$ is calculated from the estimated number of background events, $\tilde{\nu }_j^b$, resulting from the binned maximum-likelihood fit of the total cross-section measurement:

18 $$\begin{aligned} \hat{B}_j = \sum _{b\in \text {all background}} \tilde{\nu }_j^b\,. \end{aligned}$$

#### Unfolding to particle level

The reconstructed observables of both top quarks and untagged jets are unfolded to the particle level within the fiducial volume. The detector efficiency and resolution effects are corrected using

19 $$\begin{aligned} \hat{\nu }_k^{\text {ptcl}} = \mathcal {C}_k^{\text {ptcl!reco}} \sum _j M^{-1}_{jk} \mathcal {C}_j^{\text {reco!ptcl}} (N^{\text {data}}_j - \hat{B}_j), \end{aligned}$$

where $\hat{\nu }_k^{\text {ptcl}}$ is the measured expectation value for the number of signal events at particle level in bin *k* of the fiducial volume, $M^{-1}_{jk}$ represents the Bayesian unfolding procedure, and $\mathcal {C}_j^{\text {reco!ptcl}}$ is a correction factor for signal events that pass the reconstruction-level selection but not the particle-level selection. It is defined as

20 $$\begin{aligned} \mathcal {C}_j^{\text {reco!ptcl}} = \frac{S^{\text {reco}}_j - S^{\text{ reco!ptcl }}_j}{S^{\text {reco}}_j}, \end{aligned}$$

where $S^{\text {reco}}_j$ is the number of reconstructed signal events in bin *j* and $S^{\text {reco!ptcl}}_j$ is the number of events that pass the reconstruction-level selection but not the particle-level selection. $\mathcal {C}_k^{\text {ptcl!reco}}$ is a correction factor that accounts for signal events that pass the particle-level selection but not the reconstruction-level selection:

21 $$\begin{aligned} \mathcal {C}_k^{\text {ptcl!reco}} = \frac{1}{\epsilon _{k}} = \frac{S^{\text {ptcl}}_k}{S^{\text {ptcl}}_k - S^{\text {ptcl!reco}}_k}, \end{aligned}$$

where $S^{\text {ptcl}}_k$ is the number of signal events at particle level and $S^{\text {ptcl!reco}}_j$ is the number of events that pass the particle-level selection but not the reconstruction-level selection. The cross-section in bin *k* is evaluated from

22 $$\begin{aligned} \mathrm {d}\hat{\sigma }_k = \hat{\nu }_k^{\text {ptcl}} / L_{\text {int}}. \end{aligned}$$

For following iterations, the estimated number of events, $\hat{\nu }_k^{\text {ptcl}}$, is used as input.

#### Unfolding to parton level

The differential cross-section at parton level is determined in a way similar to that for particle level using

23 $$\begin{aligned} \mathrm {d}\hat{\sigma }_k = \frac{\sum _j M_{jk}^{-1}(N^{\text {data}}_j - \hat{B}_j)}{\epsilon _k L_{\text {int}}}, \end{aligned}$$

which can be obtained from Eqs. (19) and (22) by replacing the particle-level quantity $\mathcal {C}_k^{\text {ptcl!reco}}$ by $1/\epsilon _k$ and by omitting $\mathcal {C}_j^{\text {reco!ptcl}}$, since the parton-level cross-section is fully inclusive and such a correction is not needed.

### Binning and convergence of unfolding

The migration matrices and efficiencies determined with the Powheg-Box + Pythia 6 MC sample are used to extract the central values of the differential cross-sections. A number of criteria are used to optimise the binning chosen for each differential cross-section. These include the resolution of the measured quantity, the number of events available in the bin and the size of the diagonal elements in the migration matrix. In general, the same binning is used for $tq$ and $\bar{t} q$ cross-sections, except in a few cases when two bins are combined for $\bar{t} q$ cross-sections due to large statistical uncertainties. The resolution of kinematic quantities of the pseudo top quark is better than the resolution of the corresponding quantities at parton level. Hence more bins are usually used for the particle-level cross-sections.

The number of iterations needed before the unfolding converges depends on both the shape of the distribution being measured and the resolution of the variable. The cross-sections as a function of rapidity usually require fewer iterations before convergence, while the cross-sections as a function of $p_{\text {T}} (\hat{t})$ need the largest number of iterations, as the cross-section falls steeply and has a peak at low $p_{\text {T}}$. The criterion chosen for convergence is that the bias of the unfolded cross-section, i.e. the difference between the unfolded result and the true distribution, should be less than 1% in all bins. The bias is determined from the difference between the unfolded result using the MadGraph5_aMC@NLO + Herwig MC sample for unfolding and its generated distribution, while using the nominal Powheg-Box + Pythia 6 MC sample for the migration matrix and efficiency. Depending on the distribution being unfolded between three and nine iterations are used.

### Uncertainties

This section describes how the statistical and systematic uncertainties are propagated through the unfolding. The uncertainty from each source is estimated individually and separately for signal and background, taking correlations into account. In addition, an uncertainty is assigned to the unfolding process. All uncertainties are added in quadrature in each bin.

Systematic uncertainties enter the analysis in several places. First, they affect the background yield and therefore the expected signal-to-background ratio. The expected background is subtracted from data leading to a change in the input to the unfolding. The migration matrix and differential efficiency measured using the signal MC sample are also affected by systematic uncertainties.

For uncertainties associated with the modelling of the *t*-channel process, the bias is taken as the uncertainty. The bias is defined as the difference between the measured unfolded cross-section using a particular combination of signal, migration matrix and efficiency, and the generator-level cross-section.

#### Statistical uncertainties

The statistical uncertainty of the unfolded data result is determined by running over an ensemble of pseudo-experiments, varying the content of each bin according to its expected statistical uncertainty. Each pseudo-experiment is unfolded and the spread (RMS) of the result in each bin is taken as the measure of the statistical uncertainty.

For the statistical uncertainty due to the size of the signal MC sample, the migration matrix and efficiency are fluctuated in pseudo-experiments with a Gaussian function whose spread corresponds to the number of MC events in the sample. The unfolding is performed with each varied migration matrix and efficiency. Again the RMS of the unfolded results in each bin is taken as the uncertainty.

#### Systematic uncertainties

The list of systematic uncertainties considered and their definition is given in Sect. 9. Different uncertainties need to be treated in different ways in the unfolding. If an uncertainty is correlated between signal and background, the effect is added linearly. The methods used are described below.

**Detector-related uncertainties affecting the signal** The effects of the detector-related uncertainties affecting the signal are evaluated by unfolding the varied MC signal distributions using the nominal migration matrix and efficiency. The difference from the unfolded distribution using the nominal signal MC sample as an input is taken as the uncertainty and propagated binwise to the measurement. Thus, rate and shape uncertainties are taken into account simultaneously.

**PDF uncertainties affecting the signal** The effect of the PDF uncertainty on the *t*-channel MC simulation is evaluated by unfolding the MC signal distribution, using migration matrices and efficiencies created from different PDF MC signal sets: CT10 and the PDF4LHC15 combined PDF set. The bias of each PDF is then calculated and the largest difference is taken as both the negative and positive PDF uncertainty bin by bin. The difference between the bias of each eigenvector of the PDF4LHC15 and the bias of the central PDF4LHC15 is taken as an additional uncertainty.

**Signal modelling uncertainties** To evaluate the effect of different MC generators for the *t*-channel production, the MC signal distribution is unfolded using a migration matrix and efficiency created using either the MC signal of MadGraph5_aMC@NLO + Herwig or the MC signal of Powheg-Box + Herwig. The full difference between the bias of MadGraph5_aMC@NLO + Herwig and the bias of Powheg-Box + Herwig is assigned as systematic uncertainty. For the uncertainty associated with the parton-shower model, the full difference between the bias of Powheg-Box + Pythia 6 and the bias of Powheg-Box + Herwig is assigned as the final uncertainty. The bias of the up/down scale choice with Powheg-Box + Pythia 6 is used to estimate the uncertainty due to the scale variations.

**Uncertainties in background rates** The normalisation uncertainties of all backgrounds are taken from the total cross-section measurements. These uncertainties are listed in Table 9. The uncertainty in the sum of backgrounds is estimated using pseudo-experiments, and thus takes correlations into account. The rate uncertainty of the background sum is applied by varying the background sum up and down by the amount estimated in the total fiducial cross-section measurements. The modified background-subtracted data is unfolded with the nominal migration matrix and efficiency. The difference from the default unfolded distribution is taken as the rate uncertainty.

**Table 9:** Uncertainties in the normalisations of the different backgrounds for all processes, as derived from the total cross-section measurement

Process	$\Delta N / N$ (%)
$t\bar{t}, Wt, t\bar{b}$	7.5
$W^{+}$ + jets	7.1
$W^{-}$ + jets	7.3
*Z*, *VV* + jets	20
Multijets	16

**Uncertainties in shape of backgrounds** The uncertainty in the differential cross-sections due to the uncertainty in the shape of the background is determined by evaluating the effect of the uncertainty in the NN output for each background contribution. Some of the systematic uncertainties have a very small effect on the analysis. Hence, the shifts due to the variations reflecting the systematic uncertainties are compared to the MC statistical error in each bin of each distribution, in order to avoid counting statistical fluctuations as a systematic uncertainty. If the change in the bin content in at least two bins is larger than the MC statistical error in those bins, the background shape uncertainty is taken into account. The shifted backgrounds are subtracted from the data and the resulting distribution is unfolded using the nominal migration matrix and efficiency. The difference from the measured unfolded distribution in each bin is assigned as the systematic uncertainty due to shape. The main contribution to the shape uncertainty comes from the $t\bar{t}$ modelling.

**Unfolding uncertainty** In order to estimate the uncertainty due to the unfolding method, the Powheg-Box + Pythia 6 sample is divided into two. One half is used to determine the migration matrix, while the other half is used to unfold the cross-section. The full difference between the unfolded MC *t*-channel distribution and the MC *t*-channel generator-level distribution is taken as the uncertainty in the unfolding process.

As a cross-check, the results are compared with using a bin-by-bin correction factor and the single value decomposition (SVD) method [88], which is an extension of a simple matrix inversion. Consistent results are found and no extra uncertainty is assigned.

### Particle-level cross-sections

**Fig. 17:**
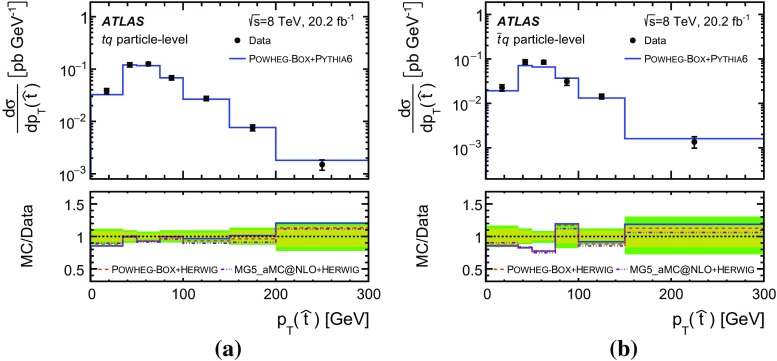
Absolute unfolded differential cross-sections as a function of $p_{\text {T}} (\hat{t})$ for **a** top quarks and **b** top antiquarks. The unfolded distributions are compared to various MC predictions. The *vertical error bars* on the data points denote the total uncertainty. The *inner* (*yellow*) *band* in the *bottom part* of each figure represents the statistical uncertainty of the measurement, and the *outer* (*green*) *band* the total uncertainty.

**Fig. 18:**
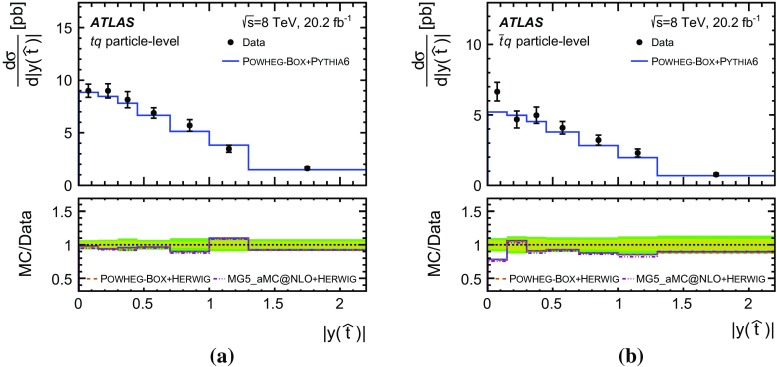
Absolute unfolded differential cross-sections as a function of $|y(\hat{t})|$ for **a** top quarks and **b** top antiquarks. The unfolded distributions are compared to various MC predictions. The *vertical error bars* on the data points denote the total uncertainty. The *inner* (*yellow*) *band* in the *bottom part* of each figure represents the statistical uncertainty of the measurement, and the *outer* (*green*) *band* the total uncertainty.

The absolute unfolded particle-level cross-sections for top quarks and top antiquarks as a function of $p_{\text {T}} (\hat{t})$ are shown in Fig. 17, while the cross-sections as a function of $|y(\hat{t})|$ are shown in Fig. 18. The numerical values of both the absolute and normalised unfolded cross-sections are given in Tables 10, 11, 12, 13. The measurements are compared to MC predictions using the Powheg-Box and MadGraph5_aMC@NLO generators. Good agreement between the measured differential cross-sections and the predictions is seen. Separate predictions using Pythia or Herwig interfaced to Powheg-Box are shown. The ratio plots show that the hadronisation model has a very small effect on the predictions.

**Table 10:** Absolute and normalised unfolded differential $tq$ production cross-section as a function of $p_{\text {T}} (\hat{t})$ at particle level

$p_{\text {T}} (\hat{t})$	$\mathrm {d}\sigma (tq)/\mathrm {d}p_{\text {T}} (\hat{t})$		$(1/\sigma ) \mathrm {d}\sigma (tq)/\mathrm {d}p_{\text {T}} (\hat{t})$	
[GeV]	[fb GeV$^{-1}$]		[10$^{-3}$ GeV$^{-1}$]	
	Stat.	Syst.	Stat.	Syst.
0–35	$38.0\pm 3.1$	$+3.3/-3.4$	$3.85\pm 0.29$	$+0.22/-0.22$
35–50	$120.9 \pm 8.4$	$+8.0 /-8.2$	$12.24\pm 0.82$	$+0.61/-0.59$
50 - 75	125.2 ± 5.3	$+7.7 / -7.9$	12.67 ± 0.49	$+0.54 / -0.54$
75–100	68.1 ± 3.9	$+5.1 / -5.0$	6.89 ± 0.38	$+0.36 / -0.34$
100–150	27.5 ± 1.5	$+2.1 /-2.1$	2.78 ± 0.15	$+0.18 / -0.18$
150–200	7.55 ± 0.76	$+0.67 / -0.56$	0.765 ± 0.076	$+0.056 /-0.046$
200–300	1.50 ± 0.24	$+0.23 / -0.23$	0.152 ± 0.023	$+0.022 /-0.022$

**Table 11:** Absolute and normalised unfolded differential $\bar{t}q$ production cross-section as a function of $p_{\text {T}} (\hat{t})$ at particle level

$p_{\text {T}} (\hat{t})$	$\mathrm {d}\sigma (\bar{t}q)/\mathrm {d}p_{\text {T}} (\hat{t})$		$(1/\sigma ) \mathrm {d}\sigma (\bar{t}q)/\mathrm {d}p_{\text {T}} (\hat{t})$	
[GeV]	[fb GeV$^{-1}$]		[10$^{-3}$ GeV$^{-1}$]	
	Stat.	Syst.	Stat.	Syst.
0–35	22.5 ± 2.7	$+2.5 / -2.4$	3.82 ± 0.44	$+0.27 /-0.24$
35–50	85.6 ± 7.8	$+7.2 /-6.3$	14.6 ± 1.3	$+1.0 /-0.8$
50–75	84.7 ± 4.7	$+5.4 / -6.9$	14.41 ± 0.74	$+0.51 / -0.81$
75–100	30.9 ± 3.3	$+4.6 / -4.4$	5.25 ± 0.54	$+0.65 / -0.62$
100–150	14.4 ± 1.3	$+1.2 / -1.24$	2.44 ± 0.21	$+0.13 / -0.13$
150–300	1.35 ± 0.23	$+0.35 / -0.30$	0.230 ± 0.038	$+0.055 /-0.046$

**Table 12:** Absolute and normalised unfolded differential $tq$ production cross-section as a function of $|y(\hat{t})|$ at particle level

$|y(\hat{t})|$	$\mathrm {d}\sigma (tq)/\mathrm {d}|y(\hat{t})|$		$(1/\sigma ) \mathrm {d}\sigma (tq)/\mathrm {d}|y(\hat{t})|$	
	[pb]		[10$$^{-3}$$]	
	Stat.	Syst.	Stat.	Syst.
0.00–0.15	9.00 ±0.45	$+0.43/ -0.43$	914 ± 43	$+19 /-18$
0.15–0.30	8.99 ±0.47	$+0.47 / -0.49$	913 ± 46	$+41/-43$
0.30–0.45	8.15 ± 0.48	$+0.59/ -0.60$	828 ± 46	$+44/-46$
0.45–0.70	6.88 ± 0.32	$+0.38 / -0.37$	699 ± 30	$+19 /-17$
0.70–1.00	5.70 ± 0.26	$+0.49 /-0.48$	579 ± 24	$+36 /-36$
1.00–1.30	3.47 ± 0.22	$+0.26/ -0.25$	353 ± 21	$+13 /-11$
1.30–2.20	1.61 ± 0.08	$+0.11/ -0.11$	164 ± 8	$+4 /-4$

**Table 13:** Absolute and normalised unfolded differential $\bar{t}q$ production cross-section as a function of $|y(\hat{t})|$ at particle level

$|y(\hat{t})|$	$\mathrm {d}\sigma (\bar{t}q)/\mathrm {d}|y(\hat{t})|$		$(1/\sigma ) \mathrm {d}\sigma (\bar{t}q)/\mathrm {d}|y(\hat{t})|$	
	[pb]		[10$$^{-3}$$]	
	Stat.	Syst.	Stat.	Syst.
0.00–0.15	6.65 ± 0.44	$+0.50 / -0.49$	1145 ± 70	$+57 / -55$
0.15–0.30	4.68 ± 0.43	$+0.41 / -0.43$	806 ± 71	$+51 / -57$
0.30–0.45	4.97 ± 0.42	$+0.40 / -0.39$	856 ± 69	$+44 / -40$
0.45–0.70	4.08 ± 0.29	$+0.34 / -0.33$	703 ± 46	$+38 / -39$
0.70–1.00	3.21 ± 0.23	$+0.27/-0.27$	553 ± 37	$+28 / -30$
1.00–1.30	2.30 ± 0.20	$+0.20/ -0.20$	396 ± 32	$+17/ -17$
1.30–2.20	0.76 ± 0.07	$+0.08 / -0.07$	132 ± 11	$+8 / -7$

**Fig. 19:**
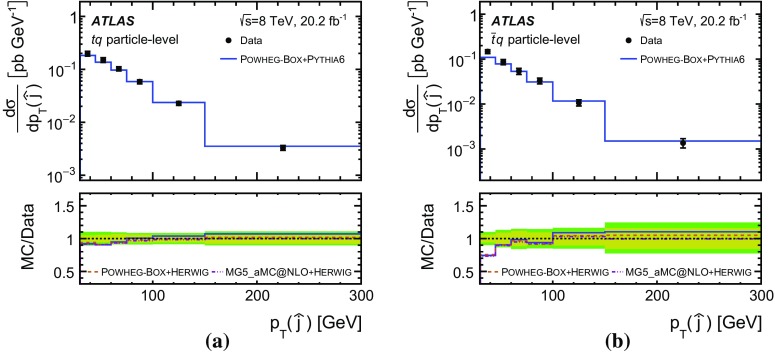
Absolute unfolded differential cross-sections as a function of $p_{\text {T}} (\hat{j})$ for **a** top quarks **b** top antiquarks. The unfolded distributions are compared to various MC predictions. The *vertical error bars* on the data points denote the total uncertainty. The *inner* (*yellow*) *band* in the *bottom part* of each figure represents the statistical uncertainty of the measurement, and the *outer* (*green*) *band* the total uncertainty.

**Fig. 20:**
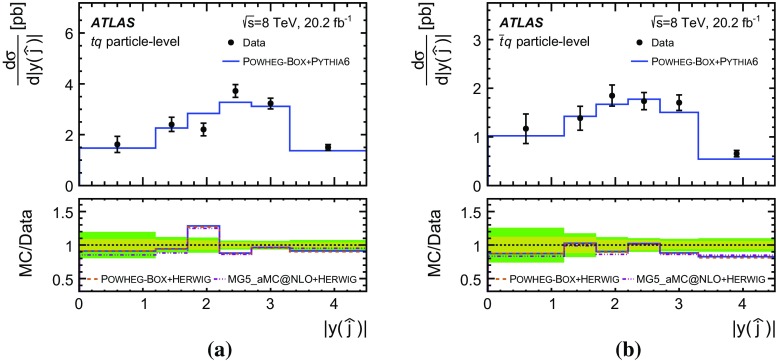
Absolute unfolded differential cross-sections as a function of $|y(\hat{j})|$ for **a** top quarks and **b** top antiquarks. The unfolded distributions are compared to various MC predictions. The *vertical error bars* on the data points denote the total uncertainty. The *inner* (*yellow*) *band* in the *bottom part* of each figure represents the statistical uncertainty of the measurement, and the *outer* (*green*) *band* the total uncertainty.

The absolute cross-sections for the untagged jet as a function of the same variables are shown in Figures 19 and 20 and both the absolute and normalised cross-sections are tabulated in Tables 14 ,15, 16, 17. The measurement as a function of $|y(\hat{j})|$ uses the neural network without $|\eta (j)|$, while all other measurements use the default network. The measured cross-sections are again well described by the predictions, although there is a tendency for the prediction to be somewhat harder than the data as a function of $p_{\text {T}} (\hat{j})$.

**Table 14:** Absolute and normalised unfolded differential $tq$ production cross-section as a function of $p_{\text {T}} (\hat{j})$ at particle level

$p_{\text {T}} (\hat{j})$	$\mathrm {d}\sigma (tq)/\mathrm {d}p_{\text {T}} (\hat{j})$		$(1/\sigma ) \mathrm {d}\sigma (tq)/\mathrm {d}p_{\text {T}} (\hat{j})$	
[GeV]	[fb GeV$^{-1}$]		[10$^{-3}$ GeV$^{-1}$]	
	Stat.	Syst.	Stat.	Syst.
30–45	199 ± 10	$+18 / -19$	20.1 ± 0.8	$+1.2 / -1.2$
45–60	151 ± 9	$+13 / -14$	15.26 ± 0.85	$+0.91 / -0.94$
60–75	102.3 ±7.0	$+6.8 / -5.8$	10.36 ± 0.69	$+0.46 / -0.35$
75–100	58.5 ± 3.5	$+3.5 / -3.9$	5.92 ± 0.35	$+0.24 / -0.27$
100–150	22.8 ± 1.3	$+1.4 / -1.4$	2.31 ± 0.13	$+0.11 / -0.11$
150–300	3.29 ± 0.26	$+0.24 / -0.22$	0.333 ± 0.026	$+0.019 / -0.015$

**Table 15:** Absolute and normalised unfolded differential $\bar{t}q$ production cross-section as a function of $p_{\text {T}} (\hat{j})$ at particle level

$p_{\text {T}} (\hat{j})$	$\mathrm {d}\sigma (\bar{t}q)/\mathrm {d}p_{\text {T}} (\hat{j})$		$(1/\sigma ) \mathrm {d}\sigma (\bar{t}q)/\mathrm {d}p_{\text {T}} (\hat{j})$	
[GeV]	[fb GeV$^{-1}$]		[10$^{-3}$ GeV$^{-1}$]	
	Stat.	Syst.	Stat.	Syst.
30–45	147 ± 9	$+12 / -12$	25.0 ± 1.2	$+1.1 / -1.0$
45–60	86.4 ± 7.8	$+8.3 / -8.5$	14.7 ±1.3	$+1.0 / -1.0$
60–75	54.2 ±6.2	$+5.1 / -6.0$	9.21 ± 1.03	$+0.68 / -0.88$
75–100	33.0 ± 3.1	$+3.7 / -3.9$	5.62 ±0.51	$+0.36 / -0.41$
100–150	10.7 ± 1.1	$+1.3 / -1.2$	1.82 ± 0.19	$+0.14 / -0.11$
150–300	1.36 ± 0.22	$+0.26 / -0.22$	0.231 ± 0.036	$+0.044 / -0.038$

**Table 16:** Absolute and normalised unfolded differential $tq$ production cross-section as a function of $|y(\hat{j})|$ at particle level

$|y(\hat{j})|$	$\mathrm {d}\sigma (tq)/\mathrm {d}|y(\hat{j})|$		$(1/\sigma ) \mathrm {d}\sigma (tq)/\mathrm {d}|y(\hat{j})|$	
	[pb]		[10$$^{-3}$$]	
	Stat.	Syst.	Stat.	Syst.
0.0–1.2	1.62 ± 0.14	$+0.28 / -0.28$	164 ± 12	$+22 / -22$
1.2–1.7	2.40 ± 0.18	$+0.22 / -0.20$	244 ± 17	$+15 / -11$
1.7–2.2	2.21 ± 0.15	$+0.19 / -0.20$	224 ± 15	$+10 / -11$
2.2–2.7	3.72 ± 0.16	$+0.19 / -0.19$	378 ± 16	$+16 / -16$
2.7–3.3	3.23 ± 0.13	$+0.16 / -0.17$	328 ± 13	$+15 / -15$
3.3–4.5	1.50 ± 0.06	$+0.10 / -0.10$	152.5 ± 6.0	$+9.2 /-9.3$

**Table 17:** Absolute and normalised unfolded differential $\bar{t}q$ production cross-section as a function of $|y(\hat{j})|$ at particle level

$|y(\hat{j})|$	$\mathrm {d}\sigma (\bar{t}q)/\mathrm {d}|y(\hat{j})|$		$(1/\sigma ) \mathrm {d}\sigma (\bar{t}q)/\mathrm {d}|y(\hat{j})|$	
	[pb]		[10$$^{-3}$$]	
	Stat.	Syst.	Stat.	Syst.
0.0–1.2	1.17 ± 0.14	$+0.27 / -0.27$	205 ± 20	$+31 /-31$
1.2–1.7	1.39 ± 0.17	$+0.18 / -0.18$	243 ± 27	$+14 /-16$
1.7–2.2	1.85 ± 0.14	$+0.16 / -0.16$	324 ± 25	$+20 / -17$
2.2–2.7	1.73 ± 0.13	$+0.12 / -0.12$	305 ± 22	$+20 /-19$
2.7–3.3	1.70 ± 0.10	$+0.12 / -0.12$	299 ± 19	$+26 /-26$
3.3–4.5	0.655 ± 0.04	$+0.053 / -0.051$	115 ± 8	$+11 /-11$

In general, the main sources of uncertainty in the differential cross-sections are similar to those for the fiducial cross-section measurements: the JES calibration and uncertainties associated with the modelling of both the signal and the $t\bar{t}$ background. The background normalisation uncertainty is typically about half of the total systematic uncertainty, while the statistical uncertainty in each bin is similar to the total systematic uncertainty for the absolute cross-section measurements. For the normalised cross-sections, the luminosity and $b/\bar{b}$ efficiency uncertainties cancel and the size of many other systematic uncertainty contributions is reduced. Uncertainties due to the unfolding are small compared to the total uncertainty.

### Parton-level cross-sections

**Fig. 21:**
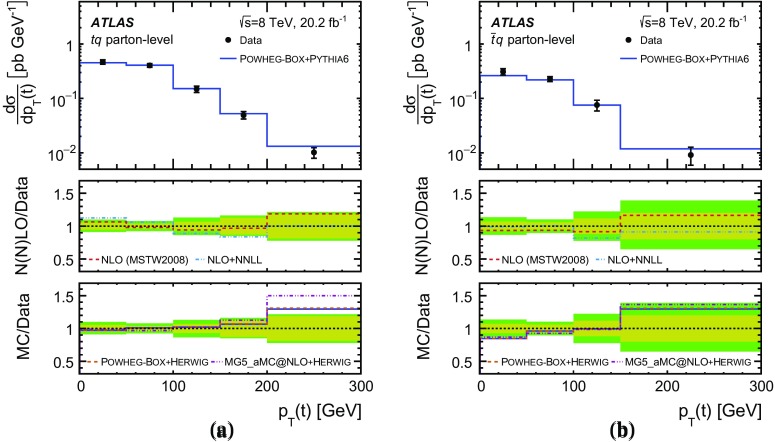
Absolute unfolded differential cross-sections as a function of $p_{\text {T}} (t)$ for **a** top quarks and **b** top antiquarks. The unfolded distributions are compared to QCD NLO and NLO + NNLL calculations as well as various MC predictions. The *vertical error bars* on the data points denote the total uncertainty. The *dashed* (*red*) *line* in the central distribution shows the NLO prediction calculated using MCFM. The *dash-dot* (*blue*) *line* is the NLO + NNLL prediction [25]. The *bottom* distribution compares the data with the MC predictions from Powheg-Box (*orange dashed line*) and MadGraph5_aMC@NLO (*purple dash-dotted line*). The *inner* (*yellow*) *band* in the *bottom* part of each figure represents the statistical uncertainty of the measurement, and the outer (*green*) *band* the total uncertainty.

**Fig. 22:**
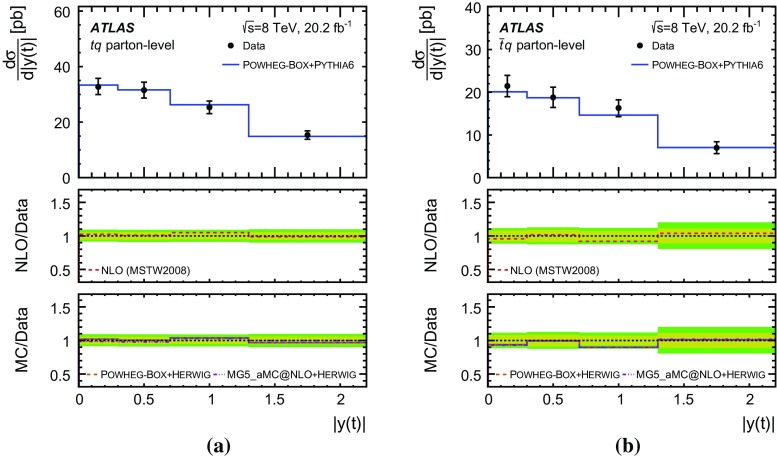
Absolute unfolded differential cross-sections as a function of $|y(t)|$ for **a** top quarks and **b** top antiquarks. The unfolded distributions are compared to a QCD NLO calculation and various MC predictions The *vertical error bars* on the data points denote the total uncertainty. The *dashed* (*red*) *line* in the central distribution shows the NLO prediction calculated using MCFM. The *bottom* distribution compares the data with the MC predictions from Powheg-Box (*orange dashed line*) and MadGraph5_aMC@NLO (*purple dash-dotted line*). The *inner* (*yellow*) *band* in the *bottom* part of each figure represents the statistical uncertainty of the measurement, and the outer (*green*) *band* the total uncertainty

Differential cross-sections for the top quark and antiquark at parton level are measured as a function of $p_{\text {T}} (t)$ and $y(t)$. The absolute cross-sections are shown in Figs. 21 and 22 and the numerical values for both the absolute and normalised cross-sections are given in Tables 18, 19, 20, 21. The measured cross-sections are compared to both NLO QCD predictions as well as the same MC predictions used for the comparison of the particle-level cross-sections. A calculation at NLO + NNLL QCD is available for the top-quark $p_{\text {T}}$  [89]. This is compared to the data in Fig. 21. All predictions agree well with the data, with the same tendency for almost all MC predictions to be somewhat harder than the data as a function of $p_{\text {T}} (t)$. The NLO + NNLL prediction describes the data better than the MC predictions as a function of $p_{\text {T}} (t)$.

**Table 18:** Absolute and normalised unfolded differential $tq$ production cross-section as a function of $p_{\text {T}} (t)$ at parton level

$p_{\text {T}} (t)$	$\mathrm {d}\sigma (tq)/\mathrm {d}p_{\text {T}} (t)$		$(1/\sigma ) \mathrm {d}\sigma (tq)/\mathrm {d}p_{\text {T}} (t)$	
[GeV]	[fb GeV$^{-1}$]		[10$^{-3}$ GeV$^{-1}$]	
	Stat.	Syst.	Stat.	Syst.
0–50	467 ± 25	$+34 / -39$	8.57 ± 0.33	$+0.32 / -0.43$
50–100	404 ± 15	$+28 / -27$	7.42 ± 0.32	$+0.47 /-0.40$
100–150	149 ± 10	$+17 / -18$	2.73 ± 0.18	$+0.27 / -0.29$
150–200	49.2 ± 6.3	$+5.0 / -4.1$	0.90 ± 0.12	$+0.08 / -0.07$
200–300	10.2 ± 1.9	$+1.2 / -1.3$	0.187 ± 0.035	$+0.019 /-0.022$

**Table 19:** Absolute and normalised unfolded differential $\bar{t}q$ production cross-section as a function of $p_{\text {T}} (t)$ at parton level

$p_{\text {T}} (t)$	$\mathrm {d}\sigma (\bar{t}q)/\mathrm {d}p_{\text {T}} (t)$		$(1/\sigma ) \mathrm {d}\sigma (\bar{t}q)/\mathrm {d}p_{\text {T}} (t)$	
[GeV]	[fb GeV$^{-1}$]		[10$^{-3}$ GeV$^{-1}$]	
	Stat.	Syst.	Stat.	Syst.
0–50	310 ± 21	$+36 / -35$	9.67 ± 0.48	$+0.77 / -0.76$
50–100	228 ± 13	$+19 / -20$	7.11 ± 0.47	$+0.49 /-0.51$
100–150	76 ± 9	$$+14 / -14$$	2.36 ± 0.27	$$+0.45 / -0.46 $$
150–300	9.1 ± 1.8	$+3.1 / -2.6$	0.284 ± 0.057	$+0.089 /-0.076$

**Table 20:** Absolute and normalised unfolded differential $tq$ production cross-sections as a function of $|y(t)|$ at parton level

$$|y(t)| $$	$$\mathrm {d}\sigma (tq)/\mathrm {d}|y(t)| $$		$$(1/\sigma ) \mathrm {d}\sigma (tq)/\mathrm {d}|y(t)| $$	
	[pb]		[10$$^{-3}$$]	
	Stat.	Syst.	Stat.	Syst.
0.0–0.3	32.7 ± 1.8	$+2.5 / -2.1$	636 ± 35	$+47 /-39$
0.3–0.7	31.5 ± 1.8	$+2.2 / -2.4$	613 ± 34	$+31 /-33$
0.7–1.3	25.3 ± 1.3	$+1.9 / -1.9$	492 ± 24	$+26 / -27$
1.3–2.2	15.4 ± 0.9	$$+1.2 / -1.2 $$	299 ± 14	$$+14 / -15$$

**Table 21:** Absolute and normalised unfolded differential $\bar{t}q$ production cross-sections as a function of $|y(t)|$ at parton level

$$|y(t)| $$	$$\mathrm {d}\sigma (\bar{t}q)/\mathrm {d}|y(t)| $$		$$(1/\sigma ) \mathrm {d}\sigma (\bar{t}q)/\mathrm {d}|y(t)| $$	
	[pb]		[10$$^{-3}$$]	
	Stat.	Syst.	Stat.	Syst.
0.0–0.3	21.5 ± 1.7	$+1.8 / -1.9$	714 ± 55	$+41/ -46$
0.3–0.7	18.8 ± 1.6	$+1.7 / -1.7$	626 ± 53	$+46/-46$
0.7–1.3	16.3 ± 1.2	$+1.6 /-1.6$	543 ± 37	$+44 / -43$
1.3–2.2	7.0 ± 0.8	$+1.2 / -1.1$	233 ± 23	$+30 /-29$

## Conclusion

Measurements of *t*-channel single top-quark production using data collected by the ATLAS experiment in *pp* collisions at 8 TeV at the LHC are presented. The data set corresponds to an integrated luminosity of 20.2 fb$^{-1}$. An artificial neural network is used to separate signal from background. Total and fiducial cross-sections are measured for both top quark and top antiquark production. The fiducial cross-section is measured with a precision of 5.8% (top quark) and 7.8% (top antiquark), respectively. In addition, the cross-section ratio of top-quark to top-antiquark production is measured, resulting in a precise value to compare with predictions, $R_t = 1.72 \pm 0.09$. The total cross-section is used to extract the *Wtb* coupling: $f_{\text {LV}} \cdot |V_{tb}| = 1.029\pm 0.048$, which corresponds to $|V_{tb}|>0.92$ at the 95 % confidence level, when assuming $f_{\text {LV}} =1$ and restricting the range of $|V_{tb}|$ to the interval [0, 1].

Requiring a high value of the neural-network discriminant leads to relatively pure *t*-channel samples, which are used to measure differential cross-sections for both $tq$ and $\bar{t}q$ production. Differential cross-sections as a function of the transverse momentum and absolute value of the rapidity of the top quark, the top antiquark, as well as the accompanying jet from the *t*-channel scattering are measured at particle level. The measurements of cross-sections as a function of the accompanying-jet transverse momentum and absolute value of the rapidity extend previous results, which only measured top-quark and top-antiquark distributions. Differential cross-sections as a function of the transverse momentum and rapidity of the top quark and top antiquark are also measured at parton level. All measurements are compared to different Monte Carlo predictions as well as to fixed-order QCD calculations where these are available. The SM predictions provide good descriptions of the data.

## Footnotes

1 Events involving $W\rightarrow \tau \nu$ decays with a subsequent decay of the $\tau$ lepton to either $e\nu _e\nu _\tau$ or $\mu \nu _\mu \nu _\tau$ are included in the signal.

2 ATLAS uses a right-handed coordinate system with its origin at the nominal interaction point (IP) in the centre of the detector and the *z*-axis along the beam pipe. The *x*-axis points from the IP to the centre of the LHC ring, and the *y*-axis points upwards. Cylindrical coordinates $(r,\phi )$ are used in the transverse plane, $\phi$ being the azimuthal angle around the *z*-axis. The pseudorapidity is defined in terms of the polar angle $\theta$ as $\eta = -\ln \tan (\theta /2)$. Angular distance is measured in units of $\Delta R \equiv \sqrt{(\Delta \eta )^{2} + (\Delta \phi )^{2}}$.

3 The primary vertex is defined as the vertex with the largest $\sum p_{\text {T}} ^2$ of the associated tracks.
